# Simulations of tumor growth and response to immunotherapy by coupling a spatial agent-based model with a whole-patient quantitative systems pharmacology model

**DOI:** 10.1371/journal.pcbi.1010254

**Published:** 2022-07-22

**Authors:** Alvaro Ruiz-Martinez, Chang Gong, Hanwen Wang, Richard J. Sové, Haoyang Mi, Holly Kimko, Aleksander S. Popel

**Affiliations:** 1 Department of Biomedical Engineering, Johns Hopkins, University School of Medicine, Baltimore, Maryland, United States of America; 2 Clinical Pharmacology & Quantitative Pharmacology, AstraZeneca, Gaithersburg, Maryland, United States of America; 3 Department of Oncology and Sidney Kimmel Comprehensive Cancer Center, Johns Hopkins University, Baltimore, Maryland, United States of America; H. Lee Moffitt Cancer Center & Research Institute, UNITED STATES

## Abstract

Quantitative systems pharmacology (QSP) models and spatial agent-based models (ABM) are powerful and efficient approaches for the analysis of biological systems and for clinical applications. Although QSP models are becoming essential in discovering predictive biomarkers and developing combination therapies through *in silico* virtual trials, they are inadequate to capture the spatial heterogeneity and randomness that characterize complex biological systems, and specifically the tumor microenvironment. Here, we extend our recently developed spatial QSP (spQSP) model to analyze tumor growth dynamics and its response to immunotherapy at different spatio-temporal scales. In the model, the tumor spatial dynamics is governed by the ABM, coupled to the QSP model, which includes the following compartments: central (blood system), tumor, tumor-draining lymph node, and peripheral (the rest of the organs and tissues). A dynamic recruitment of T cells and myeloid-derived suppressor cells (MDSC) from the QSP central compartment has been implemented as a function of the spatial distribution of cancer cells. The proposed QSP-ABM coupling methodology enables the spQSP model to perform as a coarse-grained model at the whole-tumor scale and as an agent-based model at the regions of interest (ROIs) scale. Thus, we exploit the spQSP model potential to characterize tumor growth, identify T cell hotspots, and perform qualitative and quantitative descriptions of cell density profiles at the invasive front of the tumor. Additionally, we analyze the effects of immunotherapy at both whole-tumor and ROI scales under different tumor growth and immune response conditions. A digital pathology computational analysis of triple-negative breast cancer specimens is used as a guide for modeling the immuno-architecture of the invasive front.

## Author summary

Spatial heterogeneity is a hallmark of cancer, thus the ability to quantify the complexity of the tumor microenvironment is an important goal of computational modeling. We present a hybrid computational modeling platform, spQSP, to extend quantitative systems pharmacology (QSP) models of immuno-oncology into the spatial dimension by combining them with spatial agent-based models (ABM). We focus on several methodological and biological aspects of modeling cancer. First, the coupling of deterministic ordinary differential equation based whole-patient QSP model and stochastic, spatial agent-based model of tumor. Second, we focus on the region at the edge of the tumor called the Invasive Front (IF). We introduce a quantitative definition of IF consistent with pathologists’ definition and present examples of its geometry. Third, we apply the model to immunotherapy of triple-negative breast cancer and compare the simulations of spatial distribution of immune cells (e.g., CD8+ and FoxP3+ T cells) with our recent digital pathology quantitative analysis of patients’ specimens. Thus, the computational platform combines the power of QSP models with spatial ABM thus opening the way to utilizing the immense information about the tumor microenvironment contained in multiplexed pathology specimens. This modeling platform enables conducting virtual clinical trials and biomarker discovery for cancer immunotherapies.

## Introduction

The study of the tumor microenvironment (TME) is essential for understanding the biological mechanisms involved in the tumor growth, progression, and dissemination processes as well as the role of the host immune system [1,2]. The TME consists of innate and adaptive immune cell subpopulations, fibroblasts, adipocytes, immune-inflammatory cells, and blood and lymphatic vascular networks [3,4,5]. A variety of processes involve cells that constitute the TME: recruitment of immune cells from blood vessels, migration, proliferation, differentiation, expansion, apoptosis, etc. Such level of intra-tumoral complexity and the wide range of patients with different anti-tumor immune responses hinder the ability to predict cancer progression [6,7,8]. Thus, understanding the unique profile of T cells in that heterogeneous microenvironment as well as defining the prognostic role of each cell type are crucial for understanding of how the immune microenvironment contributes to cancer progression. On the other hand, agents commonly known as immune checkpoint blockers (ICB) or immune checkpoint inhibitors (ICI) enhance antitumor response by targeting some specific components in the TME [9,10]. Over the last decade, immunotherapies with ICBs have demonstrated promising clinical outcomes and increase in survival rates in different types of advanced cancer [11,12,13].

Quantitative systems pharmacology (QSP) models have become vital in discovering predictive biomarkers and helped develop and test combination therapies through in silico virtual trials [14,15,16,17]. QSP models have been successfully applied to the study of different cancers as well as the development of immunotherapies, e.g., breast [18], lung [19], melanoma [20], colorectal [21]. Yet, although these models can represent the complexity of the biology involved in the tumor growth, the tumor microenvironment, the immune response, or the antibody pharmacokinetics and pharmacodynamics, their efficiency is limited. QSP models are non-spatial and deterministic, and they lack the ability to represent the heterogeneity and stochasticity of the tumor as well as the spatial distribution of the elements that comprise the tumor microenvironment [22,23,24]. Recent data from cancer studies prove the importance of modeling spatio-temporal features of cancer progression in order to understand the key role of the immune system and develop more effective combination immunotherapies [25,26,27,28,29]. Agent-based models, where entities called agents act and interact according to a set of rules, deterministic or stochastic, are excellent tools to represent the elements and processes that characterize the TME and the effects of immunotherapies with ICBs [30,31,32,33,34,35,36,37].

Spatial models have been widely used to study the importance of the heterogeneity of the tumor microenvironment on solid tumor growth and morphology and the implications for cancer therapy. For instance, spatial analysis and nonlinear simulations have been performed to characterize invasiveness and fingering/fragmentation mechanisms of solid tumors [38]. A three-dimensional multispecies mixture model has also probed that spatial patterning of cancer stem cells and externally applied signaling factors play a central role in the development or suppression of fingering structures in normal and cancer colon organoids [39]. Additionally, agent-based modeling has been used to identify adaptive dosing strategies that control invasiveness of heterogeneous tumors to reduce resistance and recurrence [40]. Regarding cancer immunotherapy, an extensive variety of modeling approaches have been developed: data-driven top-down vs mechanistic bottom-up, simplistic vs detailed, continuous vs discrete, and hybrid [41]. Nevertheless, only stochastic models have the ability to represent the complex spatial dynamics of cancer and immune cells that is key in the optimization of viral dosing and production of effective treatment outcomes [42,43].

Multiscale hybrid models are powerful tools that combine deterministic and stochastic scenarios that handle a wide range of spatio-temporal scales [44,45]. Here, we extend a novel and recently published spatial QSP model (spQSP) [36,37], where a whole-patient ordinary differential equations-based QSP model and a spatial ABM, each representing part of a whole tumor, are combined, to study the dynamics of tumor growth and the response to immunotherapy at different spatial and temporal scales. Different types of cancer cells, T cells, and myeloid-derived suppressor cells (MDSCs) in ABM are represented as agents that move and react, conforming to a set of probabilistic rules, whereas QSP model defines an average number of cells that changes over time. Unlike previous studies [36,37], where the state of the species in the tumor is defined by combining the outcomes from QSP model and ABM, the presented approach replaces the spatially homogeneous QSP representation of cells in the tumor by the spatial ABM representation after applying a scaling factor. The QSP temporal dynamics of cells in the tumor is redefined in terms of propensity functions and probabilities, which establishes a simple methodology to transform QSP models into their equivalent spatial representations and guarantees a consistent coupling between QSP model and ABM. Although the ABM provides information at a microscopic level, this mesoscopic coupling approach enables us to use spQSP as a coarse-grained model to represent the entire system at the tumor scale. Moreover, although this extended version does not yet include tumor vasculature explicitly, it introduces a dynamic recruitment of cells from blood based on the local concentration of cancer cells in order to characterize T cell hotspots at the tumor scale and their density profiles at the invasive front. A digital pathology computational analysis of triple-negative breast cancer specimens is used as a guide for modeling the immuno-architecture in this study [28].

This study is an extension of our recent spQSP platform development [36,37]. Our specific goals in this study are two-fold: (a) To present the methodology of coupling QSP and ABM models and demonstrate its application on several examples; (b) to focus on the topic of the invasive front in triple-negative breast cancer and demonstrate that the model is in agreement with our previously published analysis of pathology samples [28].

## Material and methods

Our hybrid spQSP model applied to triple-negative breast cancer (TNBC) is composed of two parts: a QSP model based on ordinary differential equations and a spatial agent-based model. The former is used to study the anti-tumor immune response by representing the human body as a four-compartment system: central (blood system), tumor, tumor-draining lymph node, and peripheral (the rest of the organs and tissues); the latter describes the spatio-temporal evolution of the tumor microenvironment in order to study the level of heterogeneity that characterizes this type of tumors. The QSP model, and more specifically, the tumor compartment from QSP is combined with the ABM of tumor resulting in the spatial QSP which we refer to as spQSP.

### Quantitative systems pharmacology model

**Fig 1:**
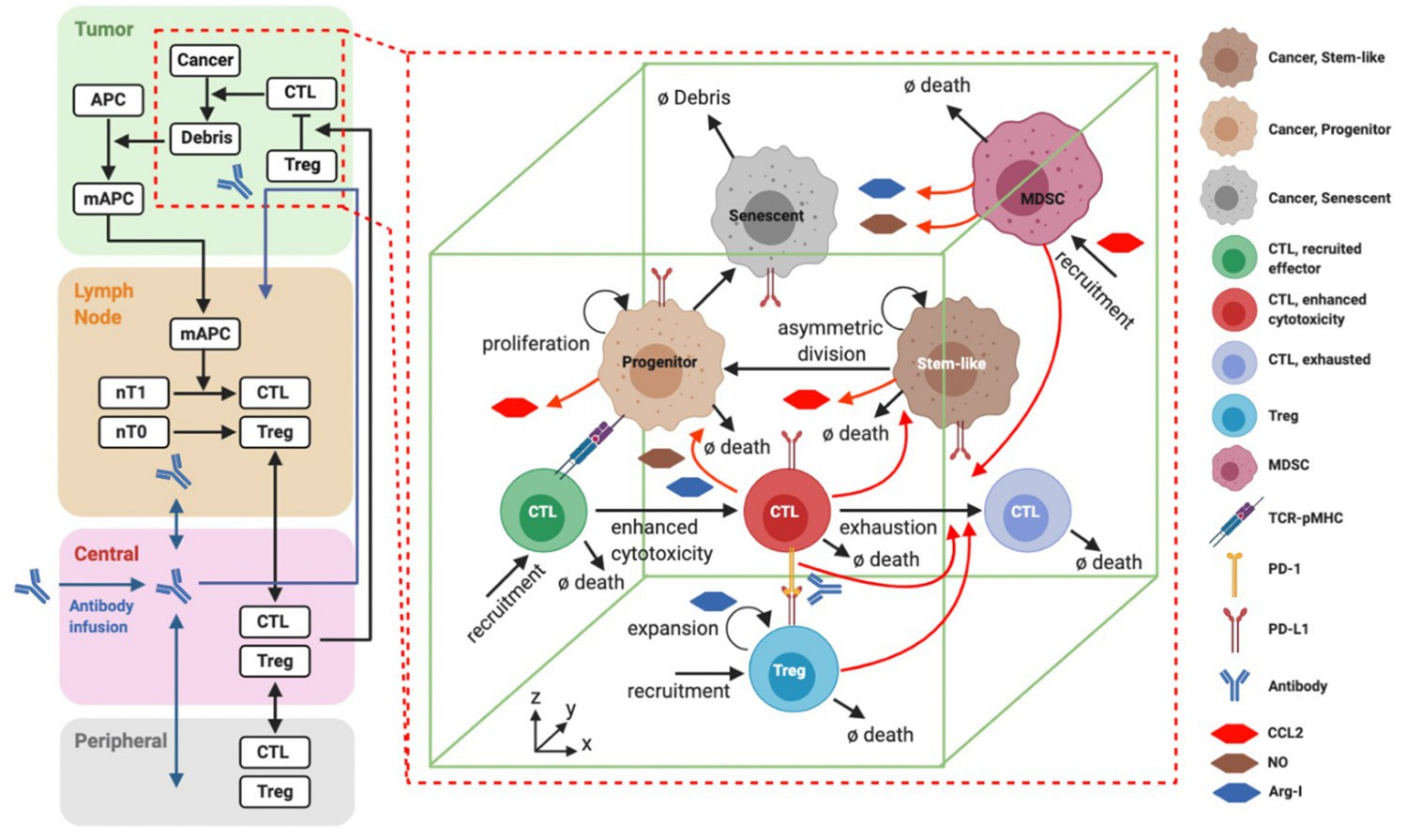
Diagram of the spQSP model. Left: Compartmental representation of the whole patient by QSP model. Right: Spatial representation of the tumor by ABM. Some deterministic species and reactions from the QSP tumor compartment are replaced with equivalent agents and stochastic reactions in ABM. The antigen module, however, is not explicitly represented in ABM. It consists of antigen-presenting cells that mature after taking antigens from the tumor compartment to transport them through lymphatic vessels to the tumor-draining lymph node compartment. Once there, they prime naïve cytotoxic T lymphocytes (CTL) and regulatory T cells (Treg) that clonally expand in the tumor draining lymph nodes, intravasate, circulate through the central compartment (blood system), and extravasate into the tumor microenvironment as described in [1]. The figure has been created with Biorender.

The QSP part of our hybrid model has been recently developed by Wang et al. [46]; the model is related to other models from our laboratory [18,19,20,21,47,48,49,50]. It is composed of eight modules that correspond to different types of cell/species: cancer cells, effector T cells, regulatory T cells, MDSCs, antigen-presenting cells, antigens, immune checkpoint ligands and receptors, and therapeutic agents. The dynamics of the major species is illustrated in the left side of Fig 1 and the temporal evolution of their concentrations is obtained by solving a system of 120 ODEs and 39 algebraic equations. Wang et al. [46] implemented the SimBiology toolbox in MATLAB and generated a Systems Biology Markup Language (SBML) file with the full information about the reaction fluxes, algebraic equations, and model parameters. We converted this file to C++ programming language by using a converter tool developed in Python language to be able to combine it with ABM that is entirely developed in C++ language.

Without modifying the differential and algebraic equations from Wang et al. [46] model, we have recalibrated some parameters to obtain a set of simulations that agree with the clinical observations; specifically, on the number of responders to anti-PD-1 treatment and T cell densities in the tumor [51]. Section A.1 of the S1 Supplementary Material includes QSP solutions for 100 cases without and with anti-PD-1 treatment after recalibration. All parameter values, reactions, and expressions of the QSP model are listed in S1 Table.

### Spatial quantitative systems pharmacology model

#### Description of the agent-based model

The ABM proposed in this study replaces the tumor compartment of the QSP model by scaling the number of cells and representing either a small version of the tumor or a sample volume of it (region of interest, ROI). This ABM is a significant extension of a previous model developed by Gong et al. [31] with additional species and rules. Also, it is more detailed than the QSP model in terms of species since we include different types of cancer cells (stem-like, progenitor, and senescent) and T cells (effector, cytotoxic, exhausted) (Fig 1). A related spQSP model has recently been published and applied to non-small cell lung cancer (NSCLC) [36]; it was coupled with our previously published QSP model for NSCLC [19]. The current model also differs from the model developed by Zhang et al. for triple-negative breast cancer [37] where the emphasis was on incorporating single-cell RNA sequencing data into the spQSP model. There are important differences between the models, particularly in how the QSP model and ABM are coupled and also in the representation of certain species, thus here we present the complete model formulation for clarity and reproducibility.

The three-dimensional grid is defined as a parallelepiped composed of voxels where the cells (or agents) are located. For this study we assume voxels of size 20x20x20 microns. Cell dynamics in the grid takes place according to the following rules:

- Due to their relatively large cellular volumes, only one cancer cell or MDSC is allowed to occupy one voxel.
- Up to eight T cells can occupy one voxel but only one T cell can coexist with a cancer cell or a MDSC.
- All cells have a probability of movement assigned so they can randomly migrate to adjacent voxels every time step.
- A cell can only interact with cells that surround them in the spatial grid, i.e., cells that occupy the same voxel and the 26 neighboring voxels.
- No-flux boundary conditions are imposed at the edges of the 3D parallelepiped (which in this study is assumed to be 150x150x150 voxels to represent the entire tumor and 200x200x20 voxels to study part of the tumor; these dimensions can be readily changed depending on needs and computer resources).

**Fig 2:**
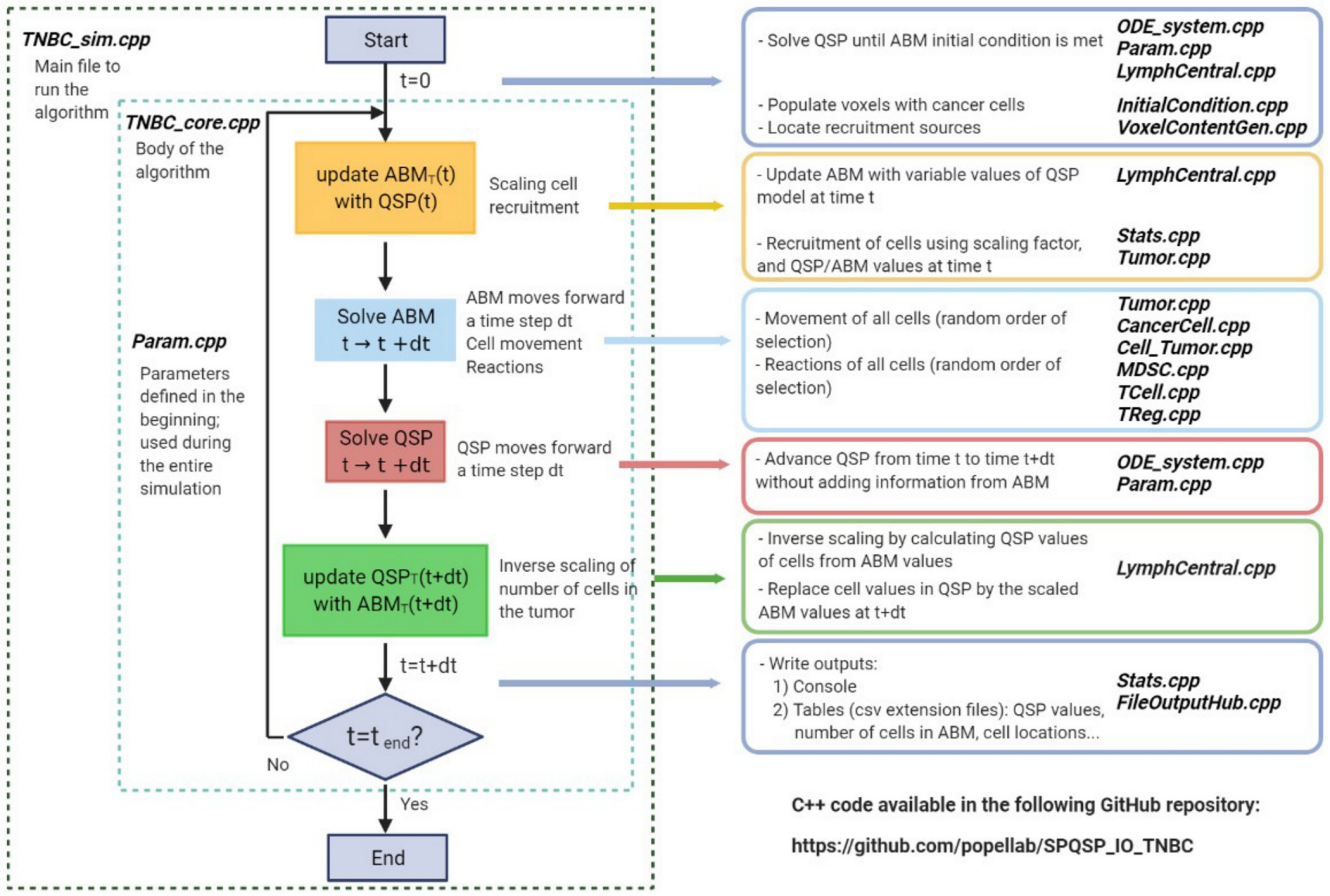
Workflow and description of the spatial QSP model explaining how the QSP model and ABM are coupled. Left: Workflow of the calculation steps in the spQSP algorithm. Right: Breakdown of each step and list of the main C++ files used in the step. The figure has been created with Biorender.

Fig 2 shows a workflow of the spatial QSP algorithm. The QSP model is initialized before ABM and starts from a single cancer cell (unless ABM is assumed to start from a single cancer stem-like cell in order to initialize QSP model and ABM at the same time) or a spatial distribution of cancer cells, e.g., normal distribution. Then, a specific diameter pre-selected from a random distribution is used to calculate the initial tumor volume assuming a spherical tumor. Once the tumor reaches that predefined volume in the QSP model, the values of the species divided by a scaling factor are set as the initial conditions in the ABM model. Alternatively, it is possible to initialize ABM before the predefined tumor volume in the QSP model is reached and before a significant number of T cells and MDSCs are recruited into the QSP tumor compartment not to enforce initial spatial distributions of such cells in ABM, e.g. when a normal distribution of cancer cells is imposed as the initial condition, but the tumor is still so small that T cells and MDSCs are not present yet. Once initialized, some of the QSP model values at a time *t* are used in the reaction rates in ABM, e.g., the recruitment rates of T cells and MDSCs to represent the ABM scaled version of the real-size tumor or a fraction of it; the scaling equations will be presented in the section below and Section A.2 of the S1 Supplementary Material as part of the QSP-ABM coupling description. Then, ABM assumes that cells move to adjacent voxels and react during the time step *dt*. After all reactions take place, the values of QSP model variables at time *t* are used to advance the QSP part to a time *t* + *dt* and the number of cells in ABM is inversely scaled to update the total number of cells in the QSP tumor compartment at *t* + *dt*; the inverse scaling equations will also be presented as part of the QSP-ABM coupling description and Section A.2 of the S1 Supplementary Material. Thus, all species in QSP model are updated to their state at *t* + *dt* and the algorithm repeats the calculations for the subsequent time step.

### Representation of species in the ABM

#### Cancer cells

Unlike QSP model, ABM classifies cancer cells into three categories: cancer stem-like cells (CSCs), progenitor cells (PCs), and senescent cells (SCs). Following the cancer cell rules proposed by Norton et al. [30,52], CSCs division happens either symmetrically or asymmetrically. By defining a probability of division, *k*, ABM determines if a CSC divides to two daughter CSCs or to a CSC and a PC. All CSCs are assumed to divide indefinitely, i.e., there is no limit to the number of divisions. On the contrary, a maximum number of divisions, *d*_*max*_, is specified for PCs before they transition to the senescent state. Finally, SCs do not have the ability to divide and end up dying at a predefined death rate, *μ*.

#### CD8+ T cells

While QSP model only distinguishes between effector T cells and exhausted/suppressed T cells, ABM also divides the former into two subcategories: effector and cytotoxic T cells. Effector T cells are recruited from blood (QSP central compartment) at random spatial locations in ABM that represent entry points in the tumor microvasculature. When these cells are in the proximity of cancer cells, they get activated and become cytotoxic, i.e., they gain the ability to kill cancer cells. Subsequently, T cells either get exhausted or die. One type of exhaustion occurs when the programmed cell death proteins 1 (PD-1), expressed on T cells surfaces, bond with the programmed death-ligands 1 (PD-L1) on the surroundings cancer cells and T cells. The other type of exhaustion takes place when neighboring regulatory T cells inhibit the cytotoxic function of effector T cells. Once the latter get exhausted, their cytotoxic capability gets suppressed and become innocuous for cancer cells. Henceforth, we refer to the sum of effector, cytotoxic, and exhausted/suppressed T cells as CD8+ T cells.

#### Regulatory T cells

Regulatory T cells (Tregs) are recruited into the tumor from blood and expand in the presence of Arginase-I (Arg-I). They have the ability of exhausting cytotoxic T cells when they encounter each other. Like effector T cells, regulatory T cells die at a specific death rate. Henceforth, we interchangeably use the terms *regulatory T cells* and *FoxP3+ T cells* for the same type of cells.

#### Myeloid-derived suppressor cells

MDSCs get into the tumor from blood at the entry points in ABM. In addition to a baseline recruitment, MDSCs also get recruited by the chemokine CCL2 secreted by cancer cells. MDSCs secret the cytokine Arg-I and nitric oxide (NO) that inhibit the killing of cancer cells by cytotoxic effector T cells. Additionally, Arg-I activates the regulatory T cell’s expansion mechanism in the tumor.

#### Other species

Concentrations of CCL2, NO, Arg-I, and PDL1-PD1 are calculated in the QSP model and are assumed to be constant over the entire ABM domain.

### Representation of cell migration in the ABM

The probability of migration of a cell through the three-dimensional grid is dependent on the migration rate and it is defined in Section A.2 of the S1 Supplementary Material.

### Representation of reaction rates in the ABM

#### Cancer cells growth and death

Following [36], we formulate an ordinary differential equation (ODE) version of the ABM rules for cancer cell growth dynamics to keep consistency between the QSP model and ABM. Calculations are summarized in Section A.2 of the S1 Supplementary Material.

#### Decay reaction rates

Taking the reaction rates from the ODEs in the QSP model, we define propensities per cell and probabilities for the reactions where the number of cells in the tumor decays in Section A.2 of the S1 Supplementary Material. This approach is applied to five processes: death of cancer cells by cytotoxic T cells, death of regulatory T cells, cytotoxic T cell exhaustion (by regulatory T cells or from PD-L1 interaction), death of effector and cytotoxic T cells, and death of MDSCs.

#### Recruitment reaction rates

Recruitment rates describe the number of T cells and MDSCs that get recruited in the tumor from blood. The dynamics of this process is described in terms of probabilities and propensities defined in Section A.2 of the S1 Supplementary Material. This approach is applied to four processes: recruitment of regulatory T cells, recruitment of effector T cells, base recruitment of MDSCs, and recruitment of MDSCs by CCL2.

Section A.3 of the S1 Supplementary Material includes the characterization of the region with maximum probability of recruitment based on the analysis of the invasive front of a tumor.

#### Expansion reaction rate

The expansion of regulatory T cells by Arg-I is also described in terms of probabilities in Section A.2 of the S1 Supplementary Material.

### QSP-ABM coupling

#### Scaling: the number of cells in the QSP model is used to update ABM

Defining the number of cells of a species in the QSP tumor as *S*_*T*_ and the number of cells of the same species in the ABM tumor as *S*_*T’*_, we express the variation of number of cells in time as follows

$$\frac{dS_{T}}{dt}=k_{r}S_{T}$$ 1

where *k*_*r*_ is a generic reaction rate. In our model, we assume that propensities and probabilities of reaction are defined from reaction rates in the QSP model. Consequently, we can define a constant scaling factor *γ* between the number of cells in the tumor compartment in the QSP model and ABM such that

$$S_{T}=\gamma S_{T^{\prime }}$$ 2

therefore, ABM still preserves the same dynamics in time than the QSP model,

$$\frac{dS_{T}}{dt}=k_{r}S_{T}\to \frac{d(\gamma S_{T\prime })}{dt}=k_{r}(\gamma S_{T^{\prime }})\to \frac{dS_{T\prime }}{dt}=k_{r}S_{T\prime }.$$ 3

Thus, from Eq (2), if there are no cells of the species *S*_*T’*_ in ABM at a time *t*, i.e. *S*_*T’*,*t*_ = 0, the estimated number of cells in ABM after a time step *τ*, *S*_*T’*,*t+τ*_, is dependent on the factor *γ*, such that

$$S_{T^{\prime },t+\tau }=\left\lfloor \frac{S_{T,t+\tau }}{\gamma }\right\rfloor ,$$ 4

where *S*_*T*,*t*+*τ*_ is the number of cells in the QSP tumor compartment at a time *t* + *τ*. This condition is applied to T cells and MDSCs such that

$$T_{eff}\prime =\left\lfloor \frac{T_{1}}{\gamma }\right\rfloor ,T_{exh}\prime =\left\lfloor \frac{T_{exh}}{\gamma }\right\rfloor ,T_{0}\prime =\left\lfloor \frac{T_{0}}{\gamma }\right\rfloor ,MDSC\prime =\left\lfloor \frac{MDSC}{\gamma }\right\rfloor ,$$ 5

where *T*_1_, *T*_*exh*_, *T*_0_, and *MDSC*, refer to the number of effector T cells, exhausted/suppressed T cells, regulatory T cells, and MDSCs in the QSP model, respectively, and *T*_*eff*_′, *T*_*exh*_′, *T*_0_′, and *MDSC*′, refer to the number of effector T cells, exhausted/suppressed T cells, regulatory T cells, and MDSCs in ABM, respectively,

Eq (2) is also used to estimate the number of cells recruited by ABM from the QSP central compartment every time step. The probabilities of cell recruitment in the tumor are scaled when the propensities of recruitment defined in Section A.2 of the S1 Supplementary Material are divided by the scaling factor *γ*. Therefore, a generic probability of recruitment can be expressed as

ABMprobability:

$$p_{S_{T^{\prime }},rec}=1-exp\left(-\frac{a_{S_{T^{\prime }},rec}}{\gamma }\tau \right)$$ 6

where $a_{S_{T^{\prime }},rec}$ denotes the propensity of recruitment of a cell of the species *S*_*T’*_ in the ABM tumor. Consequently, a T cell or MDSC is recruited if the condition $p_{S_{T^{\prime }},rec}>\xi_{U}$ is met, where *ξ*_*U*_ is any random number from a uniform distribution on the interval [0,1].

#### Inverse scaling: The number of cells in ABM is used to update the QSP model

Eq (2) is also implemented in ABM for inverse scaling such that the number of cells in the tumor compartment of the QSP model is estimated from the number of cells in ABM every time step since ABM gets initialized. This condition is applied to cancer cells, the sum of effector and cytotoxic T cells, exhausted/suppressed T cells, regulatory T cells, and MDSCs such that

$$C=\gamma \left(S_{t}+\sum_{i=1}^{d_{max}}P_{i}+S_{e}\right),T_{1}=\gamma (T_{eff}\prime +T_{cyt}\prime ),$$

$$T_{exh}=\gamma T_{exh}\prime ,T_{0}=\gamma T_{0}\prime ,MDSC=\gamma MDSC\prime .$$ 7

where *C* is the total number of cancer cells in the QSP model, *S*_*t*_, *P*_*i*_, and *S*_*e*_ refer to the number of cancer stem-like cells, progenitor cells after *i* divisions, and senescent cells in ABM, respectively, and *T*_*cyt*_′ is the number of cytotoxic T cells in ABM.

Section A.4 of the S1 Supplementary Material includes comparisons between results obtained with the QSP model and with spQSP model for different cases. The outcomes are qualitatively equivalent but not exactly the same due to the stochastic effects and the explicit description of cell subtypes of ABM. This comparison shows that the proposed QSP-ABM coupling guarantees the self-consistency of the hybrid spQSP model. Also, the continuous feedback between ABM and QSP provides information about the influence of stochasticity generated in ABM in the species represented in the QSP model.

## Results

All QSP model and ABM parameter values used in the simulations performed for this study are listed in S1 and S2 Tables. Although, the spatial QSP model calculates local cancer cell densities for the specific goal of defining T cell and MDSC recruitment probabilities (Section A.2 of the S1 Supplementary Material), cell densities are not among the outputs of the model. Thus, for graphical representation purposes, we have used the open-source software environment R where density calculations, based on the spatial location of cells, are implemented in the R functions *stat_density_2d* and *scale_fill_gradient*. The former performs a 2D kernel density estimation [53] and displays the results with contours (https://ggplot2.tidyverse.org/reference/geom_density_2d.html), the latter creates a two color gradient from low to high density (https://ggplot2.tidyverse.org/reference/scale_gradient.html). 3D representations of the tumor have been rendered with the open-source software Blender.

### Spatio-temporal evolution of the tumor

The tumor growth dynamics is determined by the balance between the migration and proliferation rates of cancer cells. By defining a set of non-dimensional parameters, we can describe different tumor shapes and sizes over time. Although, the tumor growth is driven by the proliferation rates of CSCs and PCs, *r*_*st*_ and *r*_*p*_, respectively, we should also take into account the rate at which they migrate through the tumor microenvironment, represented by their migration rates, *u*_*st*_ and *u*_*p*_. Thus, we express the first non-dimensional parameter *R* as follows

$$R=\frac{r_{st}u_{p}}{r_{p}u_{st}}.$$ 8

The other non-dimensional parameters are the probability of division, *k*, and the maximum number of progenitor cell divisions, *d*_*max*._

**Fig 3:**
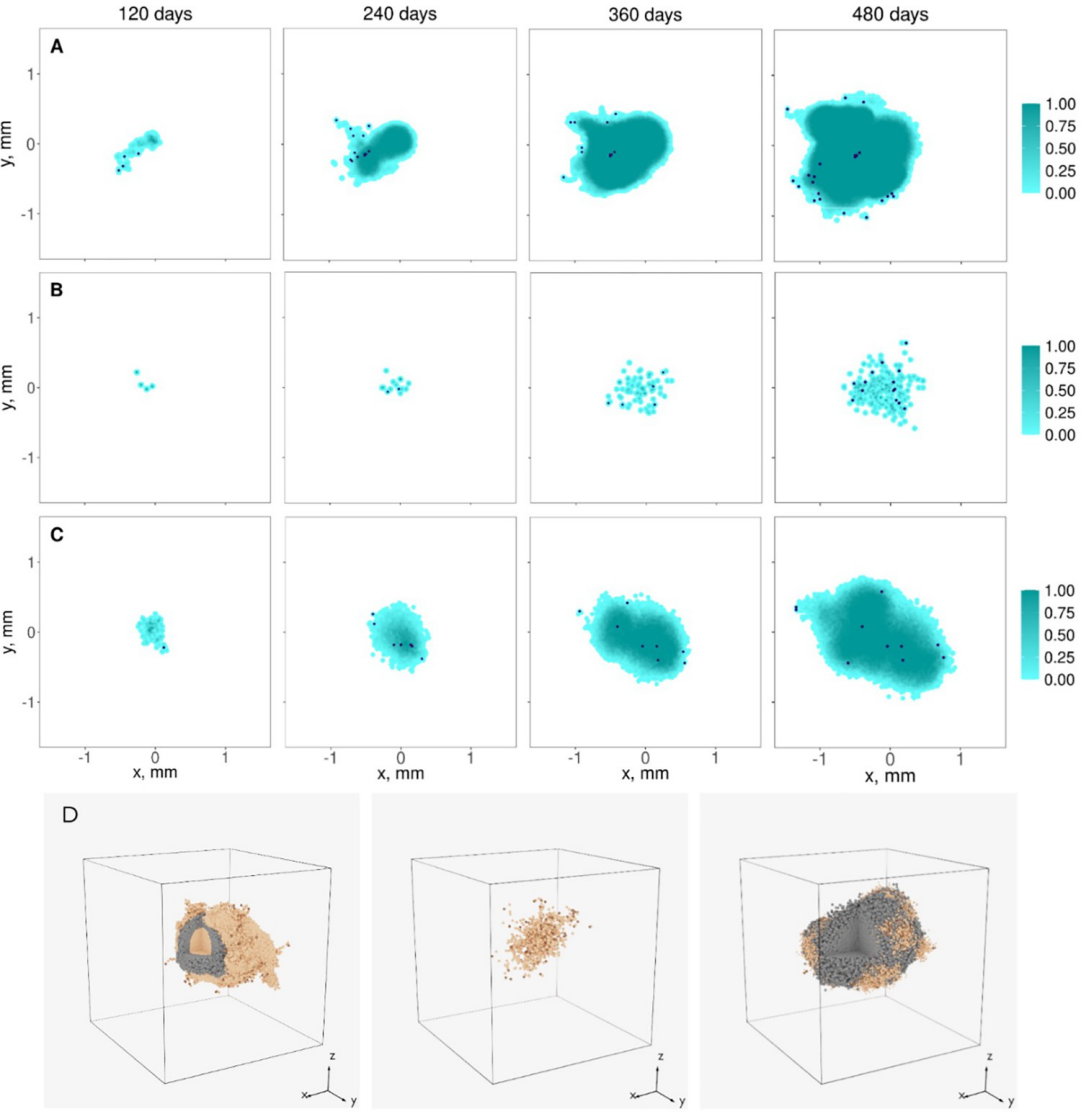
Scaled spatio-temporal evolution of the tumor. The relation between CSC and PC migration and proliferation rates, *R*, the asymmetric division probability, *k*, and the maximum number of progenitor cell divisions, *d*_*max*_, define the evolution in time of the tumor shape and size. In panels A-C, the turquoise scale bar represents the normalized cancer cell density of slices at the center of the tumors with 0.2 mm thickness every 4 months; the dark blue dots are CSCs. In panel D, dark brown, light brown, and grey dots represent CSCs, PCs, and senescent cells, respectively. Thus, three scenarios are presented: *R <* 1, high probability of asymmetric division, *k* = 0.95, and a large maximum number of progenitor cell divisions, *d*_*max*_ = 18 (panel A); *R ~* 1, low probability of asymmetric division, *k* = 0.75, and *d*_*max*_ = 18 (panel B); *R ~* 1, *k* = 0.95, and a small maximum number of progenitor cell divisions, *d*_*max*_ = 9 (panel C). The spatial QSP algorithm calculated the evolution of three-dimensional tumors for 16 months starting from one CSC located at the center of the grid. Panel D shows the three-dimensional spatial representation of the tumors from panels A-C after 16 months of growth. All simulations were performed in a 3x3x3 mm grid. Cases with spherical shapes are included in section B.1 of the S1 Supplementary Material. The scaling factor is *γ*=1 in all cases.

For the study of the spatio-temporal evolution of tumors, we have set the recruitment of T cells and MDSCs to zero, assume the presence of one stem-like cancer cell in the center of the ABM grid at the beginning of the simulation, and the scaling factor *γ* equals to 1 in all simulations. From our analysis in Table 1 we conclude that the combination of high proliferation rates of CSCs and high migration rates of PCs, i.e., *R >* 1, a high probability of asymmetric division, and a large maximum number of progenitor cell divisions lead to large, spherical, and significantly dense tumors (S4A Fig and first figure of S4C Fig). The absence of CSCs at the IF of the tumor ensures isotropic growth. When *R ~* 1, the isotropic growth still takes place, although the tumor gets smaller and denser (S4B Fig and second figure of S4C Fig). On the contrary, when CSC proliferation rates and PC migration rates are low, i.e., *R <* 1, and the probability of asymmetric division is high, the tumor becomes anisotropic. We observe that some groups of cells get detached from the primary tumor or form fingering structures (Fig 3A and first figure of Fig 3D). For cases with *R ~* 1 and low probability of asymmetric division the number of PCs gets reduced, and the tumor cells follow a sparse distribution (Fig 3B and second panel of Fig 3D). Finally, the maximum number of progenitor cell divisions defines the density of the tumor and the number of senescent cells (Fig 3C and third figure of Fig 3D). The higher *d*_*max*_, the denser the tumor and the lesser the number of senescent cells.

**Table 1:** Spatio-temporal characterization of tumors in terms of the non-dimensional cancer cell parameters *R*, *k*, and *d*_*max*_. Cases A-C correspond to panels A-C, respectively, in Fig 3. CSCs refers to cancer stem-like cells.

CASE	A	B	C	A (S4 Fig)	B (S4 Fig)
***R***	< 1	1	1	> 1	1
***k***	High	Low	High	High	High
***d***_***max***_	Large	Large	Small	Large	Large
**Growth rate**	Medium	Slow	Medium	Fast	Medium
**Size**	Medium	Small	Medium	Big	Medium
**Shape**	Non-regular	Non-regular	Non-regular	Regular	Regular
**Density**	High	Low	Medium	Medium	High
**CSCs, location**	Spread	Spread	Spread	Centered	Off-centered
**CSCs, number**	High	Medium	Medium	Very low	Low

### Spatio-temporal characterization of the immune response

In this section we present spatial and temporal comparisons of the immune response under different tumor growth conditions without and with immunotherapy. For this analysis, ABM is used as a coarse-grained model to represent the entire tumor. Some examples of coarse-grained approaches where an agent represents groups of cancer cells can be found in [54,55,56]. In our model, the representation of large groups of cells as agents introduces randomness at a mesoscale that a macroscopic model would not capture, and the macroscopic temporal dynamics of the tumor is ensured by the consistent coupling between the QSP model and ABM. Thus, the scaling factor *γ* that we choose for the following simulations is equivalent to the number of cells represented by each agent. In this analysis, we assume that *γ* = 5x10^4^ for all simulations.

### Spatial distribution of cell densities without immunotherapy

**Fig 4:**
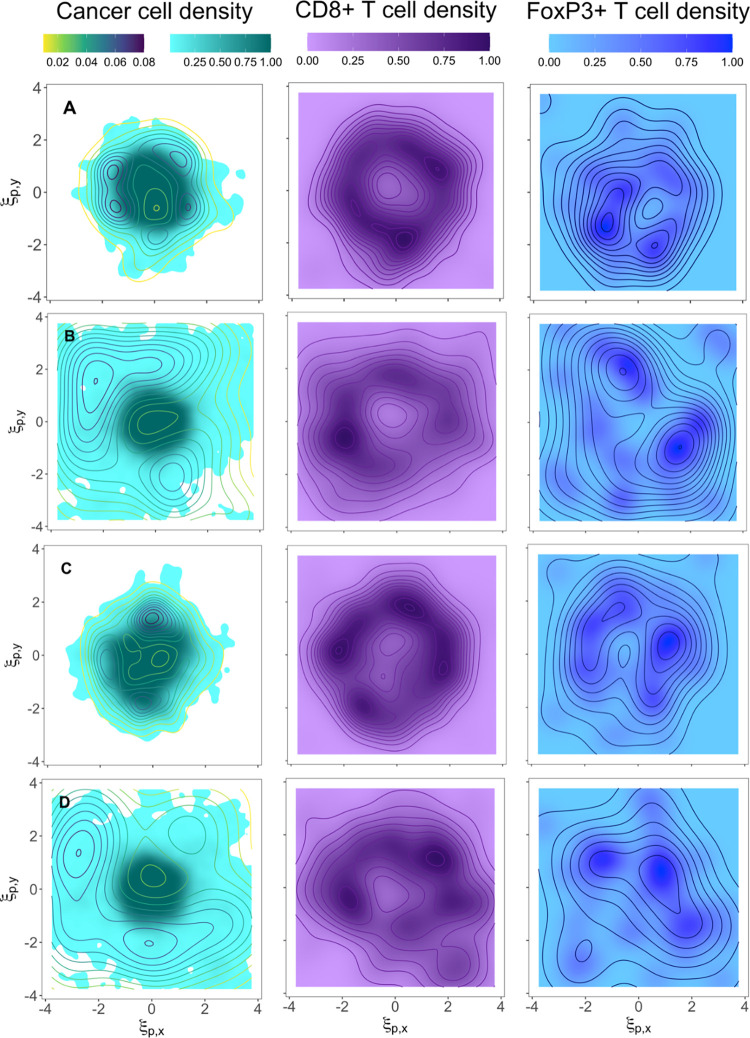
Spatial distributions of cell densities under different tumor growth conditions. Cancer cell, CD8+ T cell, and FoxP3+ T cell densities are normalized and represented in turquoise, purple, and blue scale bars, respectively. CSC density distributions are represented in the first column of contour plots as yellow-to-blue lines. ξ_p,y_ = y/*y*_*ref*_ and ξ_p,x_ = x/*x*_*ref*_ are non-dimensional spatial coordinates with *y*_*ref*_ = *x*_*ref*_ = 2u_p_/r_p_. Four different scenarios are presented: similar migration and proliferation effects, *R* ~ 1, 10% of the grid occupied by voxels with cell recruitment sources, and *d*_*max*_ = 9 (panel A); fast migration of CSCs and proliferation of PCs, R < 1, 10% of the grid occupied by voxels with cell recruitment sources, and *d*_*max*_ = 9 (panel B); *R* ~ 1, 10% of the grid occupied by voxels with cell recruitment sources, and *d*_*max*_ = 4 (panel C); R < 1, 30% of the grid occupied by voxels with cell recruitment sources, and *d*_*max*_ = 9 (panel D). The spatial QSP algorithm calculated the evolution of three-dimensional tumors starting from a normal distribution of cancer cells located at the center of the grid. QSP model and ABM are coupled before reaching the point where T cells are recruited and also before the initial tumor diameter condition from the QSP model is met. Thus, no initial T cell spatial distribution is enforced. The figures show the cancer cell density in a two-dimensional slice at the center of the tumor 10 months after the initial tumor diameter condition is met. The scaling factor is *γ*=50000 in all cases.

In order to study the spatial distribution of cells in the tumor microenvironment, we have assigned Gaussian kernel density estimates to the agents to plot cell densities instead of individual entities. Thus, regions with high cell density represent the locations where it is more likely to find cells and vice versa. In digital pathology studies these regions are known as cancer or immune hotspots [57,58,59,60]. The coarse-grained application of the spatial QSP combines the characteristics of two methods that are used for CD8+ T cell enumeration in prognosticating TNBC: hotspot versus whole-tumor [61,62]. It provides information at the whole-tumor scale while estimating the regions where the main hotspots are located. Table 2 summarizes the main features of cancer cell, CD8+ T cell, and FoxP3+ T cell density spatial distributions presented in Fig 4, where all contour plots represent cell densities ten months after the initial diameter condition is met. CD8+ T cells and FoxP3+ T cells are assumed to get recruited everywhere but with higher probability at the IF of the tumor.

**Table 2:** Spatial characterization of the immune response under different tumor growth conditions. R.V. stands for recruitment volume and refers to the percentage of spatial domain occupied by voxels with cell recruitment points (where only one recruitment point is assumed per voxel); CSCs denotes cancer stem-like cells.

	Parameters			Cell spatial distributions		
Panel	*R*	*d*_*max*_	R.V. (%)	Tumor	CD8+ T cells	FoxP3+ T cells
A	~ 1	Large	10	**Regular** shaped core, **narrow** region of low density at the IF. CSCs: present all over the tumor.	**Uniform** and **high** density around tumor, **narrow** region	**Partially** defined **circular** pattern
B	< 1	Large	10	**Regular** shaped core, **wide** region of low density at the IF. CSCs: High and low gradients, widely spread	**Non-uniform** and **low** density around tumor, **wide** region	**No clear pattern**
C	~ 1	Small	10	**Non-regular** core, **narrow** region of low density at the IF. CSCs: present all over the tumor.	**Uniform** and **high** density around tumor, **narrow** region	**Partially** defined **circular** pattern
D	< 1	Large	30	**Regular** shaped core, **wide** region of low density at the IF. CSCs: High and low gradients, widely spread	**Non-uniform** and high density around tumor, **wide** region	**No clear pattern**

Our analysis shows that the variation of some dimensionless parameters can be associated with specific spatial patterns in the tumor and, consequently, in the T cell distributions. For instance, the relation between migration and proliferation rates, *R*, is critical in the shape of the tumor and the location of low cancer cell density regions. For *R ~* 1, the tumors stay quite circumscribed and quasi-rounded (Fig 4A and 4C) which is consistent with the observations from radiological and ultrasound images [63,64,65]. Nevertheless, although CSC densities in both panels A and C get confined in the tumor region (multicolor lines in cancer cell contour plots of Fig 4), only the cell density of CSCs in panel C gets completely circumscribed on the body of the tumor. For *R <* 1, the tumor shape becomes very irregular, some fingering structures form at the IF, and there exists a wide diffuse region of low cancer cell density surrounding the core of the tumor (Fig 4B and 4D) [63,64,65]. Panel C shows that a small maximum number of progenitor cell divisions introduces anisotropic growth in the core of the tumor (dark turquoise region) when compared to panel A. We also see that a larger recruitment volume does not seem to affect the cancer cell density distribution in the tumor when comparing panels B and D.

CD8+ T cell density in panels A and C form a uniform region around the core of the tumor. Densities are practically zero in regions far from the core. CD8+ T cell density plots, however, show that the widely spread distribution of cancer cells in panel B and D has a strong influence on generating non-uniform T cell distributions. Low CD8+ T cell density in the core and a combination of low and high density (hotspots) regions in the surroundings of the core, as panels B and D show, have been experimentally observed in breast cancer [66]. From a digital pathology perspective, the relevance of the location of hotspots has been reported since the amount of co-localized cancer and immune hotspots correlates with a good prognosis in breast cancer [59,61]. Similar to cancer cells, CD8+ T cells are more spread out as the parameter *R* decreases.

FoxP3+ T cell hotspots are highly dense, but their numbers are low. They are mostly located at the periphery of the tumor, and only in cases with *R ~* 1 (i.e., panels A and C), they form a quasi-circular pattern around the core of the tumor.

### Spatial distribution of cell densities with immunotherapy

The results above illustrated tumor growth without pharmacological interventions. Now we will present results with anti-PD-1 immunotherapy, assuming 3 mg/kg nivolumab is administered as a single agent every two weeks, to partially mimic the previous simulations with the non-spatial QSP model in [46]. It should be noted that the purpose of the present study is primarily methodological, to formulate spQSP model and provide exemplary simulations, rather than reproduce conditions of a specific clinical trial.

**Fig 5:**
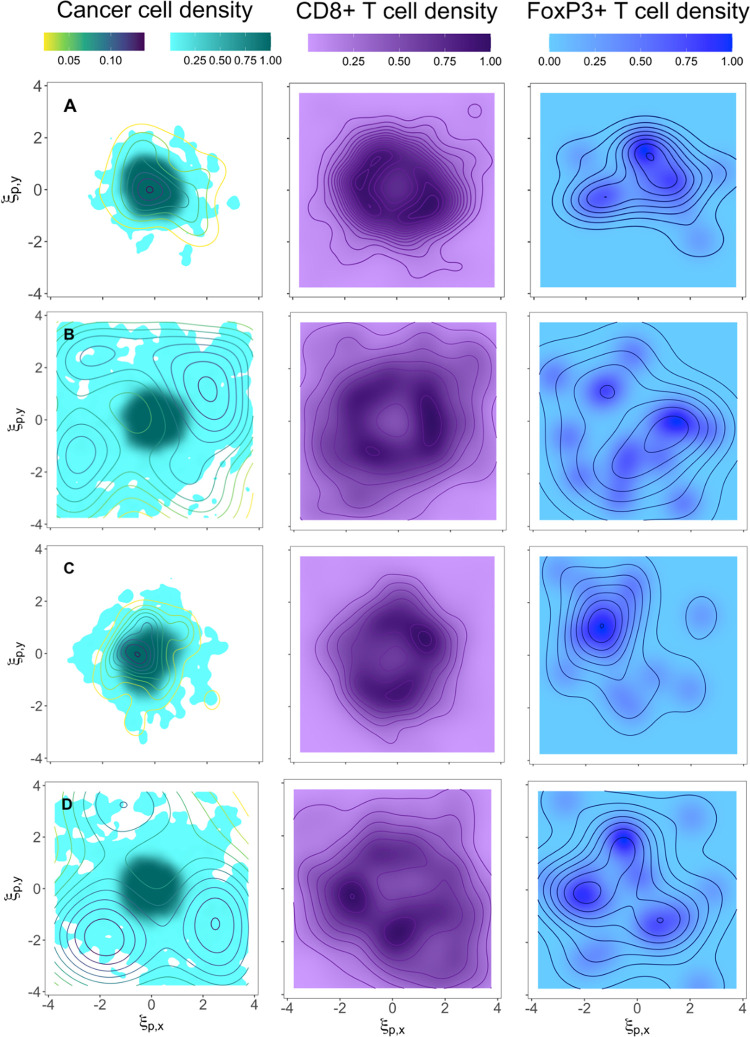
Spatial distributions of cell densities under different tumor growth conditions and immunotherapy. Cancer cell, CD8+ T cell, and FoxP3+ T cell densities are normalized and represented in turquoise, purple, and blue scale bars, respectively. CSC density distributions are represented in the first column of contour plots as yellow-to-blue lines. ξ_p,y_ = y/*y*_*ref*_ and ξ_p,x_ = x/*x*_*ref*_ are non-dimensional spatial coordinates with *y*_*ref*_ = *x*_*ref*_ = 2u_p_/r_p_. Four different scenarios are presented: similar migration and proliferation effects, *R* ~ 1, 10% of the grid occupied by voxels with cell recruitment sources, and *d*_*max*_ = 9 (panel A); fast migration of CSCs and proliferation of PCs, R < 1, 10% of the grid occupied by voxels with cell recruitment sources, and *d*_*max*_ = 9 (panel B); *R* ~ 1, 10% of the grid occupied by voxels with cell recruitment sources, and *d*_*max*_ = 4 (panel C); R < 1, 30% of the grid occupied by voxels with cell recruitment sources, and *d*_*max*_ = 9 (panel D). The spatial QSP algorithm calculated the evolution of three-dimensional tumors starting from a normal distribution of cancer cells located at the center of the grid. QSP model and ABM are coupled before reaching the point where T cells are recruited and also before the initial tumor diameter condition from the QSP model is met. Thus, no initial T cell spatial distribution is enforced. The figures show the cancer cell density of a two-dimensional slice at the center of the tumor 10 months after the initial tumor diameter condition is met and 3 mg/kg nivolumab is administered every two weeks. The scaling factor is *γ*=50000 in all cases.

Table 3 summarizes the main features of cancer cell, CD8+ T cell, and FoxP3+ T cell density spatial distributions presented in Fig 5, where all contour plots represent cell densities ten months after the initial diameter condition is met and 3 mg/kg nivolumab is administered every two weeks.

**Table 3:** Spatial characterization of the immune response under different tumor growth conditions and immunotherapy. R.V. stands for recruitment volume as defined in Table 2; CSCs denotes cancer stem-like cells.

	Parameters			Cell spatial distributions		
Panel	*R*	*d*_*max*_	R.V. (%)	Tumor	CD8+ T cells	FoxP3+ T cells
A	~ 1	Large	10	**Regular** shaped core, **finger structures** and **clusters** at the IF. CSCs: present all over the tumor.	**Uniform** and **high** density around tumor, **narrow** region	**No pattern, a few hotspots**
B	< 1	Large	10	**Regular** shaped core, **finger structures** at the IF. CSCs: Low gradients, widely spread	**Non-uniform** density around tumor, **wide** region	**No pattern, several hotspots**
C	~ 1	Small	10	**Non-regular** shaped core and **finger structures** at the IF. CSCs: mostly present in the body of the tumor	**Uniform** and **high** density around tumor, **narrow** region	**No pattern, a few hotspots**
D	< 1	Large	30	**Regular** shaped core, **finger structures** and **clusters** at the IF. CSCs: Low gradients, widely spread	**Non-uniform** density around tumor, **wide** region	**No pattern, several hotspots**

Regarding cancer cell densities, we observe that the sizes of the cores have decreased after one year of treatment, although their shapes remain quite similar to the cases without treatment. The boundaries, however, have significantly changed in panels A and C since tumors are now surrounded by cancer progenitor cell (PC) fingering structures and clusters. Again, although CSC densities in both panels A and C are mostly confined in the tumor region, only the cell density of CSCs in panel C still seems completely confined in the body of the tumor. The boundaries in panels B and D have been partially removed and the result is a combination of clusters of cancer cells and fingering structures. The gradients of CSC densities are mostly low in panels B and D when immunotherapy is applied. From a clinical perspective, one of the most interesting observations is the lack of CSC density regions in the majority of clusters in panel A and in the fingering structures of panel C, whereas panels B and D have CSC density presence in the majority of the clusters and fingering structures, an indication of their invasive behavior and potential transition to a metastatic stage.

Immunotherapy significantly changes CD8+ T cell density distribution in panel A, but it improves the immune response in panel C even more. CD8+ T cell density gets confined in a small region around the core of the tumor. In contrast, panels B and D show widely spread CD8+ T distributions with immunotherapy. The immune cells are more spread out and located in hotspots at the IF of the tumors after ten months of treatment.

FoxP3+ T cell hotspots are less dense now and their numbers are still low. They are mostly located at the periphery of the tumor and do not form any spatial pattern.

### Temporal evolution of cells without and with immunotherapy

**Fig 6:**
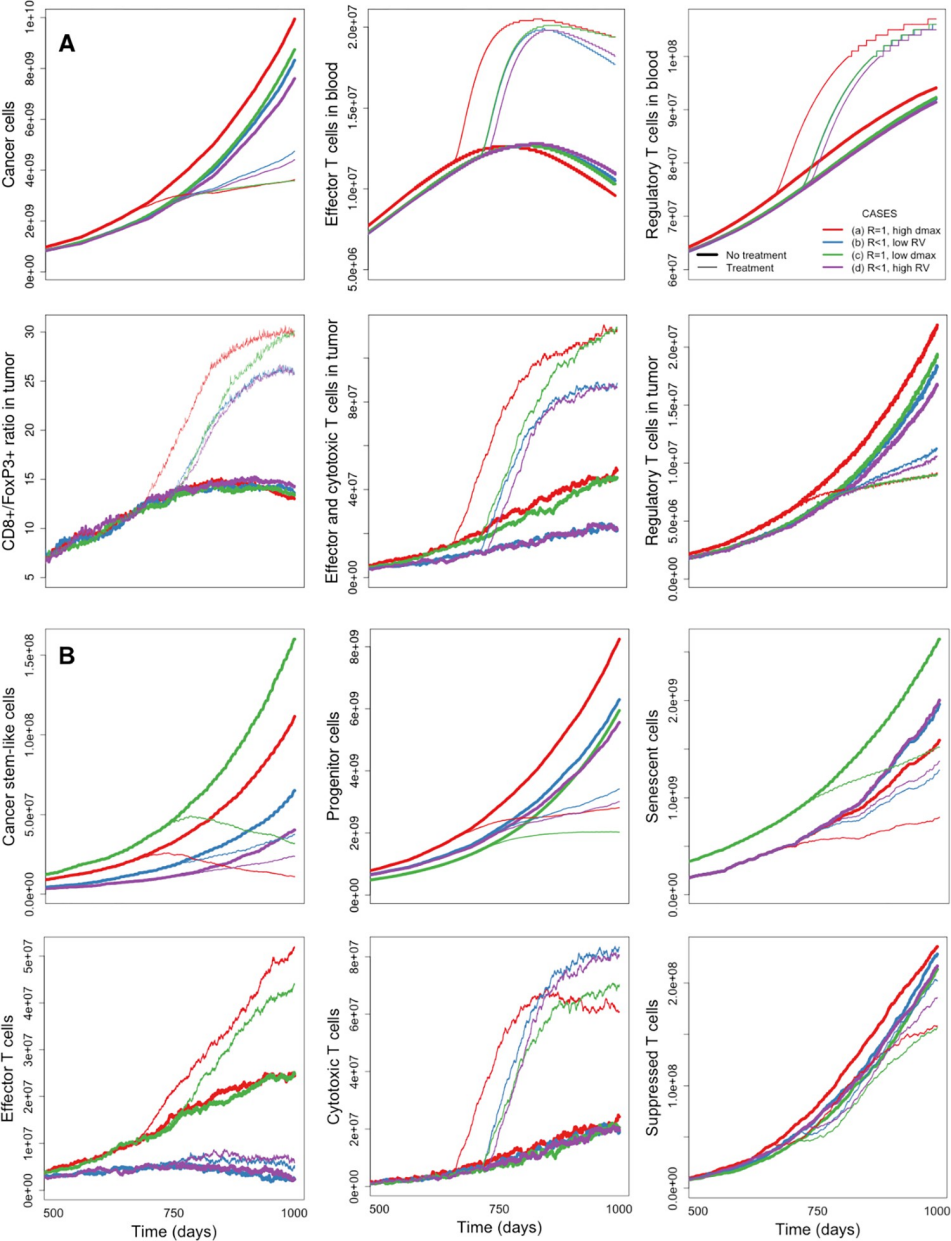
Temporal evolution of cells in the tumor microenvironment under different tumor growth conditions without and with immunotherapy. Panel A: variation in time of number of cancer cells, T cells in central and tumor compartments, and CD8+/FoxP3+ ratio. Panel B: variation in time of cancer cell and CD8+ T cell subtypes. Thick and thin red lines represent cases (a) and (a*) without and with immunotherapy, respectively (*R ~* 1, 10% of the grid occupied by voxels with cell recruitment sources, and *d*_*max*_ = 9); thick and thin blue lines represent cases (b) and (b*) without and with immunotherapy, respectively (*R <* 1, 10% of the grid occupied by voxels with cell recruitment sources, and *d*_*max*_ = 9); thick and thin green lines represent cases (c) and (c*) without and with immunotherapy, respectively (*R ~* 1, 10% grid occupied by voxels with cell recruitment sources, and *d*_*max*_ = 4); thick and thin purple lines represent cases (d) and (d*) without and with immunotherapy, respectively (*R <* 1, 30% of the grid occupied by voxels with cell recruitment sources, and *d*_*max*_ = 9).

Fig 6 shows the evolution in time of different cell types and subtypes for cases presented in Fig 4 (without immunotherapy) and Fig 5 (with immunotherapy). In order to avoid confusion, we assign cases (a)-(d) to Fig 4A–4D, and cases (a*)-(d*) to the panels Fig 5A–5D. Thick and thin lines represent cases (a)-(d) and (a*)-(d*) in Fig 6, respectively. We analyze the differences as follows.

In Fig 6, panel A, the most efficient immune response takes place in case (d) (thick purple line) when it is compared to the other cases. By looking at the temporal outcome, we would conclude that case (d) is the tumor that grows smaller, however, our spatial analysis showed a very invasive tumor with non-regular spatial distribution. Case (b) (thick blue line) is quite similar, but the percentage of grid occupied by voxels with cell recruitment sources is lower and the tumor has a larger number of cancer cells. Cases (a) and (c) (thick red and green lines, respectively) have more cancer cells, but the number of effector and cytotoxic T cells as well as regulatory T cells in the tumor are higher. Despite of having more cancer cells, these tumors have regular shape and small size. These are very interesting observations since the QSP model only provides temporal outcomes and this analysis shows that QSP outcomes could be deceitful in the absence of spatial simulations.

When immunotherapy is applied, we see effective responses in cases (a*) and (c*) (thin red and green lines, respectively), despite having apparent ineffective responses without treatment. Both cases have similar number of cancer cells, number of T cells in the blood compartment, CD8+ T cells to FoxP3+ T cells ratio, and T cells in the tumor at the end of the treatment. Cases (b*) and (d*) (thin blue and purple lines, respectively) are not that responsive to immunotherapy and their evolution in time are quite similar to each other. Case (d*) responds slightly better than case (b*) in terms of cancer cells in the tumor.

Fig 6B shows again that cases (b) and (d) are apparently the most optimal scenarios since the number of CSCs, PCs, and effector T cells increase at a much slower rate than in cases (a) and (c). Again, this does not reflect the fact that tumors in cases (b) and (d) are highly invasive. Cases (a) and (c) have many more CSCs, but they are confined in the body of the tumor. The invasiveness of these tumors also gets inhibited by a higher number of effector T cells that mostly get recruited at the IF.

Cell subtypes analysis confirms that immunotherapy strongly improves the immune response of cases (a) and (c). The number of CSCs decreases immediately after treatment is applied. They stay confined in the tumor and do not have much space to proliferate. Thus, fewer PCs are generated, and the immune response efficiently transforms them into SCs. In cases (b) and (d) the number of cytotoxic T cells significantly increases, but it is not enough for an effective response to immunotherapy.

### Definition and characterization of the invasive front

We use the spQSP model to describe the characteristics of the IF of the tumor. Since ABM considers discrete cells that do not form a continuum, it is necessary to define IF that is consistent with pathologist’s definition. Thus, for the sake of accuracy, graphical representations of the IF at the ROI scale require some additional mathematical analysis. We present below an analytical approach that defines the smoothness and boundaries of a kernel density function based on tumor growth properties and the IF pathologist’s definition. Smoothness and boundaries are introduced as inputs in the R functions *stat_density_2d* and *scale_fill_gradient* in the form of kernel density standard deviation and minimum/maximum normalized cancer cell densities, respectively.

**Fig 7:**
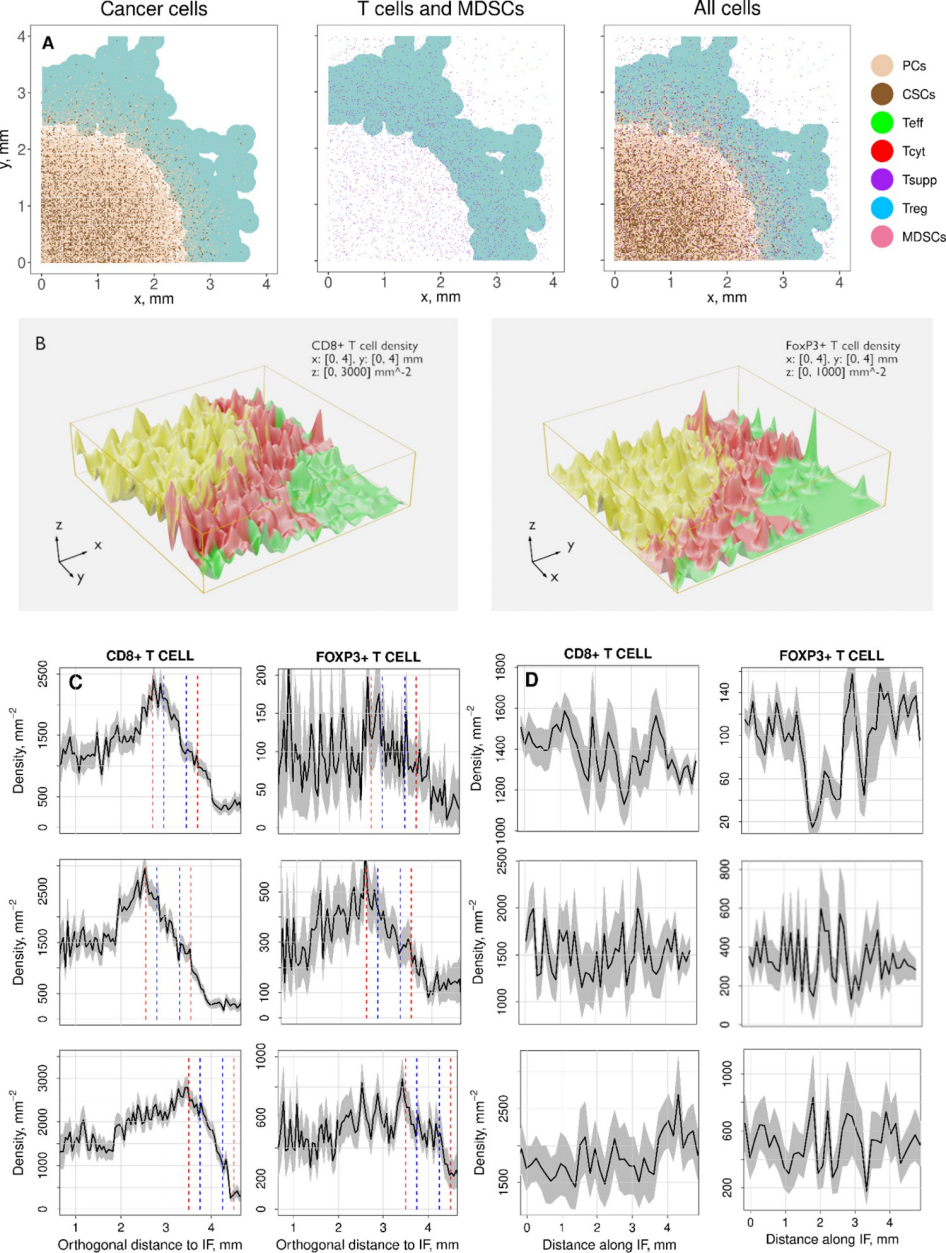
Cell distribution and cell density at the IF. Panel A: Spatial representation of cancer cell subtypes (left), CD8+ T cells subtypes, FoxP3+ T cells, and MDSCs (center), and all cells (right) in a section of a tumor slice. The IF region is depicted in pale turquoise. Slow tumor growth case (*k*_C1,growth_ = 0.005 day^-1^) and R << 1 (R = 1/50). Panel B: 3D representation of CD8+ T cell and FoxP3+ T cell densities in the central tumor (CT; yellow), the invasive front (IF; red), and the normal tissue (N; green). Panel C: CD8+ T cell and FoxP3+ T cell density profiles along the direction perpendicular to the IF averaged over the circumference of the IF. 95% confidence intervals are calculated (grey areas). Two definitions of IF are introduced here and are indicated as vertical lines, blue: width w_pathol_=0.5 mm; red: width w_pathol_=1 mm. Panel D: CD8+ T cell and FoxP3+ T cell density profiles along the direction perpendicular to the IF averaged over the circumference of the IF. In panels C and D every row is a case with a different combination of ratios *k*_C1,growth_*/k*_C,T1_, *k*_T1_*/k*_Treg_, and the parameter *T*_reg,max_, where *k*_C1,growth_, *k*_C,T1_, *k*_T1_, *k*_Treg_, and *T*_reg,max_ are the cancer cell growth rate, the rate of cancer cell death by T cells, the exhaustion rate of cytotoxic T cells by all cells that express PD-L1, the inhibition rate of cytotoxic T cells by regulatory T cells, and the maximal regulatory T cell density in the tumor, respectively. The spatial QSP algorithm calculated the evolution of a tumor slice starting from a fraction of a normal distribution of cancer cells. QSP model and ABM are coupled before reaching the point where T cells are recruited and also before the initial tumor diameter condition from the QSP model is met. Thus, no initial T cell spatial distribution is enforced. The figures here show cell distributions and densities 6 months after the initial tumor diameter condition is met.

**Fig 8:**
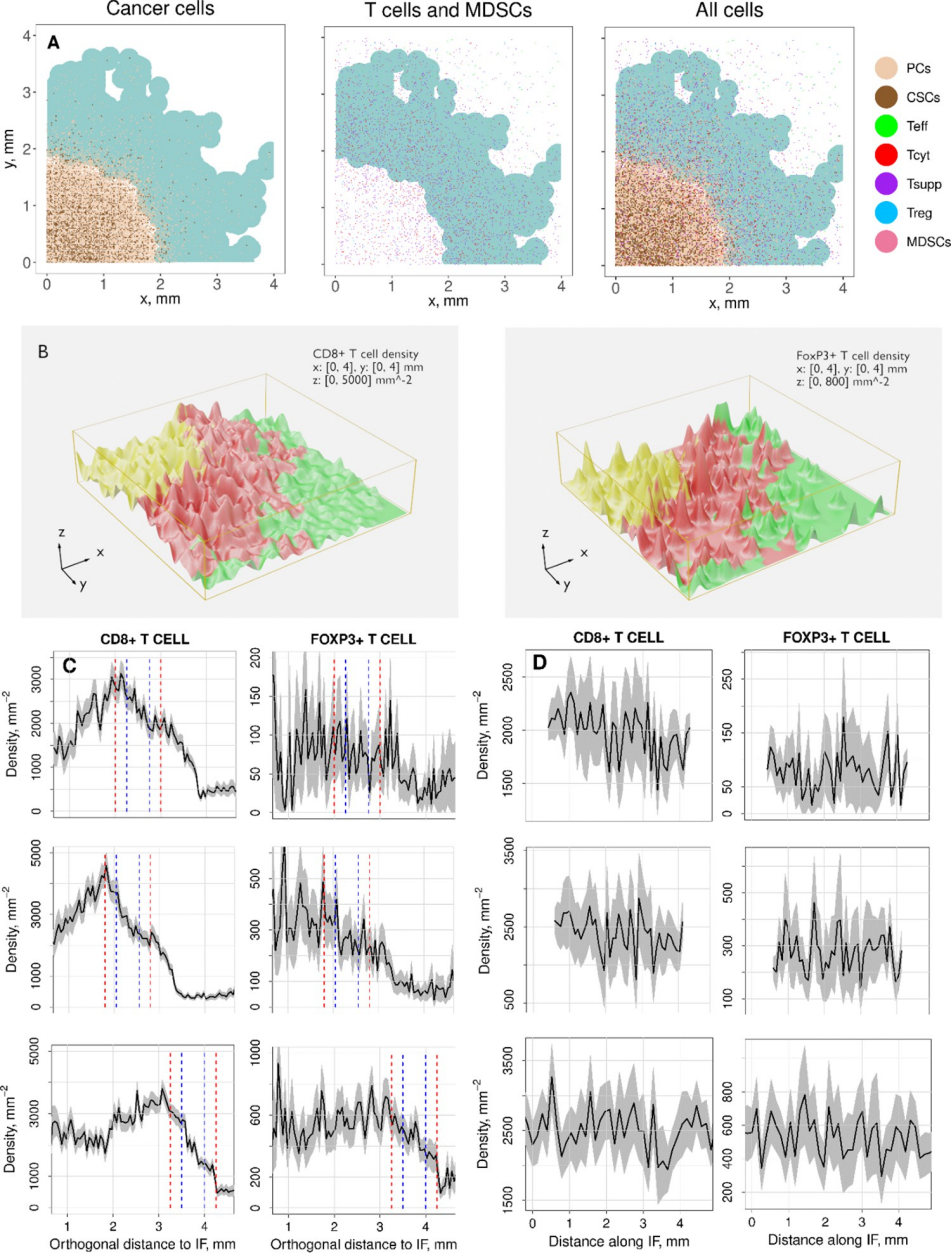
Cell distribution and cell density at the IF under treatment. Panel A: Spatial representation of cancer cell subtypes (left), CD8+ T cells subtypes, FoxP3+ T cells, and MDSCs (center), and all cells (right) in a section of a tumor slice. The IF region is depicted in pale turquoise. Slow tumor growth case (*k*_C1,growth_ = 0.005 day^-1^) and R << 1 (R = 1/50). Panel B: 3D representation of CD8+ T cell and FoxP3+ T cell densities in the central tumor (CT; yellow), the invasive front (IF; red), and the normal tissue (N; green). Panel C: Average of CD8+ T cell and FoxP3+ T cell density profiles that are perpendicular to the IF. 95% confidence intervals are calculated upon the profile (grey areas). Two definitions of IF are introduced here and are indicated as vertical lines, blue: width w_pathol_=0.5 mm; red: width w_pathol_=1 mm. Panel D: Average of CD8+ T cell and FoxP3+ T cell density profiles along the IF. In panels C and D every row is a case with a different combination of ratios *k*_C1,growth_*/k*_C,T1_, *k*_T1_*/k*_Treg_, and the parameter *T*_reg,max_, where *k*_C1,growth_, *k*_C,T1_, *k*_T1_, *k*_Treg_, and *T*_reg,max_ are the cancer cell growth rate, the rate of cancer cell death by T cells, the exhaustion rate of cytotoxic T cells by all cells that express PD-L1, the inhibition rate of cytotoxic T cells by regulatory T cells, and the maximal regulatory T cell density in the tumor, respectively. The spatial QSP algorithm calculated the evolution of a tumor slice starting from a fraction of a normal distribution of cancer cells. QSP model and ABM are coupled before reaching the point where T cells are recruited and also before the initial tumor diameter condition from the QSP model is met. Thus, no initial T cell spatial distribution is enforced. The figures here presented show cell distributions and densities 6 months after the initial tumor diameter condition is met with 3 mg/kg nivolumab administered every two weeks.

Examples of IF regions are shown in pale turquoise color in panel A in Fig 7 (without immunotherapy) and Fig 8 (with immunotherapy). Here, we explain the definition of the IF. To create a continuous outer boundary of the IF, it is necessary to introduce an averaging or smoothing procedure to transition from the discrete cells. We choose to use a 2D Gaussian kernel density [53] in the form

$$G(\vec{x},\sigma )=\frac{1}{2\pi \sigma^{2}}e^{-\frac{{\left|\vec{x}\right|}^{2}}{2\sigma^{2}}},$$ 9

where $\left|\vec{x}\right|$ is approximately half of the maximum observable distance between cells that are far from the core and *σ* is the standard deviation. It is a 2D kernel because tumor slices are projected onto 2D planes in panel A in Figs 7 and 8 and S5 and S6. A cutoff cancer cell density is estimated from the analysis performed in [67] as $\rho_{cutoff}^{min}∼\varepsilon \;with\;\varepsilon =C_{min}/C$ where *C*_*min*_ is the cell number cutoff and *C* is the total number of cancer cells in the tumor. $\rho_{cutoff}^{min}$ is the value of the function below which the cell presence is considered zero, therefore, it defines the outer boundary of the IF. Normalizing the cutoff density, we express a cutoff Gaussian kernel density as Gcutoff=ρcutoffmin(2πσ2), and obtain an analytical estimate of the standard deviation *σ* as follows

$$\rho_{cutoff}^{min}={2\pi \sigma^{2}G}_{cutoff}\to \varepsilon ∼e^{-\frac{{\left|\vec{x}\right|}^{2}}{2\sigma^{2}}}\to \sigma ∼\frac{\left|\vec{x}\right|}{\sqrt{2\ln\left(\frac{1}{\varepsilon }\right)}}.$$ 10

A more heuristic way to define the kernel function without estimating $\left|\vec{x}\right|$ is to try different values for *σ*, from large to small, until the pale turquoise areas surrounding the cells are as small as possible but still forming a continuous region altogether. Note that according to both analytical and heuristic methods: 1) we need the value of the estimated cutoff density and 2) we minimize the number of individual cancer cells and cell aggregates that may form islands outside the outer boundaries.

To define the inner boundary of the IF, we note that, on average, cancer cell density increases from zero at the outer boundary to values that reach a plateau towards the tumor core. Normalizing the density such that the dimensionless density *ρ* varies between 0 and 1, we introduce a cutoff value, $\rho_{cutoff}^{max}$, which then defines the inner boundary of the IF. In general, this value could be chosen so that the width of the IF would correspond to that conventionally used in the field cancer pathology, *w*_pathol_, typically 0.5mm or 1mm [28,68,69,70,71]. In defining $\rho_{cutoff}^{max}$ we have used theoretical analysis of wave front propagation governed by the partial differential equation S48, from Section A.3 of the S1 Supplementary Material, that describes diffusion and proliferation of cancer cells. Section A.3 also describes the definition of the IF based on local cancer cell densities and an analytical solution of the deterministic Fisher-Kolmogorov-Petrovsky-Piskunov equation, used in the studies of population growth and wave propagation [56,72]. Then, the value of $\rho_{cutoff}^{max}$ is equivalent to the normalized cancer cell density at the inner boundary of the 1 mm wide IF, *ρ*_*max*,*IF*_, expressed analytically in terms of *w*_pathol_ by equation S56. Even though the boundaries of the IF are irregular and stochastic, the width of the IF (the pale turquoise region) is approximately equal to *w*_pathol_.

### Cell distribution at the IF and T cell infiltration profiles without immunotherapy

Fig 7A presents the spatial distribution of all subtypes of cancer cells, CD8+ T cells, FoxP3+ T cells, and MDSCs in a section of a tumor slice 6 months after the initial diameter condition is met. PCs (light brown) compose most of the tumor domain, however, there is also a significant number of CSCs (dark brown) distributed over the tumor. Since the migration rate of CSC is dominant, the edge of the tumor is not smooth, and some CSCs and PCs randomly escape from it. Senescent cells are absent in this particular case. CD8+ T cells, FoxP3+ T cells, and MDSCs are assumed to get recruited everywhere, but with a higher probability at the IF of the tumor. CD8+ T effector cells (green) are mostly located outside or around the outer boundary of the IF; cytotoxic cells (red), and suppressed or exhausted cells (purple), can be mainly found at the inner boundary of the IF and throughout the tumor. The number of FoxP3+ T cells (blue) is lower than CD8+ T cells but their spatial distributions are quite similar. Finally, a few MDSCs (pink) are recruited but their influence is comparatively small.

Following the above definition of the IF, Fig 7A depicts it as a region of approximately 1 mm wide with some fingering structures emerging at the edge (pale turquoise). A significant number of cancer cells are located at the inner boundary of the IF, whereas low number of dispersed CSCs and PCs freely migrate outwards the tumor, creating an irregular outer boundary. Section B.2 of the S1 Supplementary Material includes other two cases: in panel A, CSCs do not migrate much faster than PCs; in panel C, CSCs are slower than PCs. Table 4 shows the parameters that correspond to panel A of Fig 7, and panels A and C of Section B.2 of the S1 Supplementary Material: slow tumor growth (*k*_C1,growth_ = 0.005 day^-1^) and R << 1 (R = 1/50), medium growth (*k*_C1,growth_ = 0.01 day^-1^) and R = 1, and fast growth (*k*_C1,growth_ = 0.015 day^-1^) and R >> 1 (R = 50). Besides the CSC and PC migration rates relation, we observe another factor strongly related to the shape of the IF. CD8+ T cells in cytotoxic state (red dots) are only located inside the tumor or at the IF. A few can be observed in the normal tissue, but within a very short distance from the outer boundary of the IF. Since that state only takes place at the proximity of cancer cells, we posit that their identification in real tumor samples could significantly contribute to determination the outer limit of the IF for potential resection. It is important to notice that Figs 7A and S5A and S5C, have been generated from thick slices of tumor (4x4x0.4 mm) projected onto a 2D plane. S6A Fig includes three examples of thinner slices when the tumor growth is slow (4x4x0.02 mm).

**Table 4:** Combinations of ratios *k*_C1,growth_*/k*_C,T1_, *k*_T1_*/k*_Treg_, and the parameter *T*_reg,max_, where *k*_C1,growth_, *k*_C,T1_, *k*_T1_, *k*_Treg_, and *T*_reg,max_ are the cancer cell growth rate, the rate of cancer cell death by T cells, the exhaustion rate of cytotoxic T cells by all cells that express PD-L1, the inhibition rate of cytotoxic T cells by regulatory T cells, and the maximal regulatory T cell density in the tumor, respectively. G.R. stands for growth rate.

	Slow G.R.	Medium G.R.	Fast G.R.	*Definition*
***R***	1/50	1	50	Ratio dependent on proliferation and migration rates of stem-like and progenitor cancer cells.
***k***_**C1,growth**_ ***/k***_**C,T1**_	7 *x* 10^*−*3^	7 *x* 10^*−*3^	1.1 *x* 10^*−*2^	Ratio cancer cell growth to cancer cell death by T cells.
***k***_**T1**_ ***/k***_**Treg**_	0.56	1.12	1.12	Ratio cytotoxic T cell exhaustion by PD-L1 to exhaustion by regulatory T cells.
***T***_**reg,max**_ **(cell/mL)**	9 *x* 10^5^	2 *x* 10^6^	2 *x* 10^6^	Maximal regulatory T cell density in the tumor.

Fig 7B shows the three-dimensional map of cell density distributions of CD8+ T cells and FoxP3+ T cells where the IF region, the central tumor region (CT), and the normal tissue (N) are depicted in red, yellow, and green colors, respectively. CT and N regions are inside the IF inner boundary and outside the IF outer boundary, respectively. Cell density values are generated by using the R function *density*.*ppp* (https://www.rdocumentation.org/packages/spatstat.core/versions/2.1-2/topics/density.ppp) that computes a kernel smoothed intensity function from a point pattern. Cancer cell densities have been expressed so far with the dimensionless parameter *ρ*, however, we represent T cell density distributions and profiles in terms of spatial units in order to compare them with the analysis performed in [28]. Thus, we divide the output from the R function *density*.*ppp* by the dimensional factor *L*^*2*^ where *L* is the length of the edge of a cubic voxel in the spatial grid. Additionally, due to some model limitations, we multiply densities by an augmentation factor λ = 6 (see *Technical limitations of the QSP-ABM coupling* in Discussion section). Hence, all cell density distributions and profiles are generated by assuming the same value for λ.

Fig 7C and 7D show CD8+ T cell and FoxP3+ T cell density profiles, similar to the ones presented in [28], a recent digital pathology study of triple-negative breast cancer. The three rows of density profiles in panels C and D correspond to the three scenarios from Table 4: slow tumor growth, medium growth, and fast growth. The procedure to generate T cell density-distance profiles in panel C assumes that cancer cell density decreases monotonically from core to normal tissue and it is defined as follows: 1) cancer cell density values are generated from cancer cell locations by computing gaussian kernel density estimates; 2) wide bandwidths for the kernels are selected at regions close to the tumor core (high number of cells, no significant gradients) and narrower bandwidths as we get closer to the IF (decreasing number of cells, high gradients); 3) cancer cell density values are ordered from highest to lowest and generate a list of their spatial locations following the same order; 4) T cell density values assigned to specific locations are reordered following the same order of the list generated in step 3 for cancer cell density locations. The result is an average T cell density-profile perpendicular to the IF. For Fig 7D, we only take the T cell density values that correspond to the locations where the IF is defined. Then, we divide the space along the azimuthal direction and apply a similar procedure to generate average T cell densities for each one of those divisions. The result is a T cell density-profile along the IF. All density profiles are multiplied by the augmentation factor λ.

In Fig 7C, we represent the IF region as a space of w_pathol_=1 mm wide between red lines and a narrower space of w_pathol_=0.5 mm wide between blue lines, corresponding to the conventional widths recognized by cancer pathologists. The location of the red line at the left (inner boundary of the IF) is where the cancer cell density profile reaches a value equal to *ρ*_*max*,*IF*_, estimated in Section A.3 of the S1 Supplementary Material. Now, it is important to recall that to generate Fig 7A and 7B, we defined the outer boundary of the IF at the locations where the cancer cell density value $\rho_{cutoff}^{min}$ was reached. Nevertheless, the procedure to generate average T cell density-profiles in Fig 7C requires to define an average distance from the inner boundary to the outer boundary. Such average distance is assumed to be equal to the IF width used in cancer pathology, i.e., w_pathol_=1 mm. Thus, the red line at the right (outer boundary of the IF) is defined 1 mm far from the other one. The region between blue lines is defined at the center of the one between red lines. The densities at the left and right of the red lines are the densities at the CT and the N regions, respectively. Thus, we can see that the immune cell density profiles follow a spatial distribution similar to the profiles analyzed in [28]: density increasing in the CT region from the center of the tumor to the inner boundary of the IF, where it reaches a maximum value; density decreasing from the inner boundary to the outer boundary of the IF (the 1 mm space between red lines); density keeps decreasing to the minimum value that corresponds to the N region. This decrease outside the IF is also observed in some tumor samples [28]. Interestingly, we see that it is significant in the first two cases (first and second row of panel C), but not in the third one (third row of panel C). Consequently, we posit that some T cells get recruited beyond the 1 mm region when the CSCs migration rate is larger than the PCs migration rate because a low number of CSCs migrate fast enough to create invasive low-density fingering structures (cancer cell distributions in Figs 7A and S5A). The stochastic effects are larger in FoxP3+ T cell density profiles than in CD8+ T cell profiles because the density of the former is lower in the presented cases. Additionally, we observe that, although the second case does not have the slowest tumor growth rate, the IF region advances a shorter distance than the other two cases.

### Cell distribution at the IF and T cell infiltration profiles with immunotherapy

Fig 8A presents the spatial distribution of all subtypes of cancer cells, CD8+ T cells, FoxP3+ T cells, and MDSCs in a section of a tumor slice 6 months after the initial diameter condition is met, with 3 mg/kg nivolumab administered every two weeks. The tumor size gets reduced, the IF becomes a wider region occupied by both CSCs and PCs, and a significant number of cytotoxic and suppressed CD8+ T cells are present at the IF. Low-density fingering structures are still present after immunotherapy is applied. Table 4 shows the parameters that correspond to panel Fig 8A, and panels B and D of Section B.2 of the S1 Supplementary Material: slow tumor growth (*k*_C1,growth_ = 0.005 day^-1^) and R << 1 (R = 1/50), medium growth (*k*_C1,growth_ = 0.01 day^-1^) and R = 1, and fast growth (*k*_C1,growth_ = 0.015 day^-1^) and R >> 1 (R = 50). Interestingly, for the first two cases, the IF gets wider and the locations of CD8+ T cell in cytotoxic state do not correspond to the IF spatial profile after treatment. However, immunotherapy does not affect neither the IF width nor the spatial distribution of cytotoxic CD8+ T cells in the third case. Figs 8A and S5B and S5D have been generated from thick slices of tumor (4x4x0.4 mm) projected onto a 2D plane. S6B Fig includes three examples of thinner slices (4x4x0.02 mm) when the tumor growth is slow.

Fig 8B includes the three-dimensional cell density distributions of CD8+ T cells and FoxP3+ T cells. CT and N regions are inside the IF inner boundary and outside the IF outer, respectively. Cell density values are generated from cell locations by computing gaussian kernel density estimates [53] and multiplied by the augmentation factor λ.

Finally, Fig 8C shows that immunotherapy introduces the following effects in the T cell density profiles: 1) higher CD8+ T cell densities and lower FoxP3+ T cells densities, i.e., CD8+ T cells to FoxP3+ T cells ratio increases with treatment; 2) high stochasticity in FoxP3+ T cells density profiles and disappearance of the characteristic maximum density at the inner boundary of the IF; 3) higher displacement of the IF region to the left in the first two cases than in the third one; 4) wider region of significant T cell density outside the red lines in the first two cases but negligible in the third case (see also cancer cell distributions in Figs 8A and S5A and S5B). Panel D shows that immunotherapy increases the cell density heterogeneity along the IF in all cases since oscillations happen with more frequency than without treatment. All density profiles are multiplied by the augmentation factor λ.

## Discussion

### Modeling methodology

One of the goals of this study is to introduce a methodology to combine a deterministic ODE-based QSP model with a stochastic ABM ensuring both simplicity and self-consistency. The QSP model describes the continuous temporal evolution of the average number and/or concentration of species, whereas the ABM provides information about the random spatial dynamics of individual entities over time. Besides their different nature, the species and reaction mechanisms are not the same in QSP model and ABM since some cell subtypes are only explicitly represented in the latter. Nevertheless, by formulating ODE versions of ABM rules and using propensity and probability functions based on QSP parameters and equations, we assure a simple coupling methodology for future extensions or applications of this model (e.g., developing an ABM version of the QSP lymph node compartment, adding new cells, building spQSP versions of existing QSP models of other type of cancers). Additionally, this approach minimizes the calibration of new parameters in ABM and guarantees consistency between QSP model and ABM.

### Technical limitations of the QSP-ABM coupling

The presented spatial QSP model is the combination of a whole-patient ordinary differential equations-based QSP model and a spatial ABM that represents either part of a tumor or a coarse-grained approximation of the entire tumor. We can choose one approach or the other depending on the spatial scale and level of accuracy that we are interested in as well as the applications of the model.

#### ABM representation of part of a tumor

When ABM is used to depict part of the tumor, the scaling factor *γ* represents how many times such part is smaller than the entire tumor (in number of cells). The number of cells in ABM multiplied by *γ* provides the number of cells in QSP at every time step. Similarly, the scaling factor *γ* is used to define the proportion of cells (1/*γ*) that get recruited from blood (central QSP compartment) into ABM. Since the QSP model calculates the total average number of cells in the tumor for each species, the QSP-ABM coupling implies that ABM represents a region where the number of cells is 1/γ times the total average. Thus, this coupling has a significant limitation. If, for instance, we want to simulate ROIs where the number of T cells is much larger than the average, i.e., hotspots, ABM would underestimate the number of cells in those ROIs. Examples of such limitation are the density profiles from Fig 7C and 7D that have been obtained from thick slices of tumor (4x4x0.4 mm) projected onto a 2D plane, and multiplied by an augmentation factor λ of order 1-10 for the purpose of representing density profiles similar to [28]. Digital pathology images are thinner than that and the slices from section B.3 of the S1 Supplementary Material (4x4x0.02 mm) would be a more accurate representation of real samples. Nevertheless, we do not generate density profiles from these simulated thin slices since they are sparsely populated and, consequently, the densities would be much lower and the stochastic variations much larger than in real hotspot samples.

A special case is γ=1; when this scaling factor is chosen, the tumor is entirely represented in ABM (Figs 3 and S4). Although this scenario is highly descriptive for initial tumor growth stages, it becomes unmanageable for large tumors because of the high computational cost that a sizable grid and the representation of millions of cells would require.

#### ABM as a coarse-grained model for the whole tumor

If ABM is used to describe the growth dynamic of the entire tumor, each agent is assumed to contain a group of cells and the scaling factor γ is the number of cells that that agent represents. The QSP-ABM coupling guarantees that the ABM representation follows the same macroscopic temporal dynamics as the QSP model. Spatially, the depiction of large groups of cells as agents introduces randomness that a macroscopic model would not capture. Thus, the accuracy of the model is inversely related to the value of γ, and the spatial dynamics gets restricted by the number of agents that comprise the entire system. This coarse-grained approach requires the multiplication of migration rates of agents by 1/γ^1/3^, a scaling factor that represents the fraction of cells that would move in a specific direction in a 3D grid if we assume that γ is the number of cells inside a voxel occupied by an agent. Because of these limitations, the spatial distribution of agents gives us a rough picture of the tumor that we present in terms of cell density distributions (Figs 4 and 5). However, accurate analysis of the invasive front is not viable with this approach.

### Potential improvements in the tumor growth description

The accuracy of the spQSP model to describe tumor shape and IF properties is not only limited by the modeling approaches (e.g., deterministic/stochastic, macroscopic/microscopic), but also by the species and mechanisms that are explicitly represented.

The significant role of the extracellular matrix (ECM) in the evolution of solid cancers is not taken into account explicitly in the spQSP model [73]; implicitly, its effects are reflected in the parameters such as migration and proliferation rates. Induced ECM components are dynamic during progression, and promote invasion and metastasis [74,75]. ECM also mediates resistance of cancer cells to existing treatments [76,77]. Future extensions of the model could be combined with detailed spatial representations of the ECM fiber network such as [78] and [79] in order to elucidate its influence on tumor shapes and cell migration patterns. Fundamental elements of the tumor microenvironment such as macrophages and fibroblasts are also crucial in tumor progression [30].

Although, we define a probabilistic T cell extravasation mechanism from blood that depends on the local cancer cell density, the angiogenesis process and the tumor vasculature network [80,81] are not explicitly included in this model. Adding a detailed capillary-scale complete network at the tumor scale would require very significant computational costs. However, the same modeling approaches proposed for the representation of agents and mechanisms in the spQSP could be applied to implement tumor vasculature in the model: coarse-grained approximation and refined description of the vessel network at the tumor scale and the invasive front scale, respectively. There is an extended literature regarding modeling angiogenesis and tumor vasculature as well as their role in invasive and metastatic processes that could be used to implement these mechanisms [81].

Besides mechanistic models, recent artificial intelligence and deep learning approaches for whole-slide image segmentation are becoming indispensable for the spatial analysis of pathological samples [82,83]. They provide unique data about the components of the tumor microenvironment, the tumor boundaries, and the response to treatment. This highly valuable information would be undoubtedly useful for future spQSP calibration, model improvement, and validation processes.

## Conclusions

Quantitative Systems Pharmacology, pharmacokinetics (PK) and physiologically based pharmacokinetic (PBPK) models have been used to represent the dynamics of drug transport in patients in conjunction with ABM to predict the disease trajectories as different treatment strategies are applied. By capturing the histopathology with agent-based models, these systems recapitulate the spatiotemporal disease dynamics in high-resolution; however, the computational cost can be prohibitive, especially in scenarios involving tissues of larger size and longer time frames.

Besides the representation of macroscopic processes with ordinary-differential-equations-based QSP models (ODE-based), and the depiction of spatially heterogeneous systems with ABM, some approaches also capture the development of agents (either single entities or clusters) with systems of ODEs [84,85]. Others include partial differential equations (PDEs) in combination with QSP and ABM in order to visualize the spatial dynamics of some species, without adding the stochastic level of detail and complexity of ABM [33,36,37,86]. Although, these studies include alternative approaches to ours, the optimal model depends on the purpose of the study. The systems, processes, species, and reactions that comprise a hybrid model need to be *a priori* analyzed one by one to decide the appropriate accuracy-to-computational-cost ratio. Similarly, the number of elements represented in the model need to be properly assessed since the computational cost and the data required for parameterization and validation increase as the model gets developed.

In this study, we have extended a recently published spatial QSP platform where a QSP model and an ABM are combined to represent the spatial heterogeneity of the tumor microenvironment. The original hybrid approach defined the state of the species in the tumor by combining the outcomes from QSP model and ABM. Here, however, the limited QSP representation of cells in the tumor is fully replaced by the ABM spatial representation after applying a scaling factor. This establishes a simple methodology to transform QSP models into their equivalent spatial representations. It also guarantees consistency between QSP model and ABM to estimate the overall behavior of the tumor when ABM is used as a coarse-grained version of the tumor and when it is used to represent specific regions of interests (parts of the tumor) where cells are tracked as individual entities. The dynamic cell recruitment in ABM from the QSP blood compartment that is implemented in this extended version provides a clearer picture of the spatial heterogeneity of the immune response without and with immunotherapy.

Our conclusions can be summarized as follows:

- Analysis of tumor growth based on cancer cell properties has been performed to characterize physical tumor features and their evolution over time.
- The extended spQSP model combines the characteristics of two methods that are used for CD8+ T cell enumeration in prognosticating TNBC: hotspot versus whole-tumor. It provides information at the whole-tumor scale while estimating the regions where the main hotspots are located.
- spQSP model is able to characterize the heterogeneous evolution of the invasive front under different tumor growth and immune response conditions (formation of fingering structures, budding morphology, cluster formation, etc.).
- The spatial analysis shows the formation of clusters at the whole-tumor scale as well as higher heterogeneity at the invasive front when immunotherapy is applied.
- The relative location of cancer stem-like cells and progenitor cells as well as the location of cytotoxic CD8+ T cells could be helpful in interpretations of digital pathology images to define the outer boundary of the invasive front.
- The study paves the way to full integration of spatial QSP modeling and multiplex digital pathology for model parameterization and validation, as well as biomarker discovery.
- Both model simulations of the tumor microenvironment and the corresponding digital pathology images can be analyzed using methods of spatial statistics and deep learning.

## Supporting information

S1 Supplementary Material Additional calculations, analysis, and results. (DOCX)

S1 Fig QSP solutions for 100 cases without treatment after recalibration. (TIF)

S2 Fig QSP solutions for 100 cases with 3 mg/kg biweekly Nivolumab (anti-PD-1) treatment after recalibration. (TIF)

S3 Fig Comparison of QSP and spatial QSP solutions. (TIF)

S4 Fig Spatio-temporal evolution of the tumor. (TIF)

S5 Fig Spatial representation of cancer cell subtypes, CD8+ T cell subtypes, FoxP3+ T cells, and MDSCs in a section of a tumor slice. (TIF)

S6 Fig Spatial representation of cancer cell subtypes in a section of a tumor slice with slow growth and R<<1. (TIF)

S1 Table QSP model reactions, expressions, and parameter values after recalibration. (XLSX)

S2 Table Set of QSP parameters and ABM assumptions, parameters, and expressions used for the results presented in this paper. (XLSX)
