# Supplementary material for: Simulations of tumor growth and response to immunotherapy by coupling a spatial agent-based model with a whole-patient quantitative systems pharmacology model
Source: PLoS Comput Biol. 2022 Jul 22;18(7):e1010254. doi: 10.1371/journal.pcbi.1010254 (PMC9348712; doi:10.1371/journal.pcbi.1010254)
Supplement: S1 Supplementary Material — (DOCX) [file pcbi.1010254.s001.docx]

# SUPPLEMENTARY MATERIAL

##
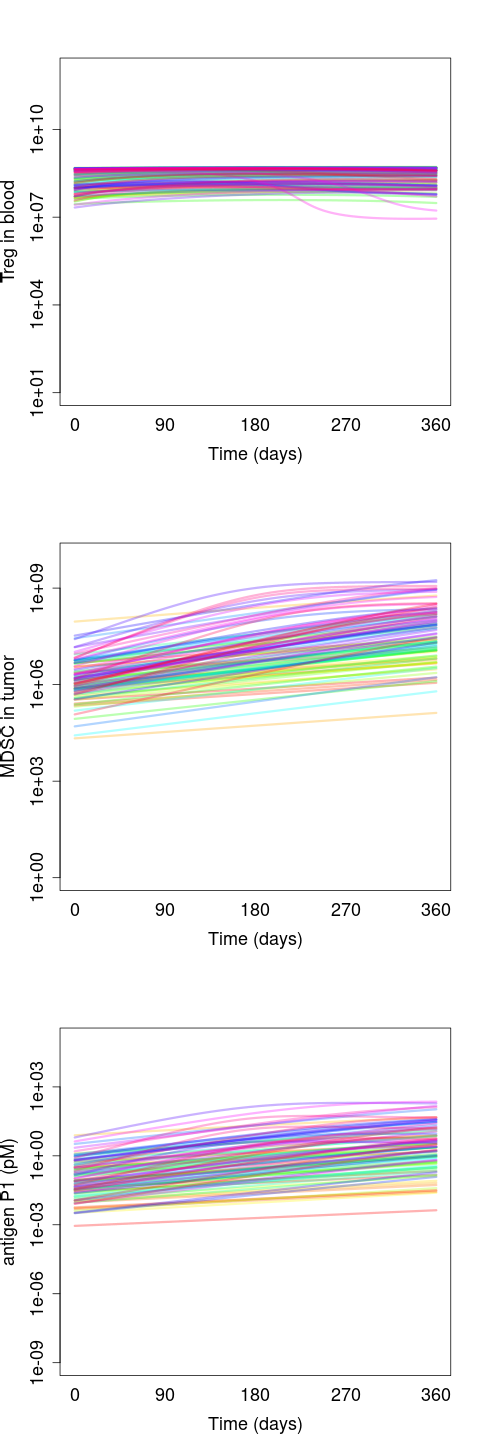

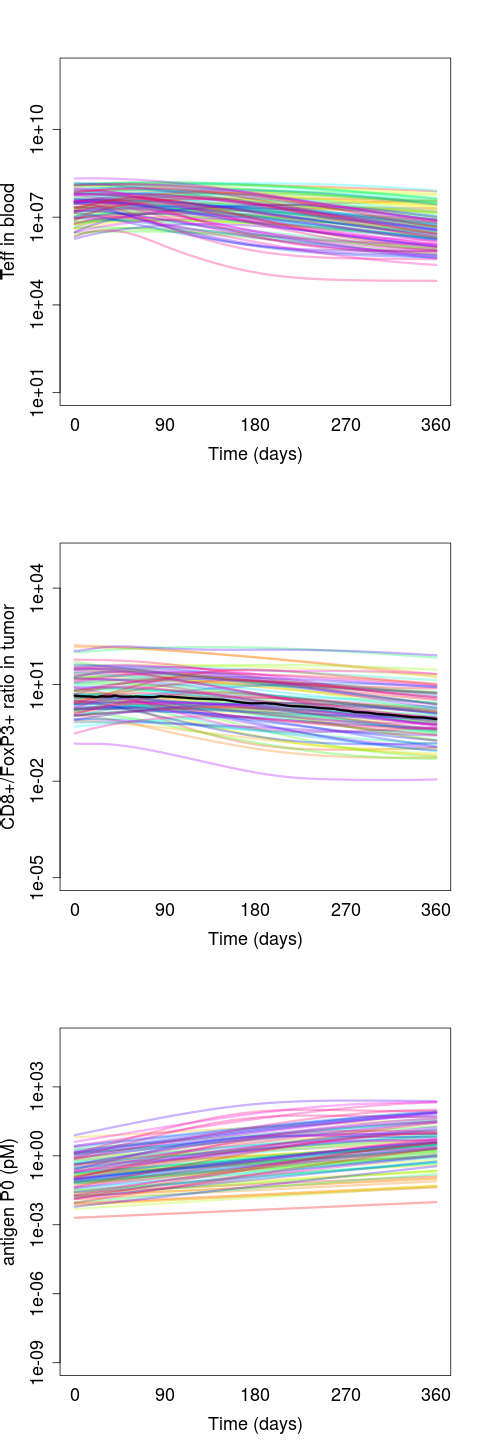

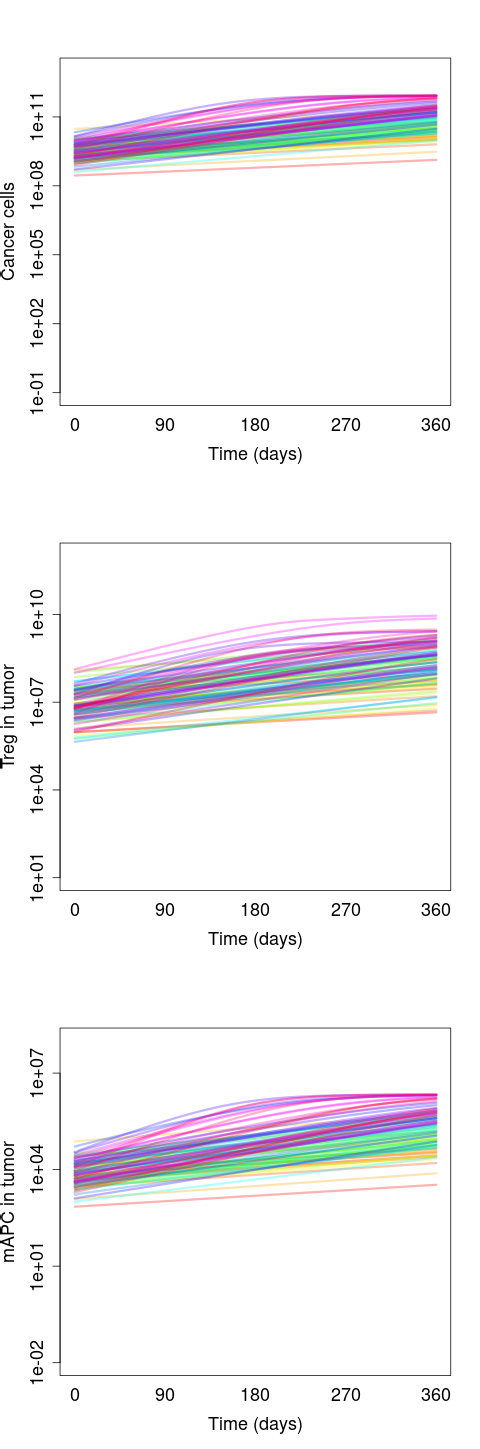

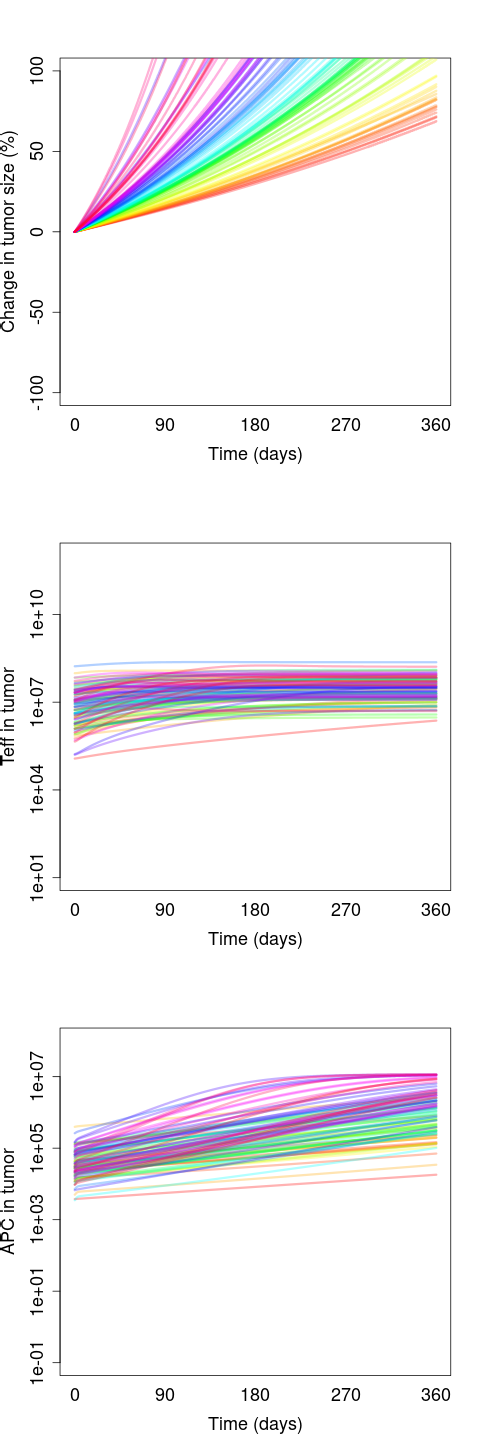
QSP model recalibration

Figure S1: QSP solutions for 100 cases without treatment after recalibration.


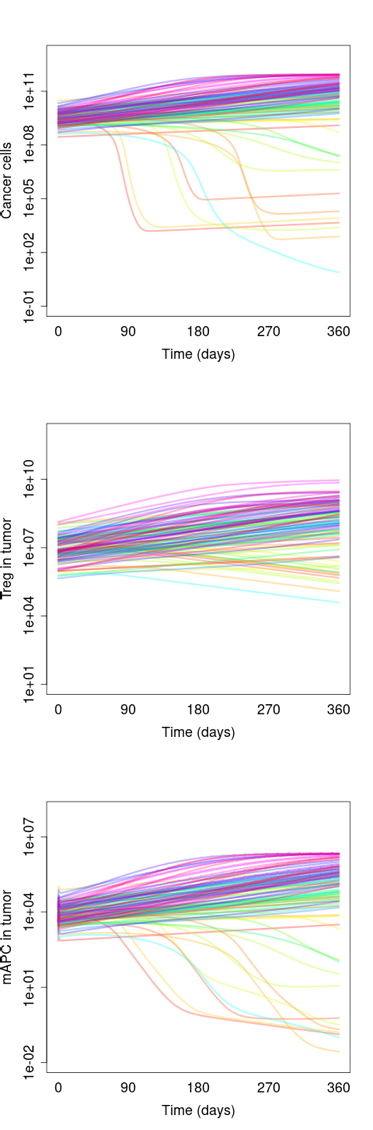

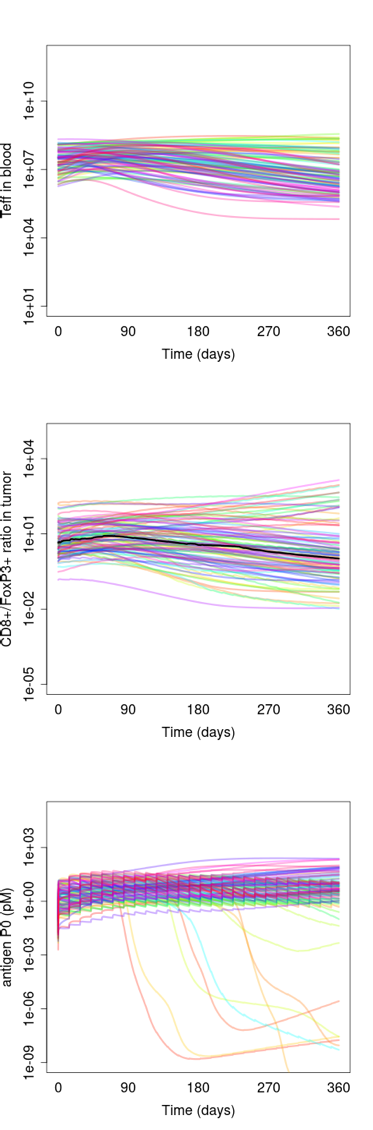

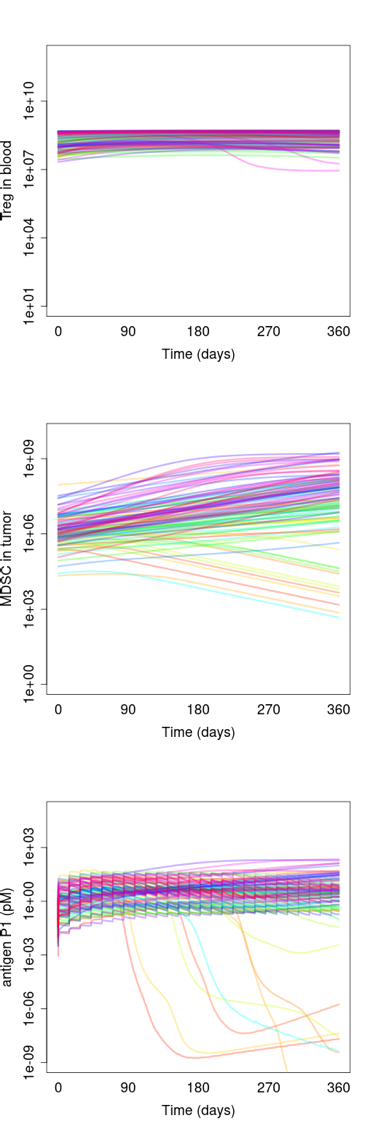

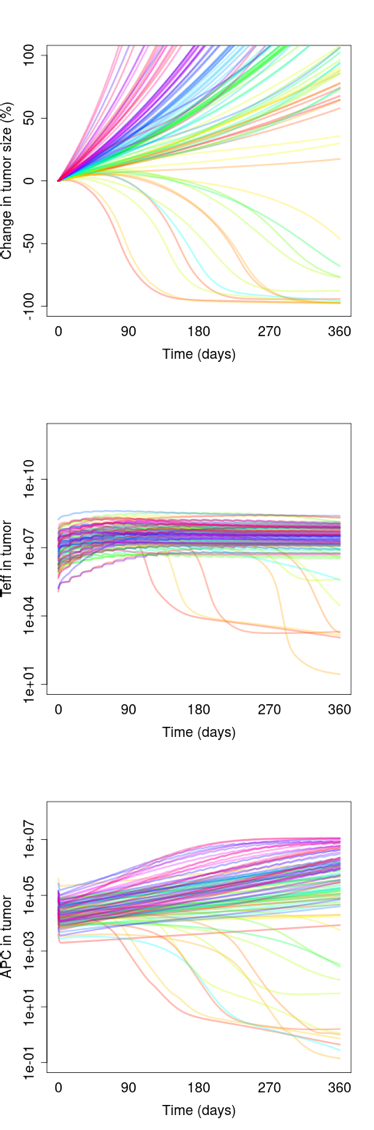


Figure S2: QSP solutions for 100 cases with 3 mg/kg biweekly Nivolumab (anti-PD-1) treatment after recalibration.

## Cell migration and reaction rates

*Cell migration*

The probability of migration of a cell in a time step τ, $p_{mig,cell}$, depends on the migration rate, $u_{cell}$, such that

$$p_{mig,cell}=\frac{\tau}{Lv_{m}}u_{cell}, (S1)$$

where *L* is the length of the edge of a cubic voxel in the spatial grid and *v_m_* is the maximum number of voxels that the fastest cell (highest migration rate) would cross during that time step. The migration rates for all cells,$u_{cell}$, are taken from literature (see Supplemental Table 2 for references) and the input *v_m_* must be any random integer number larger than 0 such that the condition $p_{mig,cell}\leq1$ is met for all cells. For instance, if we suppose that the fastest cell moves every time step, i.e., $p_{mig,cell}^{max}=1$, from equation (*S*1) we can assume that $v_{m}\approx\left\lfloor\tau u_{cell}^{max}/L \right\rfloor$, where $\left\lfloor x \right\rfloor$ is the floor function that gives the largest [integer](https://mathworld.wolfram.com/Integer.html) less than or equal to x. Thus, for each cell and each time step, ABM checks the condition $p_{mig,cell}\geq\xi_{U}$*v_m_* times, where $\xi_{U}$ is a random number from a uniform distribution on the interval [0,1], and moves the cell every time such condition is met.

*Cancer cells growth and death*

Below, we formulate an ODE version of the ABM rules for cancer cell growth dynamics. Here, *S_t_*, *P_i_*, and *S_e_* refer to the number of cancer stem-like cells, progenitor cells after *i* divisions, and senescent cells, respectively, in ABM. Assuming that cancer stem-like cells and progenitor cells proliferate at rates *r_st_* and *r_p_*, respectively, and recalling the probability of division *k*, the maximum number of divisions *d_max_*, and the death rate *µ*, we describe the dynamics of cancer cell subtypes as follows

$$\frac{{dS}_{t}}{dt}=r_{st}\left( 1-k \right)S_{t}, (S2)$$

$$\frac{{dP}_{1}}{dt}={kr}_{st}S_{t}-r_{p}P_{1}, (S3)$$

$$\frac{{dP}_{i}}{dt}=r_{p}\left( 2P_{i-1}-P_{i} \right) for 2\leq i\leq d_{max}, (S4)$$

$$\frac{{dS}_{e}}{dt}=2r_{p}P_{d_{max}}-\mu S_{e}. (S5)$$

Combining equations (*S*2) and (*S*3), we obtain

$$\frac{d}{dt}\left( P_{1}-pS_{t} \right)=-r_{p}\left( P_{1}-pS_{t} \right), (S6)$$

where

$$p=\frac{kr_{s}}{r_{st}\left( 1-k \right)+r_{p}} (S7)$$

As the tumor grows, the expression *P*_1_ *– pS_t_* from equation (*S*5) tends to zero such that

$$\frac{P_{1}}{S_{t}}\to p (S8)$$

Similarly, the relationships between other cancer cell subtypes also lead to constant ratios as time increases such that

$$\frac{P_{i}}{P_{i-1}}\to\frac{2pr_{p}}{kr_{st}}=l_{1} for 2\leq i\leq d_{max} , (S9)$$

$$\frac{S_{e}}{P_{d_{max}}}\to\frac{2r_{p}}{\left( 1-k \right)r_{st}+\mu}=l_{2}, (S10)$$

and the three cancer cell subtypes grow with a rate *k_C1,growth_ =(1-k)r_st_* that is the cancer cell growth rate from the QSP model. Additionally, for consistency between QSP model and ABM, the saturation factor *1-C/C_max_* from the QSP model, where *C* denotes the number of cancer cells in the QSP tumor and *C_max_* is the maximum capacity of the tumor, is multiplied by the proliferation rates *r_st_* and *r_p_* in ABM.

Table S1: Fractions of cancer stem-like cells, progenitor cells, and senescent cells where

$$q=1+p\sum_{i=1}^{d_{max}} l_{1}^{i-1}+pl_{1}^{d_{max}-1}l_{2}=1+\frac{p(l_{1}^{d_{max}}-1)}{l_{1}-1}+pl_{1}^{d_{max}-1}l_{2}.$$

|  | *S_t_* | *P_i_* | *S_e_* |
| --- | --- | --- | --- |
| Fraction | $\frac{1}{q}$ | $\frac{pl_{1}^{i-1}}{q}$ | $\frac{pl_{1}^{dmax-1}l_{2}}{q}$ |

Because of the long-term constant ratios between cancer cell subtypes, the fractions of each species among all cancer cells remain constant and take the forms presented in Table S1. The grid is populated with cancer cells at the beginning of the simulation according to those proportions.

*Decay reaction rates*

Generally, the decay reactions in the QSP model are defined in terms of the deterministic variation in time of the number of cells of a generic species *S* as follows

$$\frac{dS}{dt}=-k_{d}S, (S11)$$

where *k_d_* denotes a decay reaction rate. Integrating between an initial time, *t*_0_, with an initial number of cells, *S*_0_, and a final time, *t*_0_ + *τ*, with a variable number of cells, *S*, we obtain

$$S=S_{0} exp(-k_{d}\tau). (S12)$$

Assuming that *S* and *S*_0_ are the continuous equivalent of the discrete number of cells, *n* and *n*_0_, equation (*S*11) can be reformulated as the decay of a number of cells in a time step *τ*,

$$\frac{n_{0}-n}{n_{0}}=1-exp(-k_{d}\tau). (S13)$$

This expression shows that the probability of decay of one cell, *p_reaction_*, in a time step *τ* can be expressed as

$$p_{reaction}=1-exp(-a_{reaction}\tau) (S14)$$

where *a_reaction_* is the propensity of the reaction. Thus, the stochastic equivalent of deterministic reaction rates can be defined as propensity functions based on population of species [1,2,3,4]. Defining *ξ_U_* as a random number from a uniform distribution on the interval [0,1], the reaction is supposed to happen during the time step *τ* if the condition *p_reaction_* *> ξ_U_* is met.

1. Death of cancer cells by cytotoxic T cells

$$QSP expression: \frac{dC}{dt}=-k_{C,T1}\frac{T_{1}}{C+T_{total}+cell}\left( 1-H_{PD1,C} \right)\left( 1-H_{MDSC,C} \right)C (S15)$$

where *T*_1_, and *T_total_* are the effector T cells, and the total number of T cells in the QSP tumor compartment, respectively, “*cell*” is a parameter equal to 1 introduced by Wang et al. [5] in the denominator to avoid singularities, *k_C,T_*_1_ is the rate of cancer cell death by effector T cells in the QSP tumor compartment, *H_PD_*_1_*_,C_* is the Hill function of PD-1/PD-L1 inhibition by cancer cells, and *H_MDSC,C_* is the Hill function of inhibition by MDSCs.

$$ABM propensity: a_{C,T1}=k_{C,T1}\frac{{N_{T}}_{cyt}}{N_{C}+{N_{T}}_{total}+N_{cell}}\left( 1-H_{PD1,C} \right)\left( 1-H_{MDSC,C} \right) (S16)$$

$$ABM probability: p_{C,T1}=1-exp(-a_{C,T1}\tau) (S17)$$

where *k_C,T_*_1_ is the rate of cancer cell death by cytotoxic T cells in ABM and *N_Tcyt_* */*(*N_C_* + *N_Ttotal_* + *N_cell_*) is the fraction of cytotoxic T cells, *N_Tcyt_*, among all surrounding cells of a cancer cell, *N_C_* and *N_Ttotal_* are the total number of surrounding cancer cells and T cells in ABM, respectively, and *N_cell_* equals 1 to avoid singularities.

2) Death of regulatory T cells

$$QSP expression: \frac{dT_{0}}{dt}=-k_{T0,death}T_{0} (S18)$$

where *T*_0_ and *k*_T_*_0,death_* are the total number of regulatory T cells in the QSP tumor compartment and their death rate, respectively.

$$ABM propensity: a_{T0,death}=k_{T0,death} (S19)$$

$$ABM probability: p_{T0,death}=1-exp(-a_{T0,death}\tau) (S20)$$

1. Cytotoxic T cell exhaustion

3a) Inhibition of cytotoxic T cells by regulatory T cells

$$QSP expression: \frac{dT_{1}}{dt}=-k_{Treg}\frac{T_{0}}{C+T_{total}+cell}T_{1} (S21)$$

where *k_Treg_* is the inhibition rate of effector T cells by regulatory T cells in the QSP tumor compartment.

$$ABM propensity: a_{Treg}=k_{Treg}\frac{{N_{T}}_{reg}}{N_{C}+{N_{T}}_{total}+N_{cell}} (S22)$$

$$ABM probability: p_{Treg}=1-exp(-a_{Treg}\tau) (S23)$$

where *k_Treg_* is the inhibition rate of cytotoxic T cells by regulatory T cells in ABM and *N_Treg_ /(N_C_ + N_Ttotal_ + N_cell_*) is the fraction of regulatory T cells, *N_Treg_* , among all surrounding cells of a cytotoxic T cell.

3b) Exhaustion of cytotoxic T cells from PD-L1 interaction

$$QSP expression: \frac{dT_{1}}{dt}=-k_{T1}\frac{C}{C+T_{total}+cell}{H_{PD1,C}T}_{1} (S24)$$

where *k_T_*_1_ is the exhaustion rate of effector T cells by cancer cells in the QSP tumor compartment.

$$ABM propensity: a_{T1}=k_{T1}H_{PD1,C} (S25)$$

$$ABM probability: p_{T1}=1-exp(-a_{T1}\tau) (S26)$$

where *k_T_*_1_ is the exhaustion rate of cytotoxic T cells in ABM and the fraction *C/(C + T_total_ + cell*) from expression (*S*24) has been set to 1 for the propensity since we assume that all cells in the surroundings can express PD-L1.

In order to determine which exhaustion mechanism dominates, ABM calculates the fraction *χ* = *a_Treg_/*(*a_Treg_* + *a_T_*_1_). Following the Gillespie algorithm [1], ABM generates two random numbers from uniform distributions on the interval [0,1] per cytotoxic cell (*ξ_U,1_, ξ_U,2_*): *ξ_U,1_* indicates which reaction dominates, i.e., a cytotoxic T cell is exhausted by regulatory T cells if *ξ_U,1_* *< χ* or by PD-L1 interaction if *ξ_U,1_* *≥ χ*; *ξ_U,2_* determines if the exhaustion happens in the time step *τ*, i.e., *p_Treg_* *> ξ_U,2_* for inhibition by regulatory T cells or *p_T_*_1_ *> ξ_U,2_* for interaction with PD-L1.

4) Death of effector and cytotoxic T cells

$$QSP expression: \frac{dT_{1}}{dt}=-k_{T1,death}T_{1} (S27)$$

where *k_T_*_1_*_,death_* is the death rate of effector T cells in the QSP tumor compartment.

$$ABM propensity: a_{T1,death}=k_{T1,death} (S28)$$

where *k_T_*_1_*_,death_* is the death rate of effector and cytotoxic T cells in ABM.

$$ABM probability: p_{T1,death}=1-exp(-a_{T1,death}\tau) (S29)$$

1. ) Death of MDSCs

$$QSP expression: \frac{dMDSC}{dt}=-k_{MDSC,death}MDSC (S30)$$

where *k_MDSC,death_* is the death rate of MDSCs.

$$ABM propensity: a_{MDSC,death}=k_{MDSC,death} (S31)$$

$$ABM probability: p_{MDSC,death}=1-exp(-a_{MDSC,death}\tau) (S32)$$

*Recruitment reaction rates*

This model does not explicitly include the tumor vasculature, therefore in order to represent dynamic recruitment, we express the probability of extravasation of cells from blood to tumor at the invasive front (IF), *p*_rec,_*_IF_*, as a function linearly dependent on the normalized local cancer cell density, *ρ*,

$$p_{rec,IF}=p_{max}-\frac{p_{max}-p_{min}}{\rho_{max,IF}-\rho_{min,IF}}\left( \rho_{max,IF}-\rho\right), (S33)$$

where *ρ_min,IF_* and *ρ_max,IF_* are the normalized minimum and maximum local cancer cell densities at the locations where cells are recruited, respectively, and *p_min_* and *p_max_* are the probabilities of recruitment at the locations with minimum and maximum local cancer cell densities, respectively.

The four parameters in equation (*S*33), *ρ_min,IF_*, *p_min_*, *ρ_max,IF_*, and *p_max_*, are input parameters in ABM. We choose *ρ_min,IF_* = 0 since that would be the outer limit of the IF, i.e., the transition to the normal tissue. *p_min_* (or *p_rec,normal_*) should be low since it represents the probability of cell recruitment in the normal tissue. For maximum values, we choose *p_max_* = 1 as the probability of recruitment at the inner boundary of the IF. Digital pathology analysis from TNBC samples shows that the highest T cell density is reached at the inner boundary of the IF [6] and, also, it has been observed that total immune content is highly correlated with the amount of vascular endothelium cells in TNBC [7]. *ρ_max,IF_* is assumed to be neither high (close to the core) nor low (close to the normal tissue). A proper and accurate estimate of this value is highly significant when the spatial QSP model is used to represent detailed ROIs at the IF. In Section A.3 of the Supplementary Material, we provide analysis related to this parameter and the boundaries of the IF. Regarding the core of the tumor, we assume that it is highly crowded, and define the input parameter *p_rec,core_* as the probability of recruitment in locations where  *ρ* = 1.

Now, we define the normalized local cancer cell density, *ρ*, as the non-normalized density in a cubic volume comprised of voxels, *ρ_nn_*, divided by the density in a voxel, *ρ_v_*, as follows

$$\rho=\frac{\rho_{nn}}{\rho_{v}}=\frac{Cancer cells in a cubic volume/(Voxels in a cubic volume\times Voxel volume)}{Cancer cells in a voxel/(Voxel volume)}, (S34)$$

where *Cancer cells in a voxel* = 1. After simplifying (*S*34), we get the expression

$$\rho=\frac{Cancer cells in a cubic volume}{Voxels in a cubic volume}, (S35)$$

in order to represent the normalized local cancer cell densities as the number of cancer cells in a cubic volume comprised of voxels divided by the sum of all voxels contained in a cubic volume (27 voxels if the cubic volume is 3×3×3 voxels, 125 if the volume is 5×5×5 voxels, and so on). Thus, for cell recruitment representation, the ABM algorithm operates as follows:

1. At the beginning of the simulation:
2. A number of potential T cell/MDSC recruitment sources are randomly distributed with no more than a recruitment source per voxel.
3. At each time step:
4. For each potential T cell/MDSC recruitment source:

b.1. The local cancer cell density, *ρ*, around the T cell/MDSC recruitment source is defined as the sum of cancer cells in a cubic volume of, for instance, 3×3×3 voxels (with the source at its center), divided by the total number of voxels in the cubic volume, i.e., 27. The size of the cubic volume is an input in ABM that depends on the balance between accuracy and computational speed that we prefer: the larger the size, the more accurate the calculations, but the higher the computational cost.

b.2. The generic probability of T cell/MDSC recruitment at the IF depends on the local cancer cell density, *ρ,* and is calculated by using the expression (*S*33).

1. Now, the potential number of recruitment sources that ABM allocates at the beginning of the simulation, *N_sources_*, is defined as the number of locations in the spatial grid where cells can potentially transition from the central compartment to the tumor compartment. However, at every time step the algorithm counts how many of these sources, *N∗_sources_*, meet either the conditions {*ρ ≥ ρ_max,IF_* and *p_rec,core_ > ξ_core_*} or {*ρ < ρ_max,IF_* and *p_rec,IF_ > ξ_IF_*}, where *ξ_core_* and *ξ_IF_* are random numbers from uniform distributions on the interval [0,1], to determine which fraction of the potential sources are in effect recruiting cells in the core or anywhere else, respectively.
2. The propensities of T cells and MDSCs in the central compartment to get recruited in a particular source are defined below as the total propensities divided by *N∗_sources_*. ABM eventually calculates the probabilities of recruitment of each T cell and MDSC in each potential source based on these propensities.
3. Recruitment of regulatory T cells

$$QSP expression: \frac{dT_{0,C}}{dt}=-q_{T0,T,in}V_{T}T_{0,C}\left( 1-\frac{T_{0}}{T_{reg,max}V_{T}} \right) (S36)$$

where *T*_0_*_,C_* is the number of regulatory T cells in the central compartment, *q_T_*_0_*_,T,in_* is the rate of regulatory T cells transport into the tumor compartment, and *T_reg,max_* is the maximal regulatory T cell density in the tumor. *V_T_* is the tumor volume expressed as follows

$$V_{T}=V_{T,min}+V_{cell}\left( C+C_{x} \right)+V_{Tcell}\left( T_{0}+T_{1}+T_{exh} \right), (S37)$$

where *V_T,min_*, *V_cell_*, and *V_Tcell_* are the cancer-free tumor compartment volume, the average volume of a cancer cell, and the average volume of a T cell, respectively, and *C_x_* and *T_exh_* are the number of dead cancer cells (not explicitly represented in ABM) and the number of suppressed/exhausted T cells, respectively.

$$ABM propensity: a_{T0,rec}=\frac{q_{T0,T,in}V_{T}T_{0,C}}{N_{sources}^{*}}\left( 1-\frac{T_{0}}{T_{reg,max}V_{T}} \right) (S38)$$

1. Recruitment of effector T cells

$$QSP expression: \frac{dT_{1,C}}{dt}=-q_{T1,T,in}V_{T}T_{1,C} (S39)$$

where *T*_1_*_,C_* is the number of effector T cells in the central compartment and *q_T_*_1_*_,T,in_* is the rate of effector T cells transport into the tumor compartment.

$$ABM propensity: a_{T1,rec}=\frac{q_{T1,T,in}V_{T}T_{1,C}}{N_{sources}^{*}} (S40)$$

1. Recruitment of MDSCs

8a) Base recruitment of MDSCs

$$QSP expression: \frac{dMDSC}{dt}=k_{MDSC,b,rec}({MDSC}_{max}V_{T}-MDSC) (S41)$$

where *MDSC* is the number of myeloid-derived suppressor cells in the QSP tumor compartment, *MDSC_max_* is the maximal MDSC density in the tumor, and *k_MDSC,b,rec_* is the baseline rate of MDSC migration into the tumor. The QSP model does not represent explicitly the number of MDSCs in the central compartment, but we assume that the recruitment rate is similar to the rate expressed in equation (*S*41).

$$ABM propensity: a_{MDSC,b,rec}=k_{MDSC,b,rec}\frac{{MDSC}_{max}V_{T}-MDSC}{N_{sources}^{*}} (S42)$$

8b) Recruitment of MDSCs by CCL2

$$QSP expression: \frac{dMDSC}{dt}=k_{MDSC,rec}({MDSC}_{max}V_{T}-MDSC)\frac{CCL2}{{EC50}_{CCL2,rec}+CCL2} (S43)$$

where *CCL*2 is the concentration of monocyte chemoattractant protein-1 in the tumor, *EC*50*_CCL_*_2_*_,rec_* is the half-maximal CCL2 level of MDSC recruitment, and *k_MDSC,rec_* is the rate of MDSC recruitment by CCL2 into the tumor. Again, we assume that the recruitment rate is similar to the rate expressed in equation (*S*43).

$$ABM propensity: a_{MDSC,rec}=k_{MDSC,rec}\frac{{MDSC}_{max}V_{T}-MDSC}{N_{sources}^{*}}\frac{CCL2}{{EC50}_{CCL2,rec}+CCL2} (S44)$$

Similarly to T cell exhaustion, for MDSC recruitment, ABM implements the Gillespie algorithm to determine which type of recruitment is predominant.

*Expansion reaction rate*

1. Regulatory T cells expand in the tumor according to the QSP reaction rate

$$QSP expression: \frac{dT_{0}}{dt}=k_{T0,exp}T_{0}\left( 1-\frac{T_{0}}{T_{reg,max}V_{T}} \right)\frac{ArgI}{{EC50}_{ArgI,T0}+ArgI}=R_{T0,exp} (S45)$$

where *ArgI* is the concentration of Arg-I in the tumor, *EC*50*_ArgI,T_*_0_ is the half- maximal Arg-I level of regulatory T cell expansion, and *k_T_*_0_*_,exp_* is the rate of Arg-I-induced regulatory T cell expansion.

ABM takes the expression (*S*45) to define the probability of expansion as follows

$$ABM probability: p_{T0,exp}=\left( 1-\frac{T_{0}}{T_{reg,max}V_{T}} \right)\frac{ArgI}{{EC50}_{ArgI,T0}+ArgI} (S46)$$

Thus, if *p*_T0,exp_ *> ξ_U_* where *ξ_U_* is a random number from a uniform distribution on the interval [0,1], the regulatory T cell divides a number of times inversely proportional to the reaction rate *RT*0*,exp*,

$$N_{T0,exp}=\frac{\ln2}{R_{T0,exp}\tau} (S47)$$

where ln 2*/R_T_*_0_*_,exp_* represents the doubling time of regulatory T cell expansion.

## Analysis at the invasive front

## The purpose of the presented analysis is to find an analytical expression that represents the cancer cell density at the inner boundary of the IF. The estimated value is introduced as an input in ABM.

## As stated in [8], deterministic and stochastic scenarios are equivalent in the thermodynamic limit under certain constraints on the reaction system [9,10,11]. Such constraints are fulfilled for elementary reactions, but not for complex systems. Thus, rigorous studies of front propagation in reaction-diffusion problems show noticeable differences between deterministic and stochastic solutions regarding the dynamics of the leading edge, the stability of the system, and the velocity of the front [12,13,14]. To define the inner boundary of the IF, however, we extract information from the deterministic solution of a continuum wave propagation problem described by a partial differential equation [15,13] and from the analysis of its stochastic counterpart [12,13]. The combination of both provides an analytical estimate of the cancer cell density at the inner boundary in terms of the migration and proliferation rates of CSCs and PCs, the number of cancer cells in the system, and the width of the IF conventionally used by pathologists.

## First, we start with the Fisher-Kolmogorov-Petrovsky-Piskunov partial differential equation that includes diffusion and proliferation of cancer cells,

##

$$\frac{d\rho}{dt}=D\nabla^{2}\rho+r\rho\left( 1-\rho\right), (S48)$$

## where *ρ* is the normalized cancer cell density, *D* refers to the cancer cell diffusion coefficient, and *r* is the cancer cell proliferation rate. Numerical analysis performed in [16] quantified how the relation *D/r* regulates the interface width of a tumor at steady state and, more recently, the theoretical study [15] demonstrated analytically that the “wave front” of the tumor at the steady state propagates with a stable and invariant (with respect to time) profile of half-width $w_{1/2}$ such that

##

$$w_{1/2}=4\sqrt{\frac{D}{r}}. (S49)$$

## This half-width is an estimate of the region occupied by the wave front where the cancer cell density gradient is very steep. Nevertheless, qualitative analysis from [15] also shows that the cancer cell density gradients at the transition areas between the saturated core and the wave front and between the wave front and the normal tissue decrease more slowly. Thus, assuming that $w_{1/2}$is, at least, 50% of the half-width, and that *w* is the total width of the wave front, we introduce a multiplicative factor *φ* ~ 2 such that

##

$$w=2 \varphi w_{1/2}=16\sqrt{\frac{D}{r}}, (S50)$$

## to guarantee that the cancer cell density profile decreases from the maximum value at the saturated core to zero at the normal tissue in the region of width *w*. It is important to notice that the wave front is not necessarily the same as approximately 1 mm wide IF defined by pathologists [6,17,18,19,20], but a wider region that includes it. To keep the equations below general, we denote pathologist-defined IF width as *w*_pathol_.

## Now, this analysis is valid for the general cancer cell profile, however, ABM differentiates between CSCs and PCs. Thus, the width of the wave front must be redefined as

##

$$w=16\sqrt{\frac{max\{D_{st},D_{p}\}}{r_{st}}}, (S51)$$

## where *D_st_* and *D_p_* refer to the diffusion coefficients of CSCs and PCs, respectively, and are defined in terms of probability of migration in a time step $\tau$ such that

##

$$D_{cell}=\frac{\tau u_{cell}^{2}}{2 d {v_{m}}^{2}}, cell=st,p (S52)$$

## where *d* is the dimensionality of the grid (*d* = 3). In expression (*S*51), we assume that cells with maximum diffusion coefficient (or migration rate) define the outer boundary of the wave front. Also, in expression (*S*51), we assume that the CSC proliferation rate defines the inner boundary of the wave front since both CSC and PC proliferation mechanisms depend on it (recall equations (*S*2) and (*S*3)). *In vitro* and *in vivo* observations showed that proliferation occurs predominantly near the boundary of the tumor, and that the main mechanism responsible for tumor progression is cell diffusion at the tumor front [21].

## Taking a different approach, we refer to the mathematical analysis from [13] to define the asymptotic behavior of the one-dimensional cancer cell density profile

##

$$\rho\sim\exp\left( -\frac{16 x}{w} \right) for large x \left( at the outer boundary of the wave front \right). (S53)$$

## Using the definition for the density cutoff $\varepsilon$ in a wave front from [12] such that $\rho\sim\varepsilon\sim C_{min}/C$, where $C_{min}$ is the cell number cutoff (we assume $C_{min}$=1 cell) and $C$ is the total number of cancer cells in the whole tumor (~ 10^9^ - 10^11^), we get

##

$$x_{cutoff} \sim\frac{w ln(1/\varepsilon)}{16} , (S54)$$

## where $x_{cutoff}$ is the estimated width of the wave front in a stochastic system according to [12]. A posterior analysis performed in [13] determined that the assumption $\rho\sim\epsilon\sim1/C$ in a deterministic system differs from the stochastic observations and the value of $x_{cutoff}$ gets overestimated. Here, we use the combination of the width estimates of a wave front from [12,13] and [15] to define $x_{cutoff}$ and *w*, respectively, to define the outer boundary of the wave front.

## Now, expression (*S*53) shows that the cancer cell density decreases exponentially at a rate $16/w$ at the outer boundary of the wave front. At the center of the wave front, however, we assumed that the density profile decreased from the maximum value at the saturated core ($\rho=1$) to zero at the normal tissue ($\rho=0$) along at a rate $1/w$. Thus, assuming a similar proportion between the rate at 1 mm from the outer boundary of the wave front and the rate at the outer boundary of the wave front, and linearizing the cancer cell gradient along a wave front of width $x_{cutoff}$, we have

## $\frac{1}{x_{cutoff}}\sim16\frac{\rho_{max,IF}}{w_{pathol}}, (S55)$

## and after substituting expression (*S*54) into (*S*55), the normalized cancer cell density at the inner boundary is expressed as follows

##

$$\rho_{max,IF} \sim\frac{w_{pathol}}{w\ln\left( 1/\varepsilon\right)}. (S56)$$

## The expression $\ln\left( 1/\varepsilon\right)$ can change over time as the tumor grows, but in this study, it is assumed to be constant and approximately equal to 20.

##
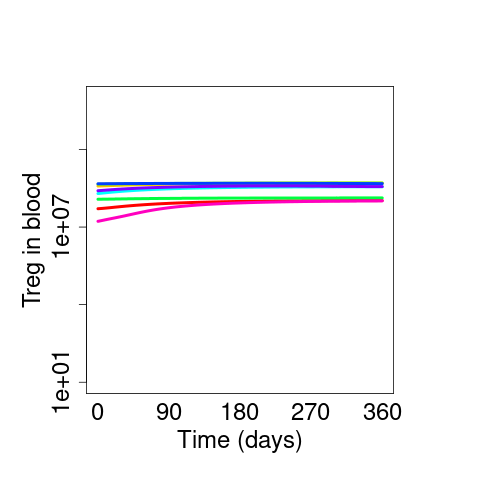

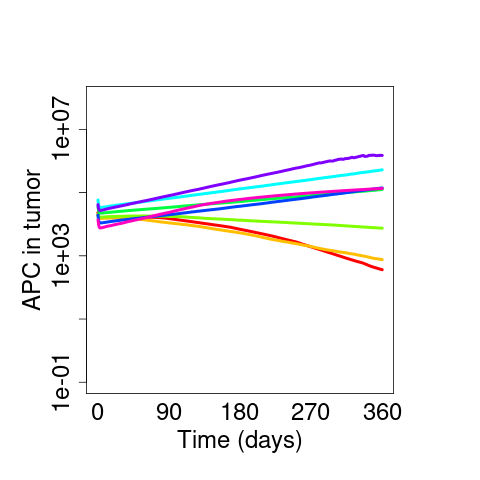

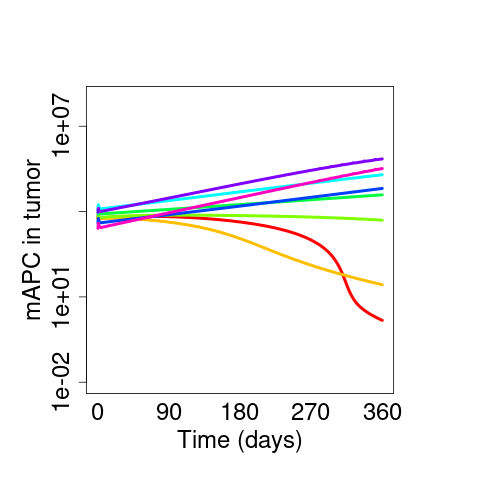

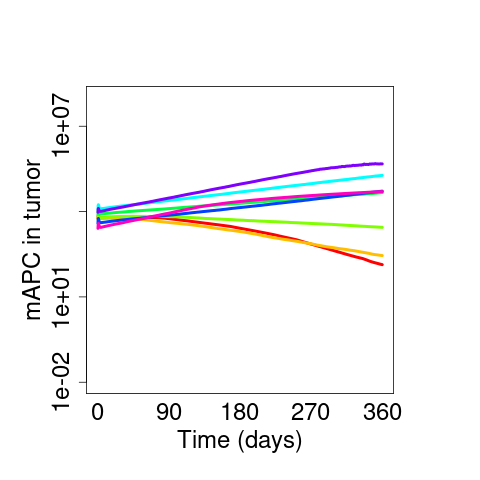

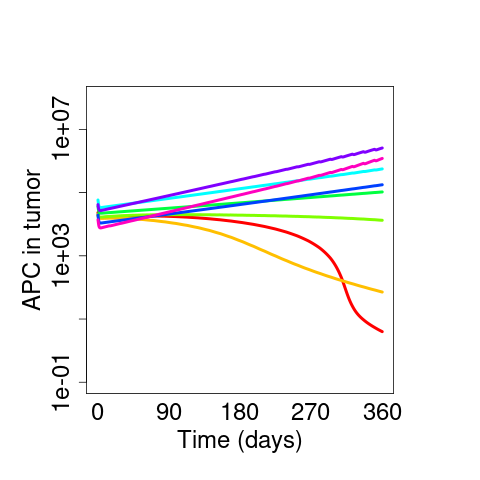

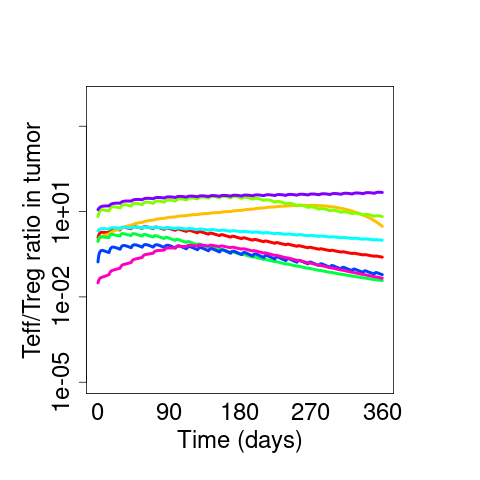

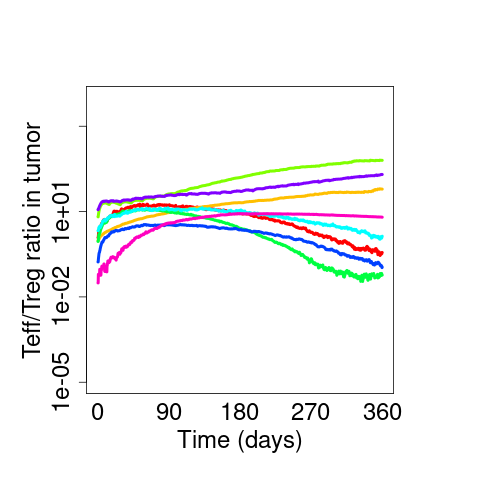

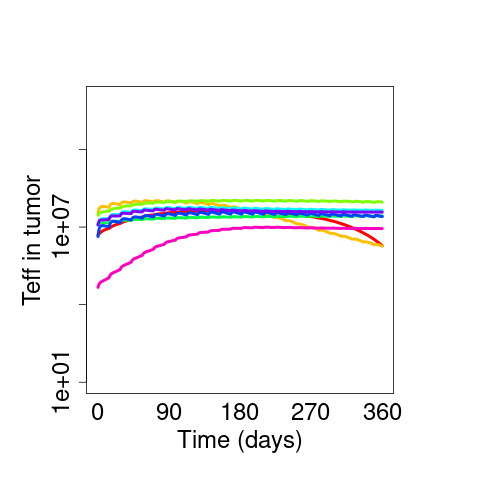

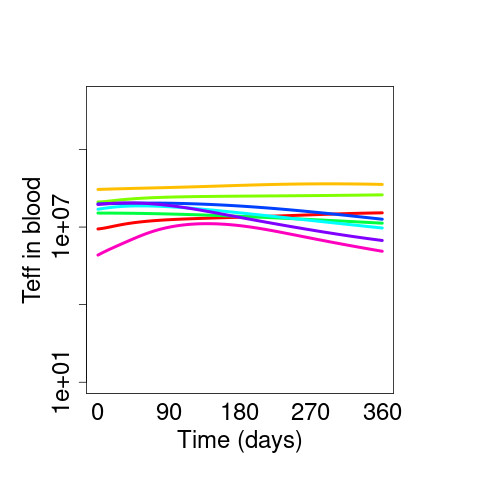

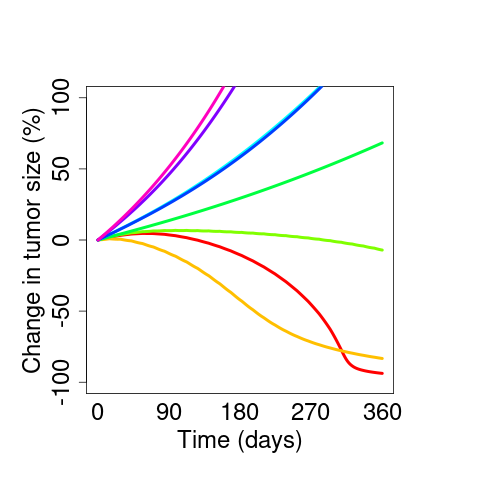

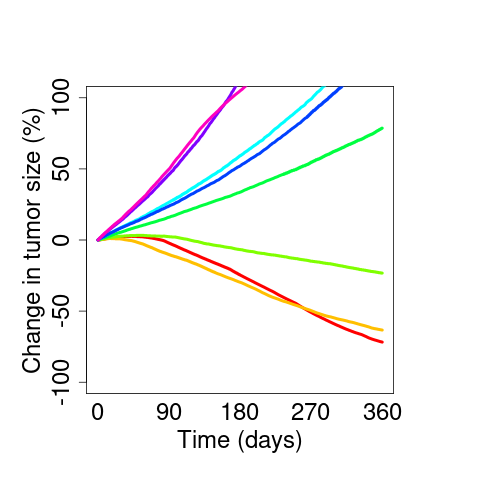

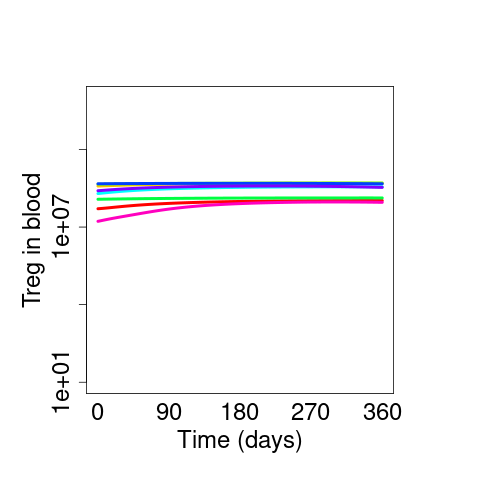

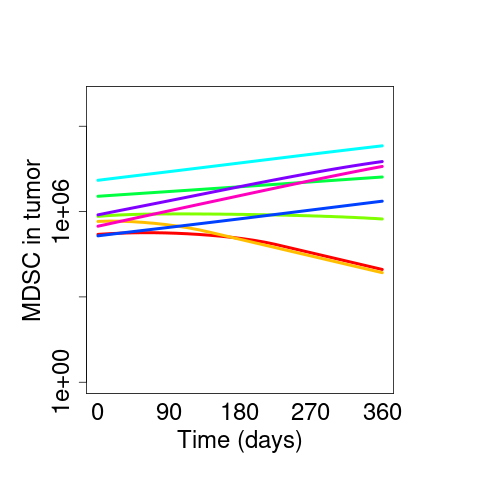

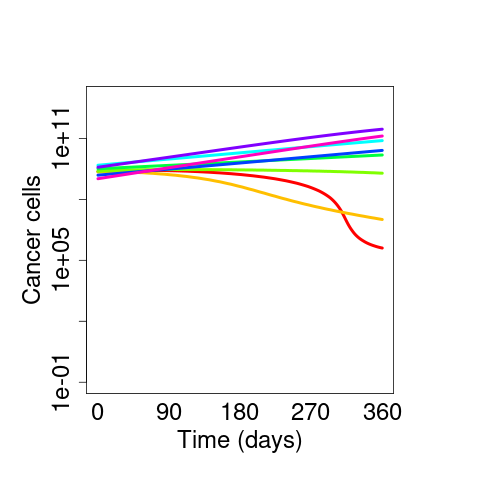

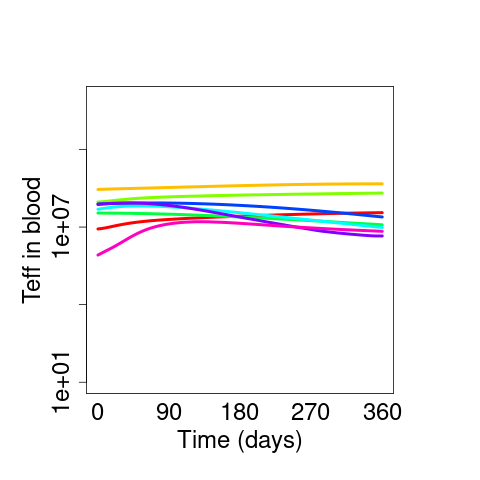

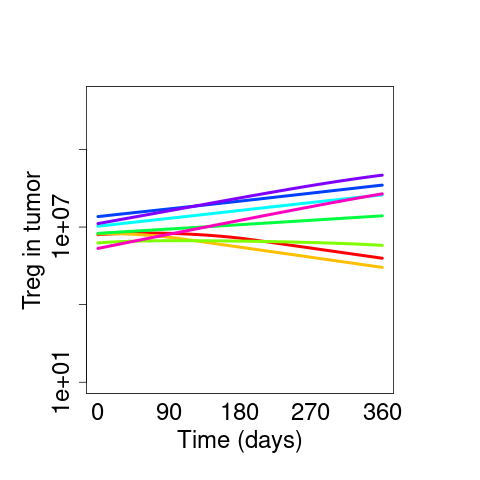

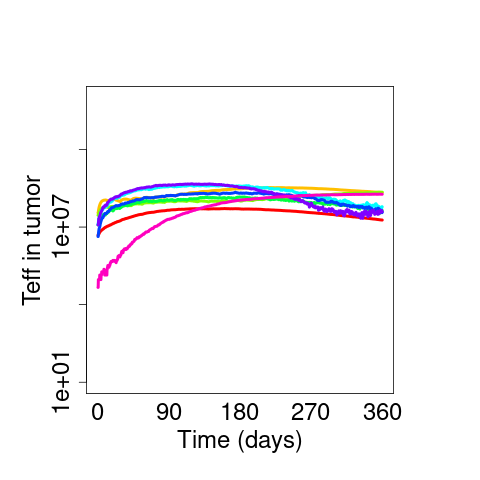

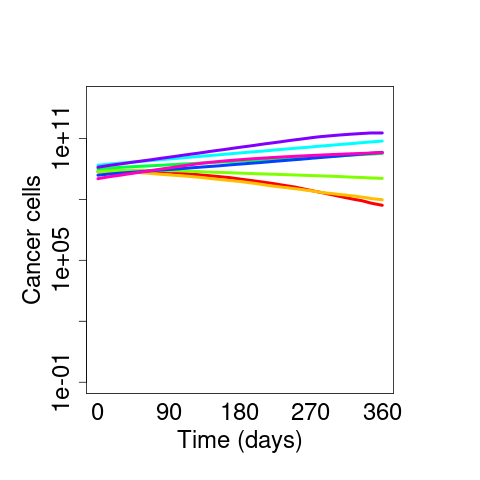

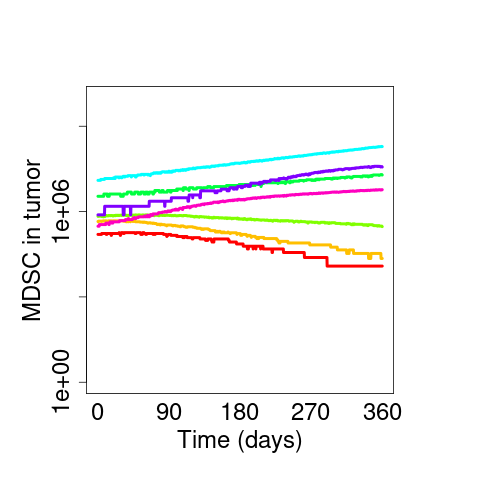
Qualitative comparison of QSP and spatial QSP


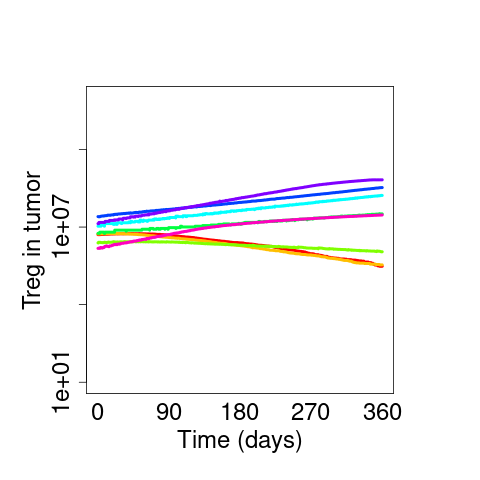


Figure S3: Comparison of QSP (first and third columns) and spatial QSP solutions (second and fourth columns).

# SUPPLEMENTARY MATERIAL

## Growth of approximately spherical tumors


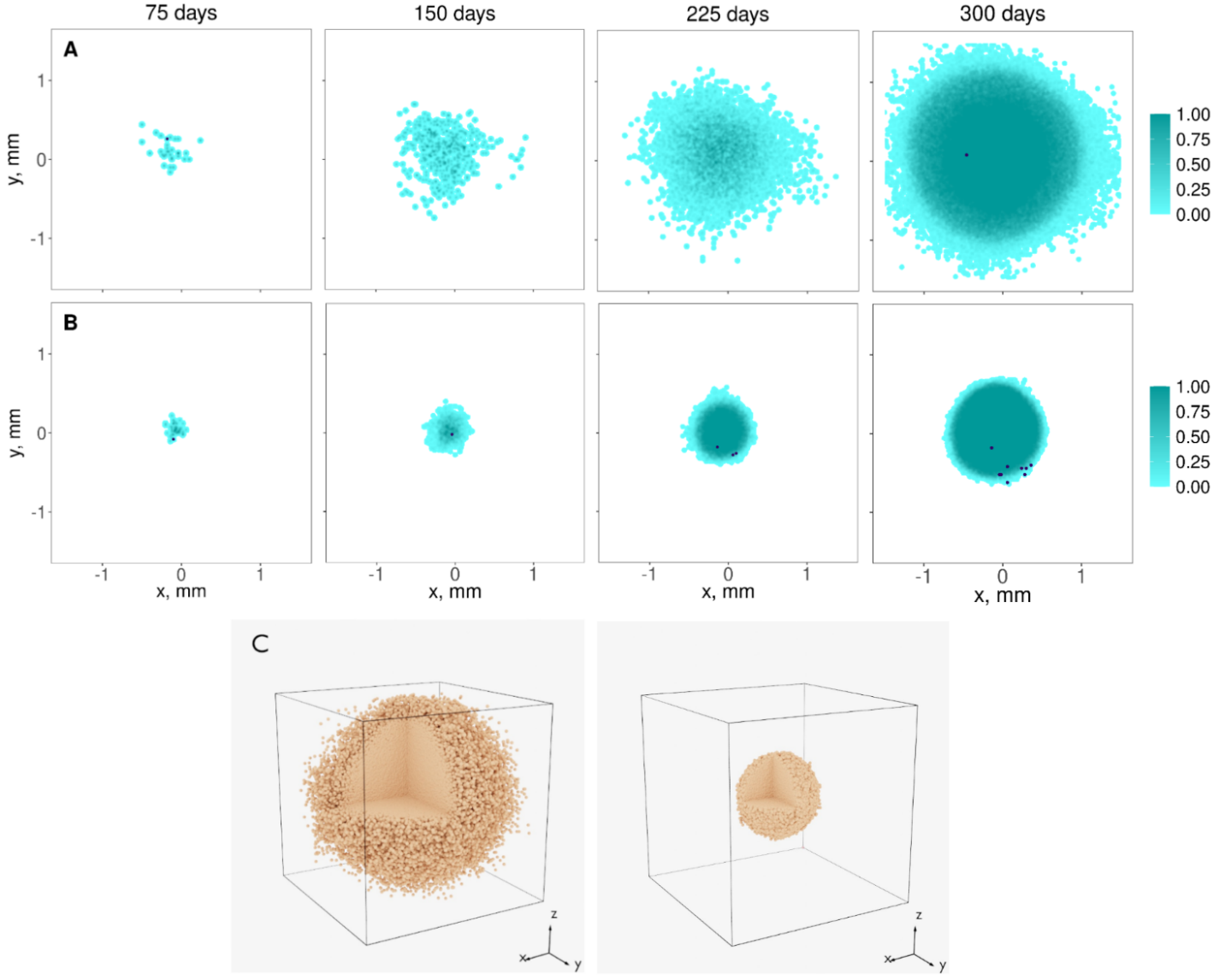


Figure S4: Scaled spatio-temporal evolution of the tumor. This figure is similar to Figure 3 in the main text, but for cases when the tumor is approximately spherical. The relation between cancer stem-like cells (CSCs) and progenitor cells (PCs) migration and proliferation rates, *R*, the asymmetric division probability, *k*, and the maximum number of progenitor cell divisions, *d_max_*, define the evolution in time of the tumor shape and size. In panels A and B, the turquoise scale bar represents the normalized cancer cell density of slices at the center of the tumors with 0.2 mm of thickness every 2.5 months; the dark blue dots are CSCs. In panel C, dark brown and light brown dots represent CSCs and PCs, respectively. Thus, two scenarios are presented: fast proliferation of CSCs and migration of PCs, *R >* 1, high probability of asymmetric division, *k* = 0*.*95, and a large maximum number of progenitor cell divisions, *d_max_* = 18 (panel A); similar migration and proliferation effects, *R ~* 1, *k* = 0*.*95, and *d_max_* = 18 (panel B). The spatial QSP algorithm calculated the evolution of three-dimensional tumors for 10 months starting from one cancer stem-like cell located at the center of the grid. Panel C shows the three-dimensional spatial representation of the tumors from panels A and B after 10 months of growth. All simulations were performed in a 3x3x3 mm grid. The scaling factor is *γ*=1 in all cases.

## Invasive front of approximately spherical tumors without and with treatment


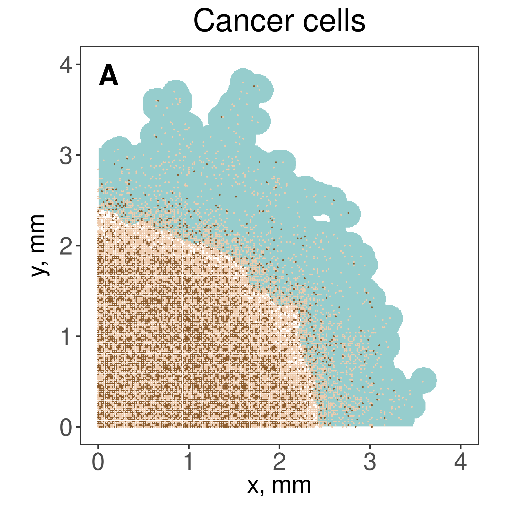

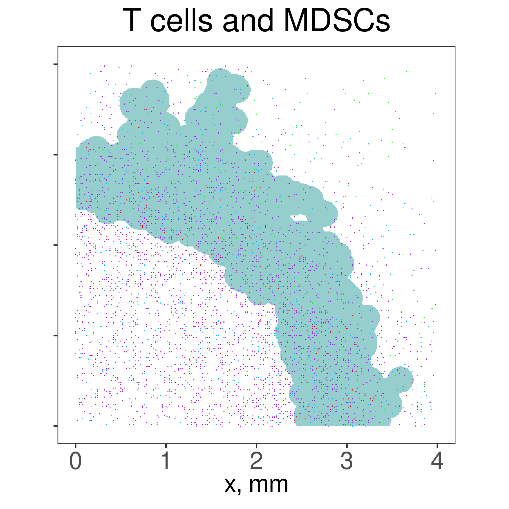

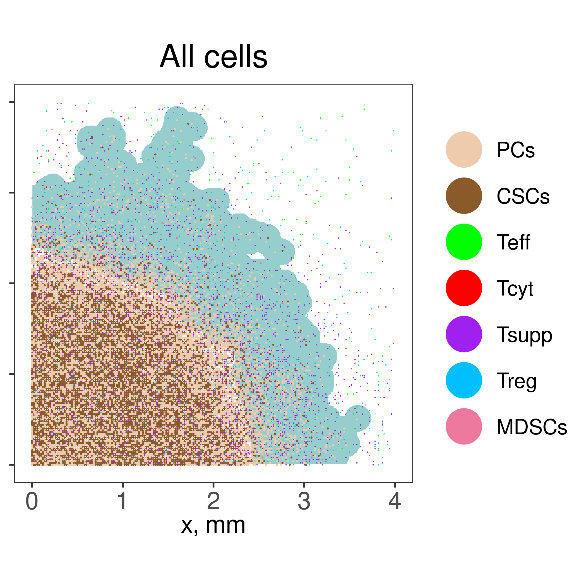

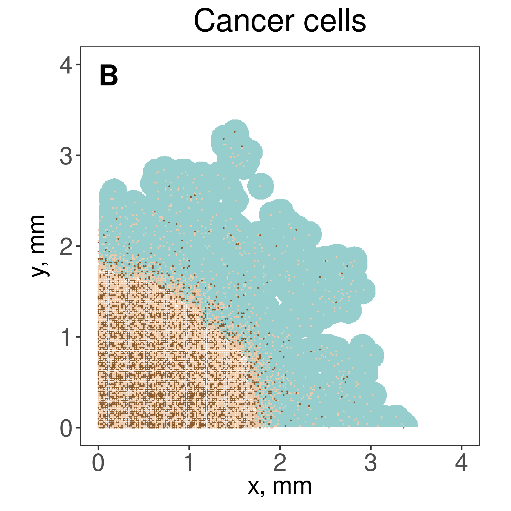

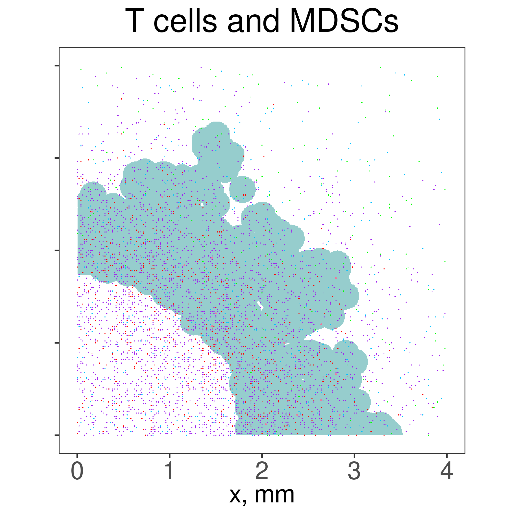

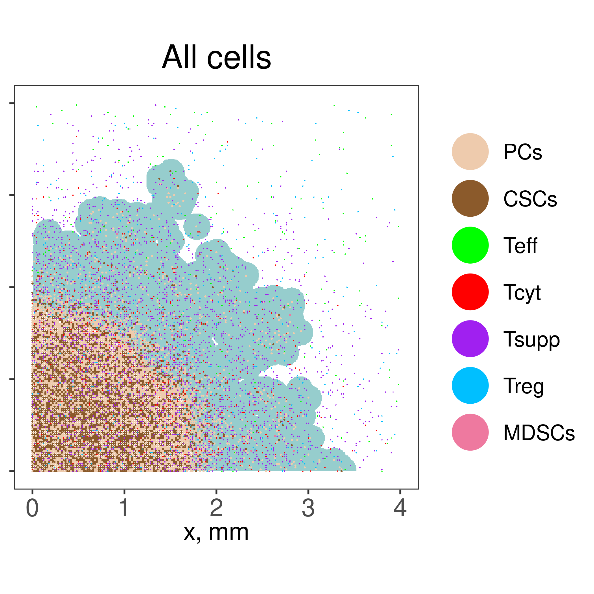

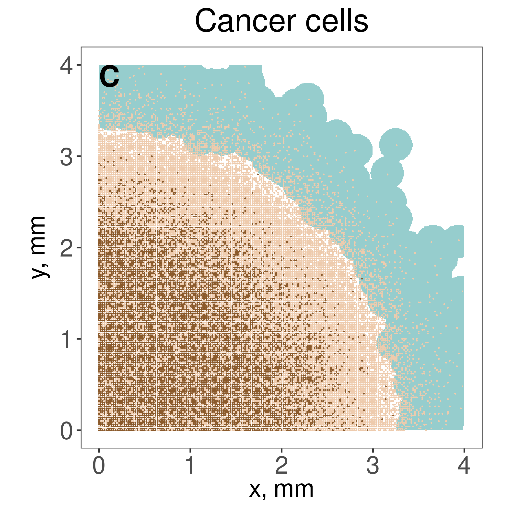

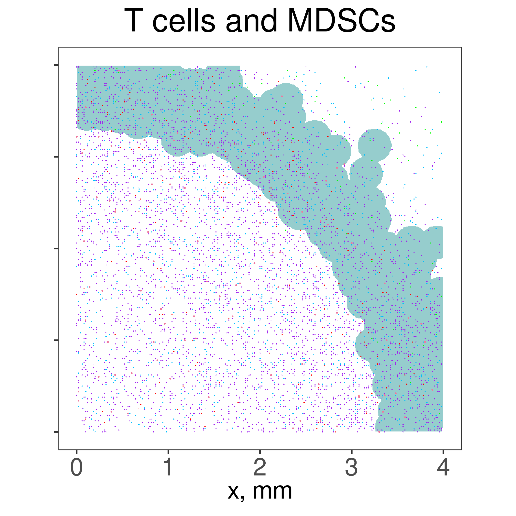

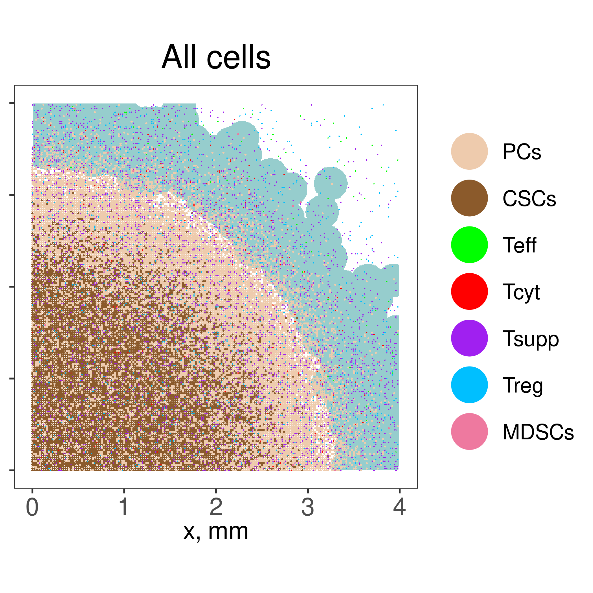

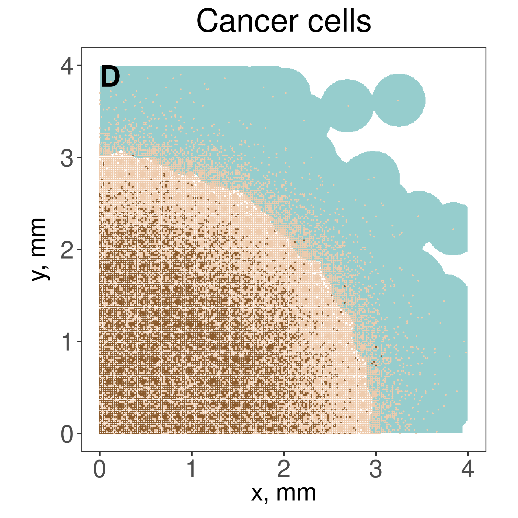

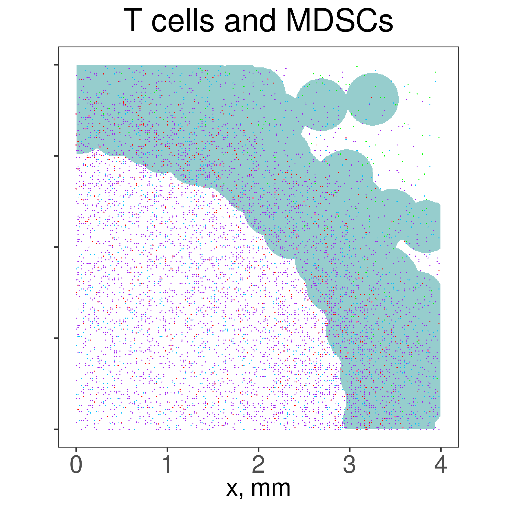

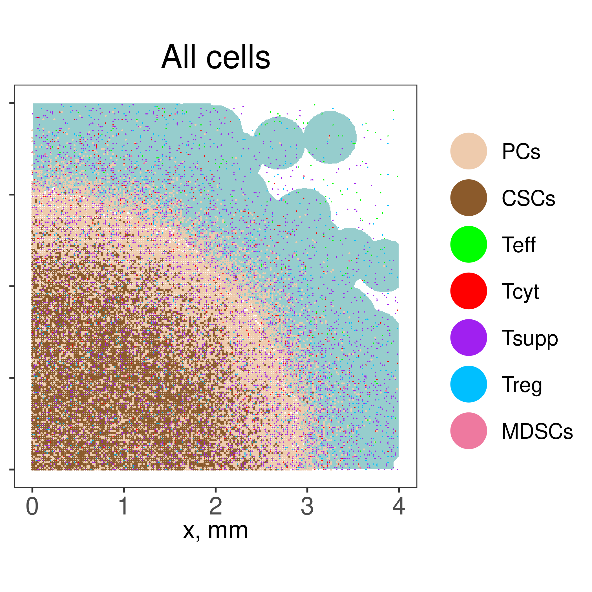


## Figure S5: Spatial representation of cancer cell subtypes (left), CD8+ T cells subtypes, FoxP3+ T cells, and MDSCs (center), and all cells (right) in a section of a tumor slice. The IF region is depicted in pale turquoise. Panels A,B: medium growth case (*k*_C1,growth_ = 0*.*01 day*^−^*^1^) and R = 1, without and with immunotherapy, respectively. Panels C,D: fast growth case (*k*_C1,growth_ = 0*.*015 day*^−^*^1^) and R >> 1 (R = 50), without and with immunotherapy, respectively. The spatial QSP algorithm calculated the evolution of a tumor slice starting from a fraction of a normal distribution of cancer cells. QSP model and ABM are coupled before reaching the point where T cells are recruited and also before the initial tumor diameter condition from the QSP model is met. Thus, no initial T cell spatial distribution is enforced. The figures here presented show cell distributions 6 months after the initial tumor diameter condition is met (with 3 mg/kg nivolumab administered every two weeks in cases with immunotherapy).

## Invasive front in 20 micron thick slices without and with treatment


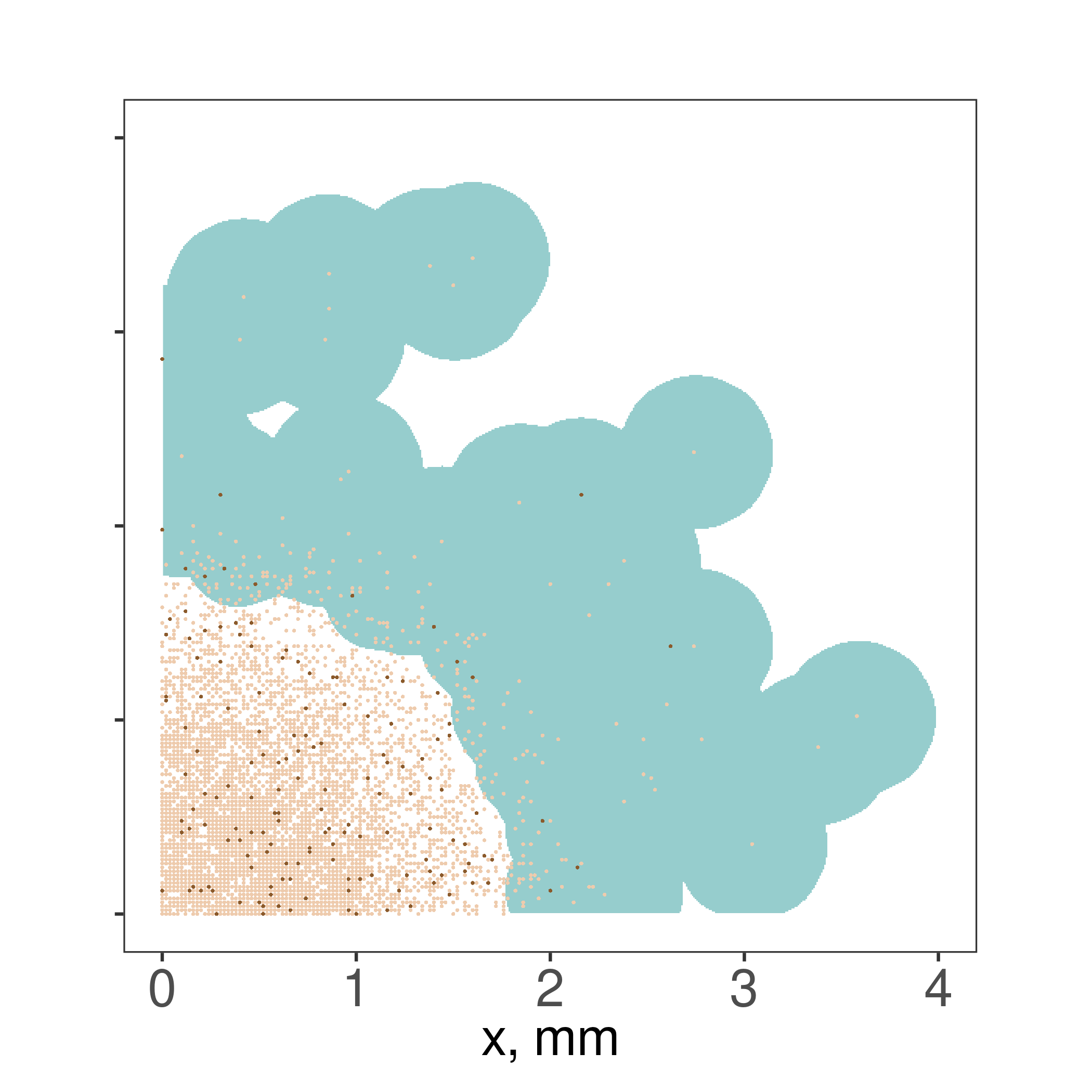

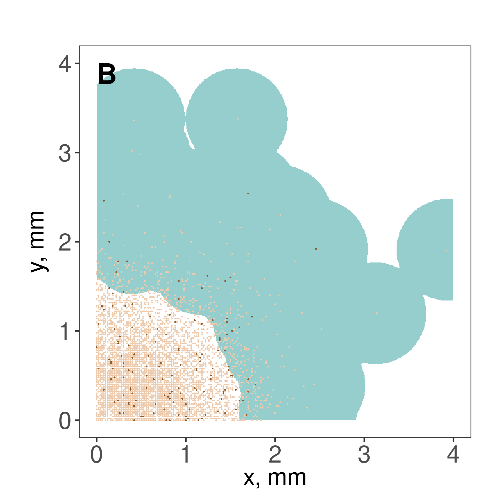

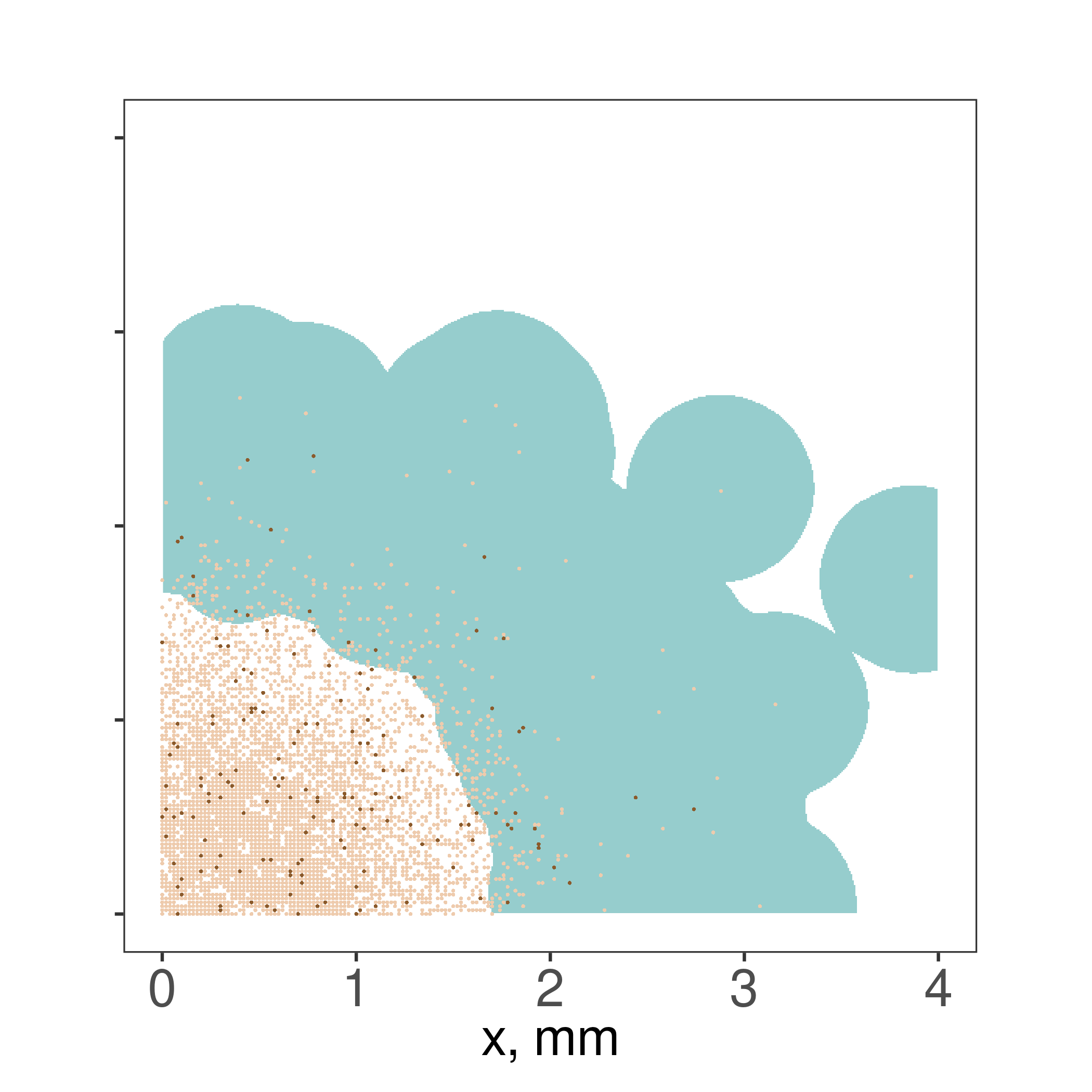

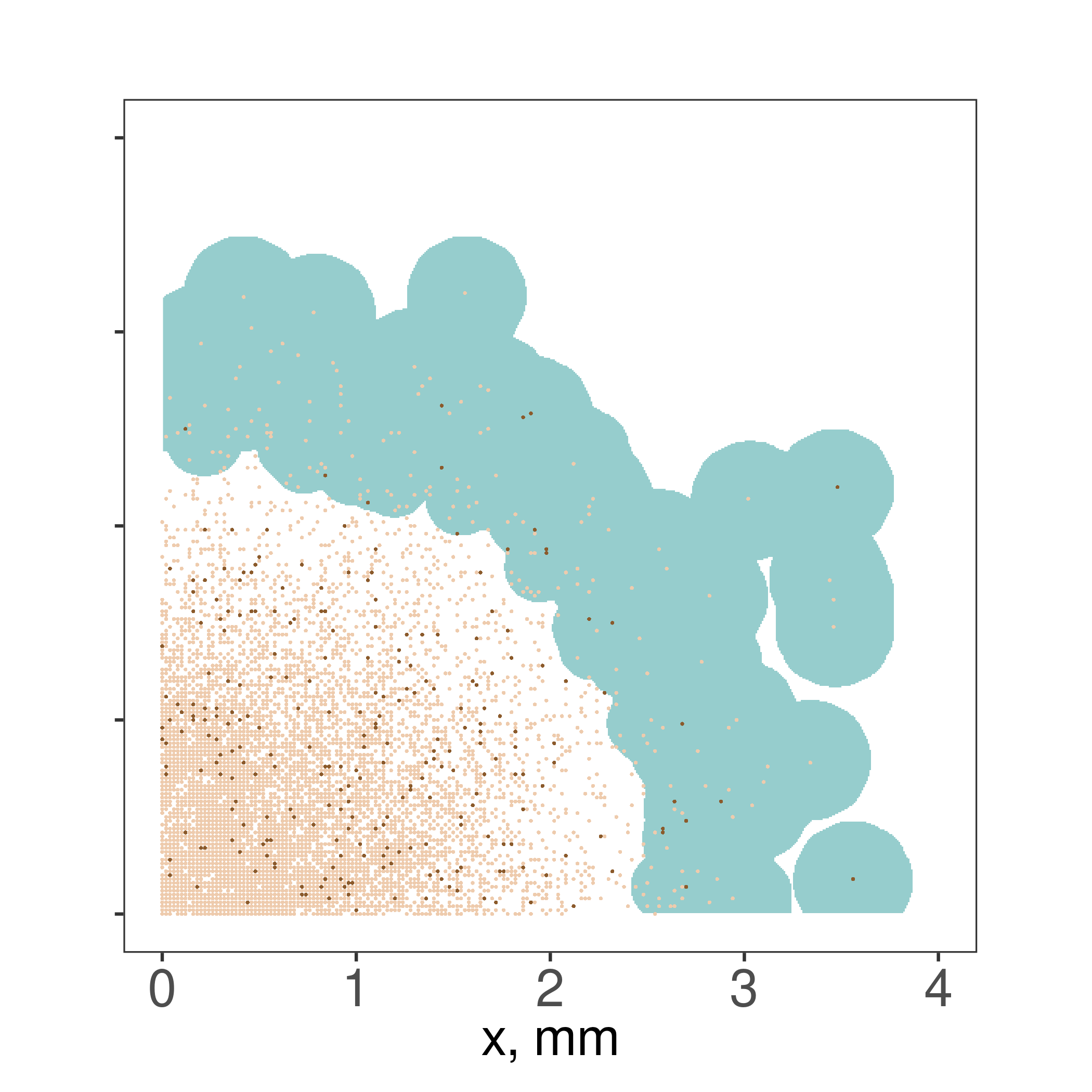

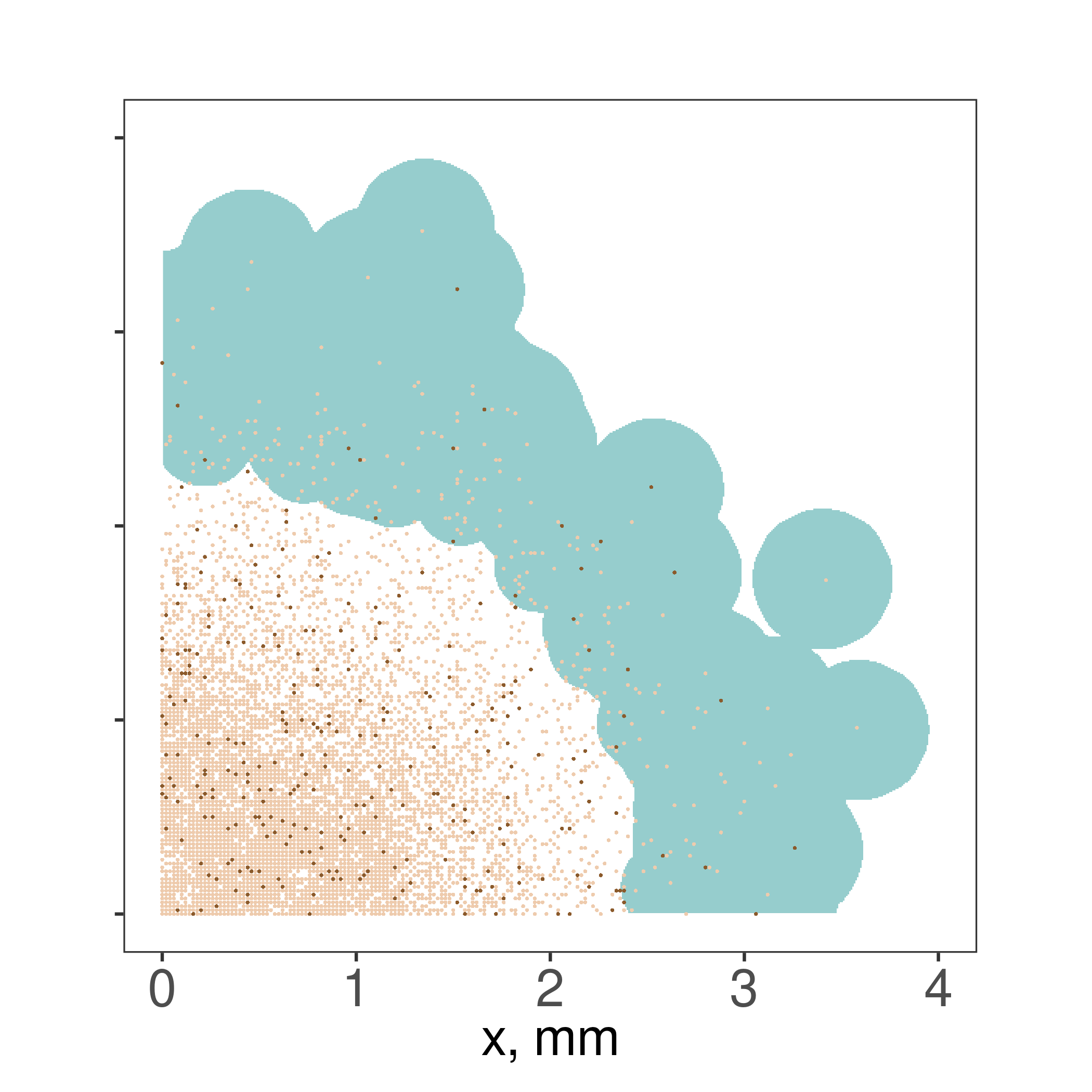

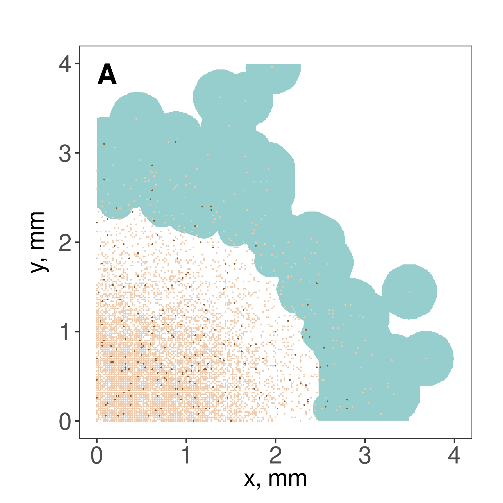


## Figure S6: Spatial representation of cancer cell subtypes in a section of a tumor slice with slow growth (*k*_C1,growth_ = 0*.*005 day*^−^*^1^) and R << 1. The IF region is depicted in pale turquoise. Panel A: Case without immunotherapy. Panel B: Case with immunotherapy. The spatial QSP algorithm calculated the evolution of a tumor slice starting from a fraction of a normal distribution of cancer cells. QSP model and ABM are coupled before reaching the point where T cells are recruited and also before the initial tumor diameter condition from the QSP model is met. Thus, no initial T cell spatial distribution is enforced. The figures here presented show cell distributions 6 months after the initial tumor diameter condition is met (with 3 mg/kg nivolumab administered every two weeks in the case with immunotherapy).

# References

| 1. | Gillespie DT. Exact stochastic simulation of coupled chemical reactions. The Journal of Physical Chemistry. 1977; 81(25): 2340-61. |
| --- | --- |
| 2. | Priami C, Morine MJ. Analysis of biological systems London: World Scientific; 2015. |
| 3. | Marchetti L, Priami C, Thanh VH. Simulation algorithms for computational systems biology Berlin: Springer International Publishing; 2017. |
| 4. | Simoni G, Reali F, Priami C, Marchetti L. Stochastic simulation algorithms for computational systems biology: Exact, approximate, and hybrid methods. Wiley Interdisciplinary Reviews: Systems Biology and Medicine. 2019; 11(6): e1459. |
| 5. | Wang H, Sové R, Jafarnejad M, Rahmeh S, Jaffee E, Stearns V, et al. Conducting a virtual clinical trial in HER2-negative breast cancer using a quantitative systems pharmacology model with an epigenetic modulator and immune checkpoint inhibitors. Frontiers in Bioengineering and Biotechnology. 2020; 8: 141. |
| 6. | Mi H, Gong C, Sulam J, Fertig EJ, Szalay AS, Jaffee EM, et al. Digital pathology analysis quantifies spatial heterogeneity of CD3, CD4, CD8, CD20, and FOXP3 immune markers in triple-negative breast cancer. Frontiers in Physiology. 2020; 11: 583333. |
| 7. | Keren L, Bosse M, Marquez D, Angoshtari R, Jain S, Varma S, et al. A structured tumor-immune microenvironment in triple negative breast cancer revealed by multiplexed ion beam imaging. Cell. 2018; 174(6): 1373-87. |
| 8. | Hahl S, Kremling A. A comparison of deterministic and stochastic modeling approaches for biochemical reaction systems: on fixed points, means, and modes. Frontiers in Genetics. 2016; 7(157). |
| 9. | Kurtz T. The relationship between stochastic and deterministic models for chemical reactions. The Journal of Chemical Physics. 1972; 57(7): 2976-8. |
| 10. | Van Kampen N. Stochastic processes in physics and chemistry Amsterdam: Elsevier; 2007. |
| 11. | Gillespie D. Deterministic limit of stochastic chemical kinetics. The Journal of Physical Chemistry B. 2009; 113(6): 1640-4. |
| 12. | Brunet E, Derrida B. Shift in the velocity of a front due to a cutoff. Physical Review E. 1997; 56(3): 2597. |
| 13. | Kessler D, Ner Z, LM S. Front propagation: precursors, cutoffs, and structural stability. Physical Review E. 1998; 58(1): 107. |
| 14. | Doering C, Mueller C, Smereka P. Interacting particles, the stochastic Fisher–Kolmogorov–Petrovsky–Piscounov equation, and duality.. Physica A: Statistical Mechanics and its Applications. 2003; 325(1-2): 243-59. |
| 15. | Pérez-García VM, Calvo GF, Bosque JJ, León-Triana O, Jiménez J, Pérez-Beteta J, et al. Universal scaling laws rule explosive growth in human cancers. Nature Physics. 2020; 16(12): 1232-7. |
| 16. | Azimzade Y, Saberi A, Sahimi M. Effect of heterogeneity and spatial correlations on the structure of a tumor invasion front in cellular environments. Physical Review E. 2019; 100(6): 062409. |
| 17. | Gong C, Anders RA, Zhu Q, Taube JM, Green B, Cheng W, et al. Quantitative characterization of CD8+ T cell clustering and spatial heterogeneity in solid tumors. Frontiers in Oncology. 2019; 8: 649. |
| 18. | Li X, Gruosso T, Zuo D, Omeroglu A, Meterissian S, Guiot MC, et al. Infiltration of CD8+ T cells into tumor cell clusters in triple-negative breast cancer. Proceedings of the National Academy of Sciences. 2019; 116(9): 3678-87. |
| 19. | Halama N, Michel S, Kloor M, Zoernig I, Benner A, Spille A, et al. Localization and density of immune cells in the invasive margin of human colorectal cancer liver metastases are prognostic for response to chemotherapy. Cancer Research. 2011; 71(17): 5670-7. |
| 20. | Hendry S, Salgado R, Gevaert T, Russell P, John T, Thapa B, et al. Assessing tumor infiltrating lymphocytes in solid tumors: A practical review for pathologists and proposal for a standardized method from the International Immuno-Oncology Biomarkers Working Group. Advances in Anatomic Pathology. 2017; 24(5): 235. |
| 21. | Brú A, Albertos S, Subiza J, García-Asenjo J, Brú I. The universal dynamics of tumor growth. Biophysical Journal. 2003; 85(5): 2948-61. |
