# Supplementary figures and images for: Simulations of tumor growth and response to immunotherapy by coupling a spatial agent-based model with a whole-patient quantitative systems pharmacology model
Source: PLoS Comput Biol. 2022 Jul 22;18(7):e1010254. doi: 10.1371/journal.pcbi.1010254 (PMC9348712; doi:10.1371/journal.pcbi.1010254)

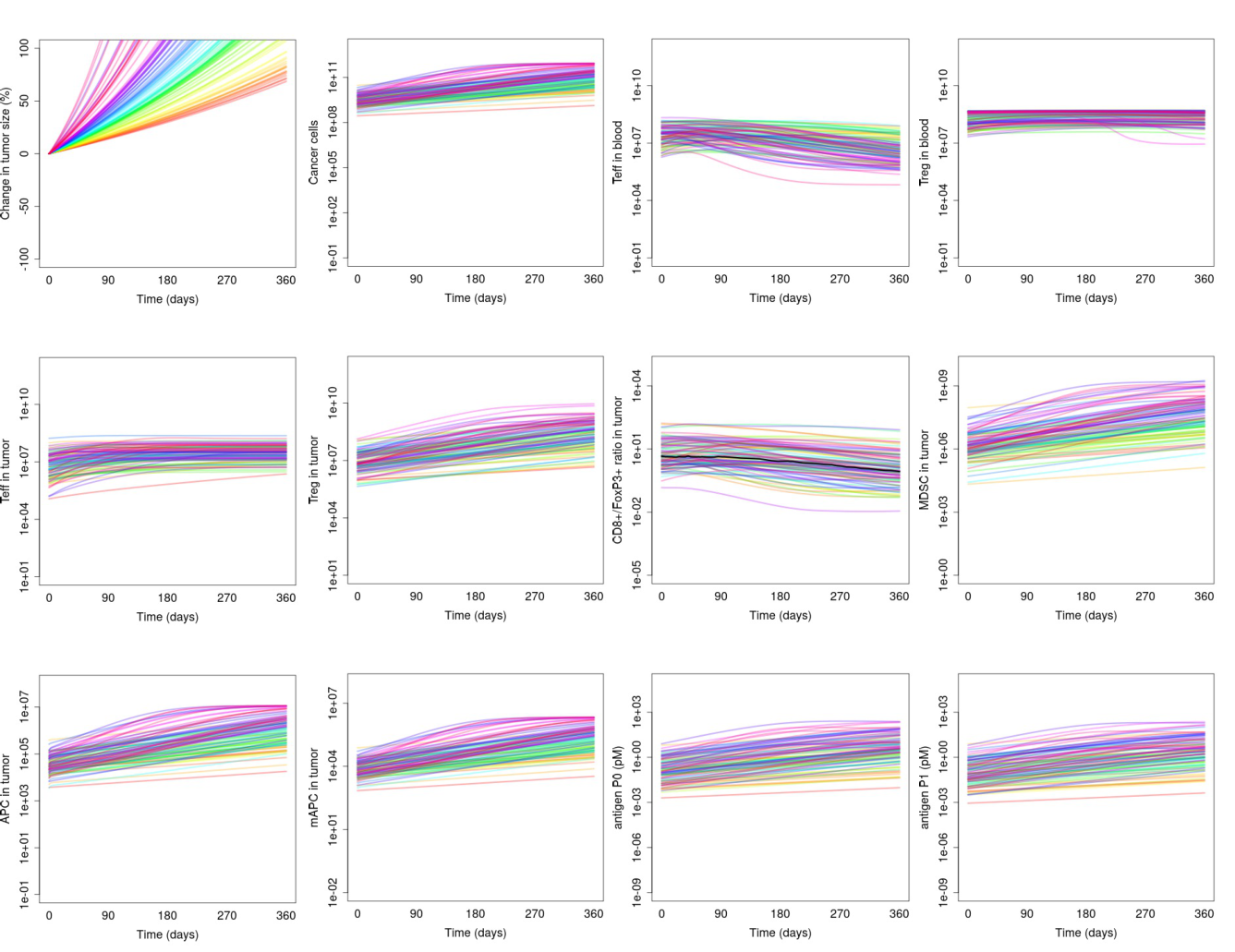

Supplement: S1 Fig — (TIF) [file pcbi.1010254.s002.tif]

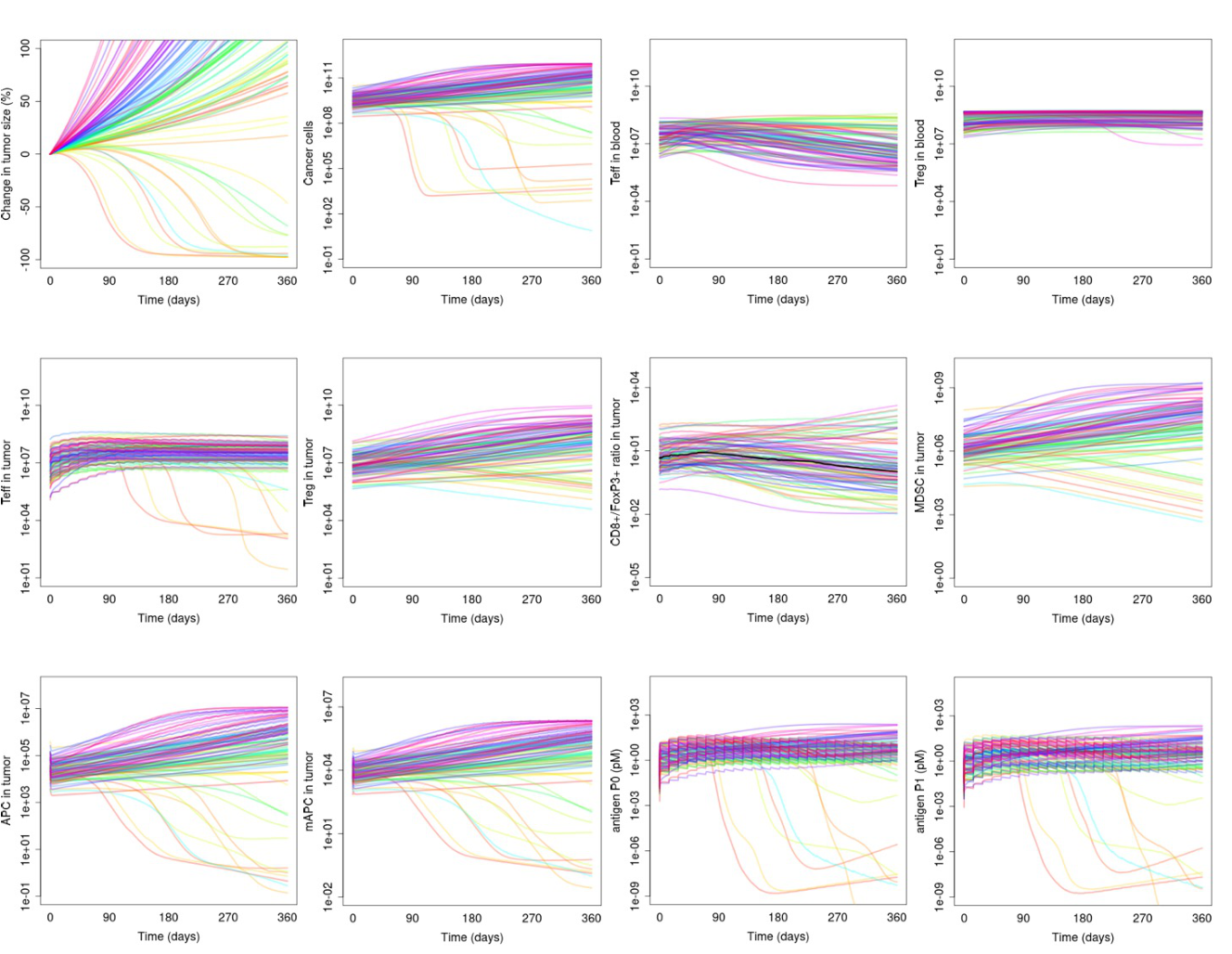

Supplement: S2 Fig — (TIF) [file pcbi.1010254.s003.tif]

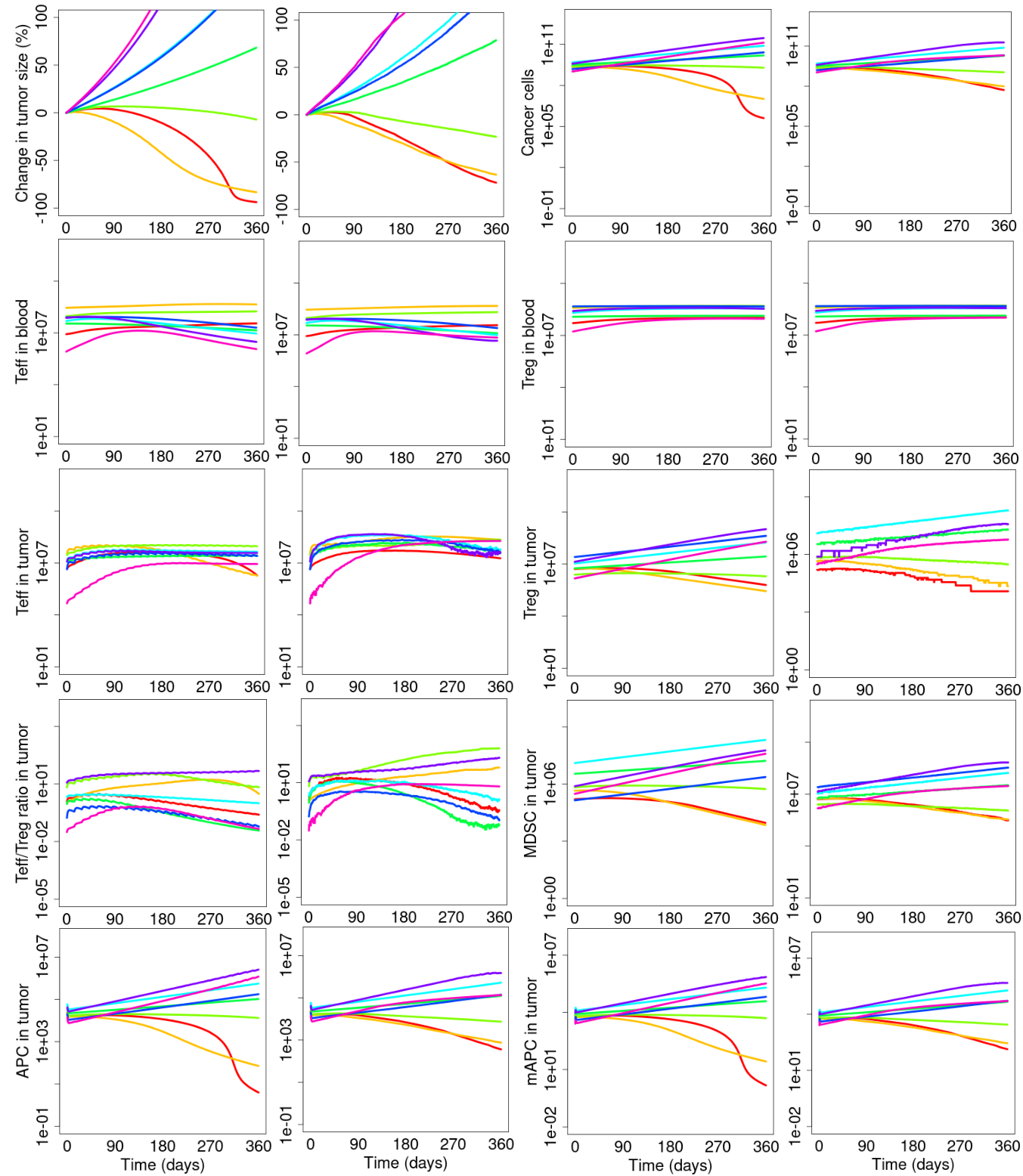

Supplement: S3 Fig — (TIF) [file pcbi.1010254.s004.tif]

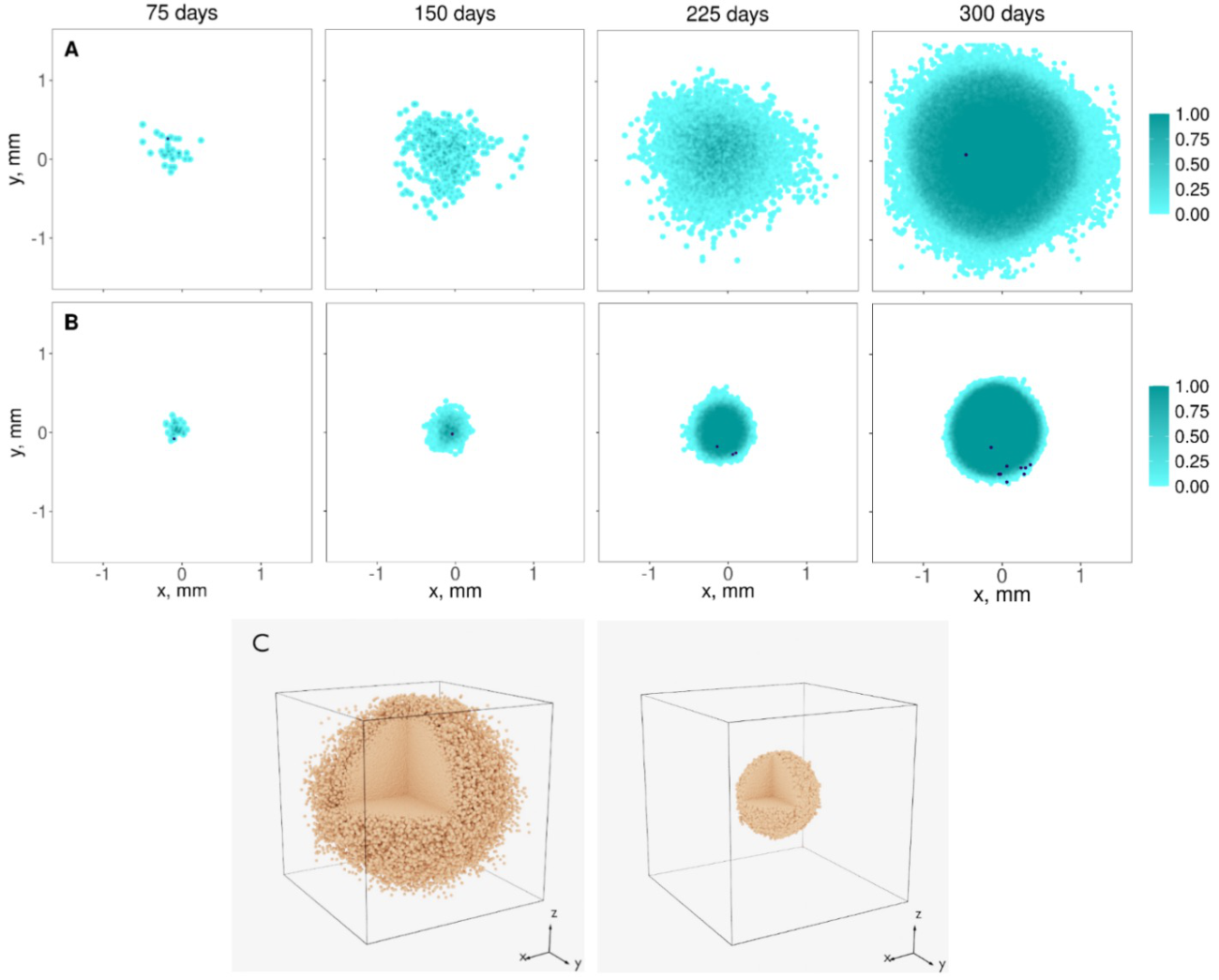

Supplement: S4 Fig — (TIF) [file pcbi.1010254.s005.tif]

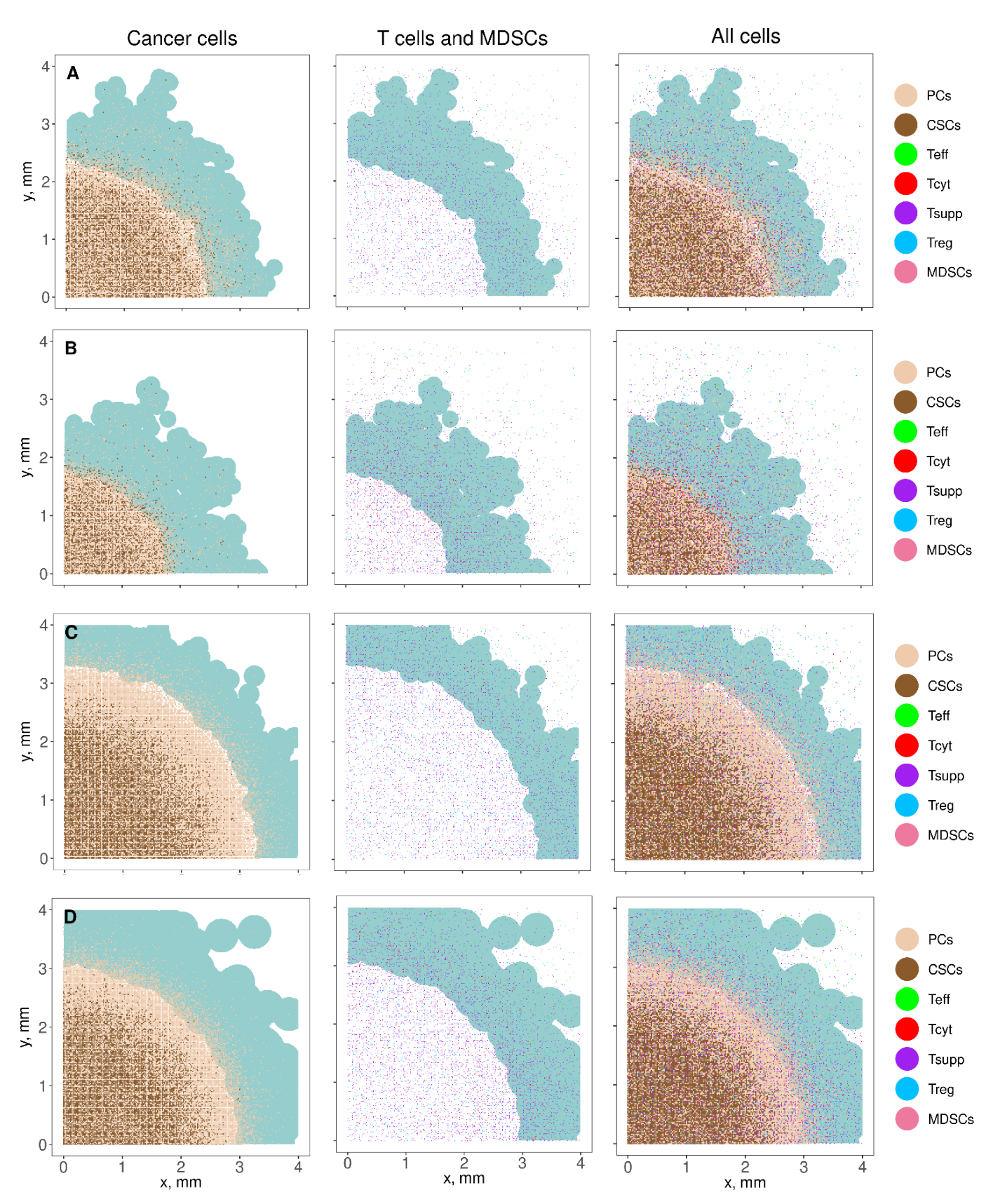

Supplement: S5 Fig — (TIF) [file pcbi.1010254.s006.tif]

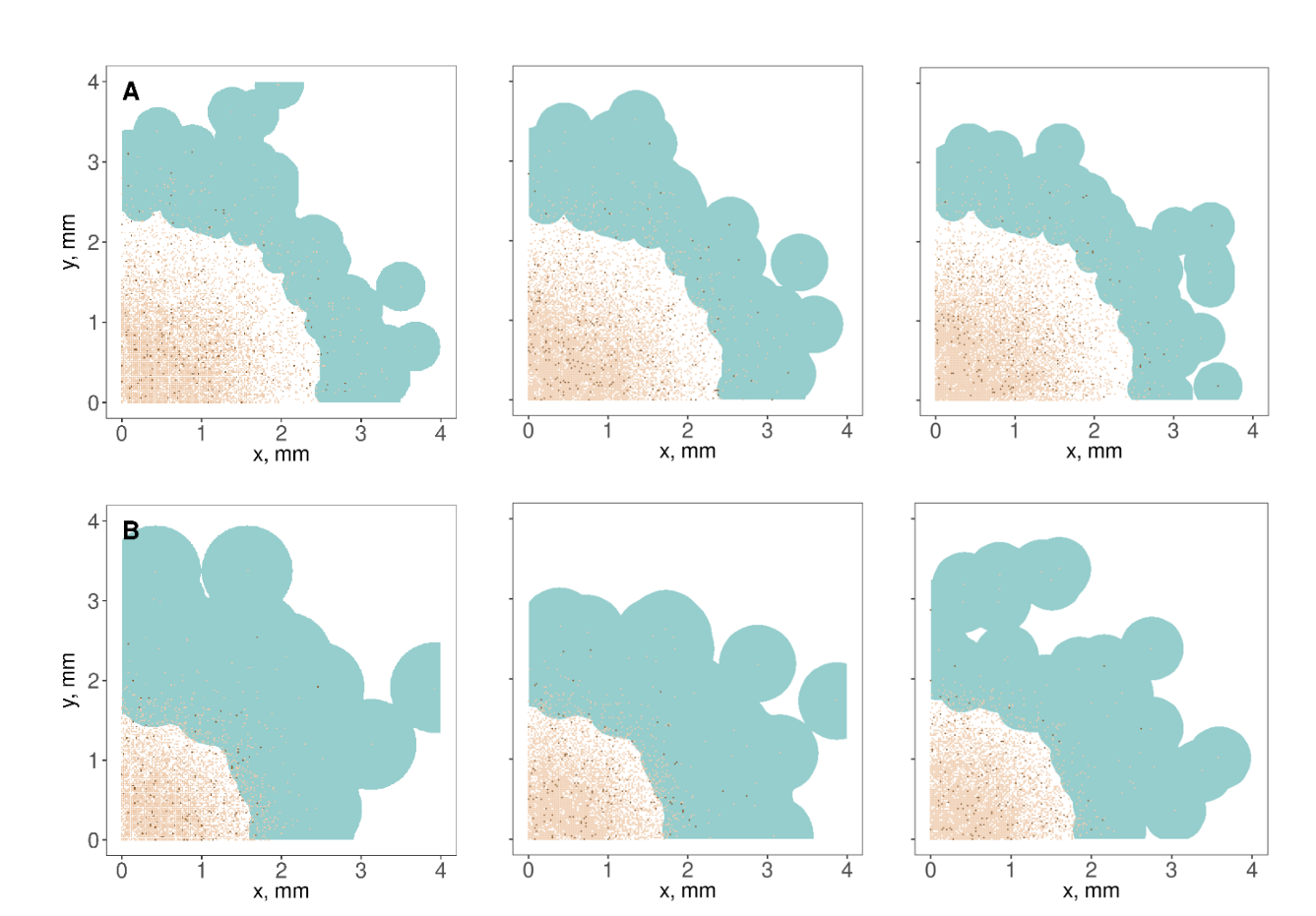

Supplement: S6 Fig — (TIF) [file pcbi.1010254.s007.tif]
